# HLA-B27-associated gut microbiota and amino acid perturbations promote ankylosing spondylitis through M1 macrophage activation

**DOI:** 10.1080/19490976.2026.2630561

**Published:** 2026-02-16

**Authors:** Tianwen Huang, Hang Yang, Lingshu Zhang, Xiangpeng Wang, Ye Chen, Huanzi Dai, Kenji Hashimoto, Yubin Luo, Yaoyu Pu, Yi Liu

**Affiliations:** a Department of Rheumatology and Immunology, West China Hospital, Sichuan University, Chengdu, People's Republic of China; b Department of Rheumatology and Immunology, Daping Hospital, Army Military Medical University, Chongqing, People's Republic of China; c Chiba University Center for Forensic Mental Health, Chiba, Japan; d West China Lecheng Hospital, Sichuan University, Boao, People's Republic of China

**Keywords:** Ankylosing spondylitis, HLA-B27, gut microbiota, metabolome, amino acids

## Abstract

Ankylosing spondylitis (AS) is strongly associated with the human leukocyte antigen B27 (HLA-B27), yet how this genetic risk factor interacts with the gut microbiome remains unclear. We integrated fecal gut microbiota analysis, untargeted metabolomics, and clinical phenotyping in 88 participants, including HLA-B27–positive patients with AS (*n* = 28), HLA-B27–positive healthy controls (*n* = 30), and HLA-B27–negative healthy controls (*n* = 30). HLA-B27 positivity, particularly in AS, was associated with marked alterations in gut microbial composition and metabolic profiles, with forty bacterial species showing progressive disease-related shifts across cohorts. Integrated pathway and metabolomic analyses identified three amino acid–related pathways consistently disrupted in AS: tryptophan metabolism, cysteine metabolism, and pyruvate-centered biosynthesis of branched-chain amino acids, ornithine, and lysine. Correlation network analyses linking differential taxa, metabolites, and clinical indices revealed previously unrecognized microbial and metabolic signatures that robustly distinguished AS from both control groups. To explore causality, fecal microbiota transplantation (FMT) from clinical donors into antibiotic-treated mice recapitulated key disease-relevant features, including impaired intestinal barrier function, systemic inflammation, trabecular bone loss, and polarization of macrophages toward a proinflammatory M1 phenotype. Mechanistic validation identified cinnabarinic acid as a critical microbial-derived metabolite that suppresses M1 macrophage polarization via activation of the aryl hydrocarbon receptor (AhR) pathway and confers protection in the FMT model. Together, these findings support a model in which HLA-B27–associated gut dysbiosis and metabolic reprogramming promote AS pathogenesis through macrophage-mediated inflammation and osteocatabolic signaling, highlighting microbial–metabolic pathways as potential therapeutic targets.

## Background

Ankylosing spondylitis (AS) is a chronic immune-mediated inflammatory disease primarily affecting the axial skeleton and characterized by inflammation at ligament–tendon–bone interfaces (enthesitis). The global prevalence of AS ranges from 0.07% to 0.31%,^1^ with a male-to-female ratio of approximately 2–3:1.^2^ The disease typically begins in the sacroiliac joints and presents with inflammatory low back pain. As AS progresses, persistent inflammation can lead to intervertebral fibrosis, syndesmophyte formation, and ultimately spinal fusion (ankylosis), resulting in severe pain, restricted mobility, and substantial functional impairment.^3^ Despite advances in understanding its clinical course, the etiology of AS remains incompletely defined and is thought to involve a multistage interplay of genetic susceptibility and environmental triggers.

Genetic factors play a central role in AS pathogenesis. Genome-wide association studies (GWAS) estimate that approximately 28% of AS heritability is attributable to genetic variation, with the human leukocyte antigen B27 (HLA-B27) accounting for nearly 20% – the largest single contribution identified to date.^4^^,^^5^ Familial aggregation is prominent, and HLA-B27 is present in up to 90% of patients across diverse ethnic populations.^6^ These observations establish HLA-B27 as one of the strongest genetic risk factors for any polygenic human disease. However, the majority of HLA-B27–positive individuals remain disease free, indicating that genetic predisposition alone is insufficient to drive disease onset and progression.

Accumulating evidence implicates the gut microbiota as a critical environmental factor in AS. Intestinal inflammation is frequently observed in AS, with ileocolonoscopy revealing inflammatory lesions in approximately 50%–70% of patients.^7^ High-throughput 16S rRNA sequencing and metagenomic studies have consistently reported AS-associated alterations in gut microbial composition, including enrichment of *Bacteroides* and *Paraprevotella* and depletion of *Lachnospiraceae*, although some variability exists across cohorts.^8‐10^ Importantly, the influence of HLA-B27 on gut ecology is increasingly recognized. In HLA-B27–transgenic rats, the expression of HLA-B27 induces marked shifts in the intestinal metabolome, and depletion of the gut microbiota by antibiotics substantially attenuates arthritis development.^11^^,^^12^ Together, these findings suggest that HLA-B27 interacts with the gut microbiome to promote AS, while highlighting the need to identify specific microbial and metabolic mechanisms underlying this interaction.

Microbial metabolism represents a key functional link between the gut microbiota and host physiology. Fermentation of dietary fiber and complex carbohydrates by gut microbes produces short-chain fatty acids (SCFAs) and other bioactive metabolites that regulate immune and metabolic pathways. In patients with AS, reduced urinary and fecal levels of acetate, propionate, and butyrate have been reported.^13‐15^ Beyond immune modulation, microbial metabolites also influence bone homeostasis; SCFAs,^16^ insulin-like growth factor-1 (IGF-1),^17^ and purine derivatives^18^ have all been implicated in the regulation of bone formation and resorption. Nevertheless, how HLA-B27-associated changes in gut microbial composition translate into specific metabolic perturbations and downstream effects on immune activation and bone pathology in AS remains poorly understood.

To address these gaps, we performed fecal gut microbiota sequencing and untargeted metabolomic profiling in HLA-B27–positive patients with AS and in HLA-B27–positive and HLA-B27–negative healthy controls. By integrating multiomics data with clinical phenotyping data, we sought to delineate HLA-B27–associated microbial and metabolic signatures and to define their interrelationships within the AS gut ecosystem. In parallel, we employed a fecal microbiota transplantation (FMT) model using human donor samples to assess the causal effects of AS-associated microbiota on intestinal barrier integrity, systemic immune responses, and bone metabolism, with a particular focus on macrophage polarization. Finally, we conducted complementary *in vitro* and *in vivo* validation experiments to investigate the role of the key microbial-derived metabolite cinnabarinic acid (CA) in modulating M1 macrophage activation and to elucidate its underlying molecular mechanism in the AS-FMT model. Collectively, this integrative approach was designed to establish functional links between gut microbial dysbiosis, metabolic reprogramming, and host immune-skeletal pathology in AS.

## Materials and methods

### Subject recruitment

The study protocol was approved by the Biomedical Research Ethics Committee of West China Hospital of Sichuan University (No. 2021-1536), and the written consents were obtained from all the participants according to the Declaration of Helsinki. A cohort of 28 AS patients and 60 HCs, ranging in age from 18 to 65 y, was recruited for the study. The diagnosis of AS was established based on the 1984 modified New York criteria, with all AS patients being HLA-B27-positive. Fecal samples were collected from the patients during their initial visit, before the administration of any disease-modifying antirheumatic drugs (DMARDs), biological agents, glucocorticoids, or Chinese traditional medicine.

Among the 60 HCs, age, gender, and body mass index (BMI) were matched, with 30 individuals testing positive for HLA-B27 and the remaining 30 testing negative. All HCs underwent a comprehensive evaluation that encompasses a thorough medical history inquiry, physical examination, and sacroiliac joint imaging assessment to rule out any spondyloarthritis-related criteria. The exclusion criteria were as follows: (a) having taken antibiotics or probiotics within three months prior to sampling; (b) having undergone digestive endoscopy or surgical procedures within six months prior to sampling; (c) possessing unique dietary habits, such as alcohol or drug abuse, or adherence to a vegetarian diet; (d) being currently pregnant or breastfeeding; and (e) having other rheumatic or autoimmune diseases, major organ system dysfunction, severe infection, cancer, or mental illnesses. A summary of the sociodemographic factors and clinical characteristics is provided (Table 1).^19^

**Table 1.** Demographic and clinical characteristics of the participants.^19^

Characteristics	AS (*n* = 28)	HLA-B27(+) HC (*n* = 30)	HLA-B27(−) HC (*n* = 30)	*P*-value
Age (y), mean ± SD	32.8 ± 7.0	30.9 ± 8.2	35.5 ± 10.2	0.136
Male, *n* (%)	22 (78.6%)	20 (66.7%)	23 (76.7%)	0.536
BMI (kg/m^2^), mean ± SD	22.2 ± 3.3	23.0 ± 3.5	23.6 ± 5.1	0.422
**HLA-B27 status**				
Positive, *n* (%)	28 (100%)	30 (100%)	0 (0%)	-
Negative, *n* (%)	0 (0%)	0 (0%)	30 (100%)	-
Disease duration (y), mean ± SD	6.0 ± 6.7	-	-	-
**Manifestations, *n* (%)**				
Lower back pain	28 (100%)	-	-	-
Peripheral arthritis	9 (32.1%)	-	-	-
Gastrointestinal symptoms*	8 (28.6%)	-	-	-
**Severity of symptoms, mean ± SD**				
CRP (μg/L)	16.1 ± 17.0	-	-	-
ESR (mm/h)	22.8 ± 18.1	-	-	-
BASDAI	3.4 ± 1.9	-	-	-
ASDAS-CRP	2.9 ± 1.0	-	-	-
ASDAS-ESR	2.5 ± 0.9	-	-	-
BASFI	1.6 ± 2.0	-	-	-
Family history, *n* (%)	10 (35.7%)	4 (13.3%)	4 (13.3%)	0.053

AS, ankylosing spondylitis; HC, healthy control; BMI, body mass index; BASDAI, Bath Ankylosing Spondylitis Disease Activity Index; ASDAS, Ankylosing Spondylitis Disease Activity Score; CRP, C-reactive protein; ESR, sedimentation rate; BASFI, Bath Ankylosing Spondylitis Functional Index.

* Refers to symptoms such as abdominal pain, abdominal distension, diarrhea, etc.

### Metagenomic sequencing and data processing

#### Fecal DNA extraction and sequencing

Fecal samples were collected in the morning using sterile tubes and were immediately stored at 4 °C. Within 2 h, they were frozen at −80 °C for subsequent analysis. Genomic DNA was extracted from these samples using the Magnetic Soil and Stool DNA Kit (TIANGEN, Beijing, China), in accordance with the manufacturer's instructions. The quality of the extracted DNA was assessed using 1% agarose gels and the Qubit® 2.0 Fluorometer (Life Technologies, CA, USA). To prepare for PCR amplification, the DNA fragments were subjected to end-polishing, A-tailing, and ligation with full-length adapters. The size distribution of the prepared libraries was then analyzed using the Agilent 2100 Bioanalyzer (Agilent Technologies, Santa Clara, CA, USA), and the quantities were determined using real-time PCR. Finally, the library preparations underwent paired-end sequencing on the Illumina NovaSeq platform (Illumina, San Diego, CA, USA), following the clustering process.

#### Quality control of raw sequences and data analysis

The raw data were subjected to quality filtering using Readfq (V8, https://github.com/cjfields/readfq), and Bowtie2.2.4 was employed to filter out reads originating from the host. Subsequently, the clean data were assembled and analyzed using SOAPdenovo (V2.04) and MEGAHIT (V1.0.4-beta), from which the Scaftigs were obtained. The filtered Scaftigs (those greater than 500 bp) were used to predict the open reading frame (ORF) using MetaGeneMark (V2.10). For ORF prediction, CD-HIT (V4.5.8) was employed to reduce redundancy and to create the unique initial gene catalog. The clean data of each sample were mapped to this initial gene catalog using Bowtie2.2.4, determining the number of reads to which genes were mapped in each sample. To refine the gene catalog for subsequent analysis, genes with read counts fewer than 2 in each sample were filtered out. The resulting gene catalog, referred to as Unigenes, was obtained for further analysis. To assign taxonomic information, the unigenes were analyzed using BLAST searches against sequences of bacteria, fungi, archaea, and viruses extracted from the NCBI NR database using DIAMOND (V0.9.9). Additionally, the unigenes were mapped to the Kyoto Encyclopedia of Genes and Genomes (KEGG) database to acquire functional information, also using DIAMOND.

### Metabolome profiling and data preprocessing

The metabolome analysis was conducted as previously described.^20^ Briefly, fecal metabolites were extracted using a solution of 400 μl of methanol to water (7:3, V/V). The mixture, consisting of 20 mg of fecal sample and the extraction solution, was vortexed for 3 min and then ultrasonicated at 40 kHz for 30 min at 4 °C. After centrifugation at 13,000 × *g* for 15 min at 4 °C, the supernatant was transferred to sample vials for liquid chromatography‒mass spectrometry (LC‒MS) analysis. To maintain quality control, an equal aliquot of each sample (10 μl) was combined to create a pooled sample, referred to as the quality control (QC) sample. Metabolome profiling was carried out using an ultra-performance liquid chromatography system (Shimadzu, Kyoto, Japan) coupled with a TripleTOF 6600 mass spectrometer (AB Sciex, Framingham, MA, USA). The raw data were converted to the mzML format using ProteoWizard for subsequent peak extraction, alignment, and integration. After eliminating features detected in less than 80% of any set of samples, a total of 4083 metabolite features in positive mode and 3810 metabolite features in negative mode were obtained. These features were then combined for further statistical analysis.

### Experimental animals

Male C57B/6 mice (8 weeks old, 20–25 g) were acclimatized and maintained in a specific-pathogen-free (SPF) environment with controlled temperature (23 ± 1 °C) and a 12-h light/dark cycle, with free access to SPF-grade food and water.^21^ All procedures performed in this study were in compliance with the guidelines approved by the Institutional Animal Care and Use Committee of West China Hospital, Sichuan University (Approval No. 20220221071). Following deep anesthesia induced by isoflurane, the mice were euthanized by cervical dislocation to alleviate suffering.

### Schedule of fecal microbiota transplantation (FMT) and sample collection

Broad-spectrum antibiotics (ABX: ampicillin, neomycin sulfate, and metronidazole, each 1 g/L) dissolved in drinking water were provided ad libitum to the male C57BL/6 mice for 14 consecutive days (day 1–14), as previously reported.^22^ The solution was renewed every 2 or 3 d. From days 15 to 28, the animals underwent FMT derived from AS patients or from HLA-B27-positive or -negative HCs. The fecal samples used for transplantation were obtained from our study cohort, detailed donor characteristics are provided in Table 1 and Table S1. To minimize bias caused by inter-donor variability, fecal samples from multiple donors were mixed at equal weight to create a standardized inoculum for each transplantation group.^23^ A total of six male C57BL/6 mice per group were used in the FMT experiments. All the mice received identical antibiotic pretreatments and subsequently received fecal suspensions from the donor mixture of their assigned groups. Fresh fecal samples from the mice were collected on day 28. The mice were euthanized on day 49, and the serum, peripheral blood cells, spleen, small intestine, colon, bone marrow, tibia, hind paw, and lumbar vertebra were harvested for downstream analyzes.

In the CA treatment experiment, antibiotic pretreatment and FMT from AS patients were administered as described above. Beginning on day 29, the mice received daily gavage of saline, CA (10 mg/kg), or a combination of CA and CH-223191 (10 mg/kg each; MedChemExpress, NJ, USA) for 21 d, followed by euthanasia on the following day (day 50).

### 16S ribosome RNA sequencing

The fecal samples from the mice were each placed into individual sterile tubes immediately after defecation and were stored at −80 °C prior to use. The DNA extractions from the fecal samples and 16S ribosome RNA sequencing analyzes were performed as reported previously.^23^

The alpha diversity was used to analyze the complexity of species diversity for each sample using four indices: the Shannon, Chao l, ACE and observed ASV indices. For beta diversity of the gut microbiota, PCoA of the ASV level was performed using analysis of similarities (ANOSIM) in the R package vegan.

### Gene expression analysis by quantitative real-time PCR

Total RNA was extracted from the small intestine, bone marrow, and hind paw tissues, reverse-transcribed into complementary DNA, and quantified by quantitative real-time PCR as reported previously.^21^ Each sample was analyzed in triplicate, and the relative gene expression was normalized to *Gapdh*. Data analysis was performed using the 2^-ΔΔCt^ method. The primer sequences are listed in Table S2.

### Western blot analysis

Following a previously described method,^21^ protein extracts from the small intestine, hind paw and cultured cells were prepared in Laemmli buffer and quantified (BCA assay). Aliquots of 50 µg of protein were denatured and separated on 10% SurePAGE™ gels prior to electrophoretic transfer to PVDF membranes. The membranes were blocked (5% goat serum, 1 h) and then probed overnight at 4 °C with specific primary antibodies: ZO-1 (1:1000, Affinity Bioscience, #AF5145), TRAP (1:1000, Affinity Bioscience, #DF6989), *p*-IKKα/β (1:1000, Affinity Bioscience, #AF3013), IKKα/β (1:500, Affinity Bioscience, #AF6014), *p*-IκBα (1:1000, Affinity Bioscience, #AF2002), IκBα (1:500, Affinity Bioscience, #AF5002), *p*-p65 (1:1000, Affinity Bioscience, #AF5875), p65 (1:500, Affinity Bioscience, #AF5006), *β*-actin (1:20000, Affinity Bioscience, #AF7018), and *α*-tubulin (1:20000, Affinity Bioscience, #AF7010). Post-incubation with an HRP-conjugated goat anti-rabbit IgG secondary antibody (1:10000, Affinity Bioscience, #S0001), the protein bands were visualized by ECL Prime and analyzed using a ChemiDoc™ Touch system with Image Lab 3.0 software (Bio-Rad).

### Immunofluorescent staining

Paraffin-embedded small intestine tissues were sectioned for immunofluorescence staining of F4/80 and CD86. Following deparaffinization, antigen retrieval was conducted using EDTA buffer (pH 8.0) with microwave heating for 10 min. After cooling to RT, sections were washed three times with PBS (5 min each). Non-specific sites were blocked with 5% goat serum in PBS for 1 h at RT. The sections were then incubated overnight at 4 °C with the following primary antibodies: anti-F4/80 rat monoclonal (1:1000, Servicebio, #GB12027) and anti-CD86 rabbit monoclonal (1:200, Servicebio, #GB13585). After PBS washes, sections were incubated with secondary antibodies (FITC-conjugated goat anti-rat IgG and Cy3-conjugated donkey anti-rabbit IgG) for 1 h at RT in the dark. Following washes, nuclei were stained with DAPI for 10 min. Autofluorescence was reduced using a quenching reagent (5 min), followed by a 10-min water rinse. The sections were then mounted with antifade medium. The slides were imaged with a VS200 whole-slide scanner and analyzed using OlyVIA 4.1 software.

### TRAP staining

TRAP staining was performed using a commercial kit (Servicebio, #G1050-50T) according to the manufacturer's instructions. Deparaffinized sections were preincubated with purified water at 37 °C for 2 h in a humidified chamber. Subsequently, the TRAP working solution was applied and incubated for 20–30 min at 37 °C in the dark. After washing with distilled water, nuclei were counterstained with 0.5% methyl green for 5 min. Finally, the sections were rinsed, dehydrated through a graded ethanol series, cleared in xylene, and mounted with neutral resin.

### Micro-CT

The right tibia and lumbar vertebrae (L1–L5) were harvested from each mouse and fixed in 4% paraformaldehyde. Subsequently, the samples were scanned using a NEMO® Micro CT imaging system. The scanning parameters were set as follows: current 60 μA, voltage 90 kV, lateral field of view 30 mm, axial field of view 10.2 mm, and a total of 4000 frames per scan. Following acquisition, the images were reconstructed using Cruiser reconstruction software. Two- and three-dimensional analyzes were then performed with Avatar 3.0 software. A region of interest (ROI) was defined in the tibia starting at 1.0 mm distal to the growth plate and extending 0.5 mm longitudinally. For vertebral analysis, the L4 vertebra was selected as the ROI. Quantitative parameters of trabecular and cortical bone were assessed within these ROIs for statistical evaluation.

### Culture and M1 polarization of Bone Marrow-Derived Macrophages (BMDMs)

Bone marrow cells were aseptically harvested from the femurs and tibias of 6–8-week-old male C57BL/6 mice. After erythrocyte lysis, cells were resuspended in complete macrophage differentiation medium consisting of DMEM/F12 supplemented with 10% fetal bovine serum (FBS), 1% penicillin–streptomycin, and 20 ng/mL macrophage colony-stimulating factor (M-CSF). The cells were counted and plated at a density of 2 × 10⁶ cells/mL in appropriate culture vessels, then maintained at 37 °C in a humidified atmosphere containing 5% CO₂. Half of the culture medium was replaced every 2–3 d. After 5–6 d of differentiation, adherent mature macrophages (M0 phenotype) were obtained.

For M1 polarization, M0 macrophages were stimulated with lipopolysaccharide (LPS; 100 ng/mL) and interferon-*γ* (IFN-γ; 20 ng/mL). The cells were harvested at 0.5, 6, and 24 h after stimulation for downstream analyzes. For the CA treatment experiments, CA (100 μM) was added simultaneously with the M1-polarizing stimuli. To inhibit aryl hydrocarbon receptor (AhR) signaling, the cells were pretreated with the AhR antagonist CH-223191 (10 μM) for 1 h prior to the addition of CA.

### Statistical analysis

All the statistical analyzes were conducted using R software (version 4.3.1). Demographic variables were compared by one-way ANOVA with Tukey's post hoc test for multiple comparisons, while gender differences were assessed using the chi-square test. Nonparametric data from metagenomic and metabolomic analyzes were evaluated with the Kruskal–Wallis rank-sum test, followed by Dunn's test for pairwise comparisons. For the animal studies, the data are expressed as the mean ± SEM. Statistical significance was determined routinely using one-way ANOVA followed by post hoc Tukey's multiple comparison tests. For all comparisons, a *P*-value of less than 0.05 was considered statistically significant.^20^^,^^21^

The *α*-diversity was computed with the R vegan package. For *β* diversity, Bray‒Curtis distances were calculated and visualized via principal coordinates analysis (PCoA) employing the ade4 and vegan packages. Differentially abundant bacterial taxa (from species to higher taxonomic ranks) were identified by linear discriminant analysis effect size (LEfSe) with significance criteria set at an LDA score > 2.0 and *P* < 0.05.^20^ Kruskal–Wallis rank-sum test was employed to identify differential KEGG orthology (KO) genes among the three groups (*P* < 0.05). Subsequently, differential microbial metabolic activities were revealed through KEGG pathway maps.

Orthogonal partial least squares discriminant analysis (OPLS-DA) was conducted in R with the ropls package. To identify differentially abundant metabolites in two-group comparisons, a composite threshold was applied, requiring a variable importance in projection (VIP) greater than 1, a fold change (FC) either greater than 2 or less than 0.5, and a false discovery rate (FDR) below 0.05. The results were visualized in a volcano plot constructed with ggplot2. Associations between altered taxa and metabolites across all subjects were evaluated using Spearman's correlation analysis with the psych package.^20^ The heatmap was produced using the heatmap package, while the Cytoscape software was employed to assess and depict the correlations between species, metabolites, and clinical indexes using a circular layout. The random forest package and pROC package were utilized to construct the random forest analysis and receiver operating characteristic curve (ROC), respectively.

## Results

### Characteristics of the study population

**Figure 1.**
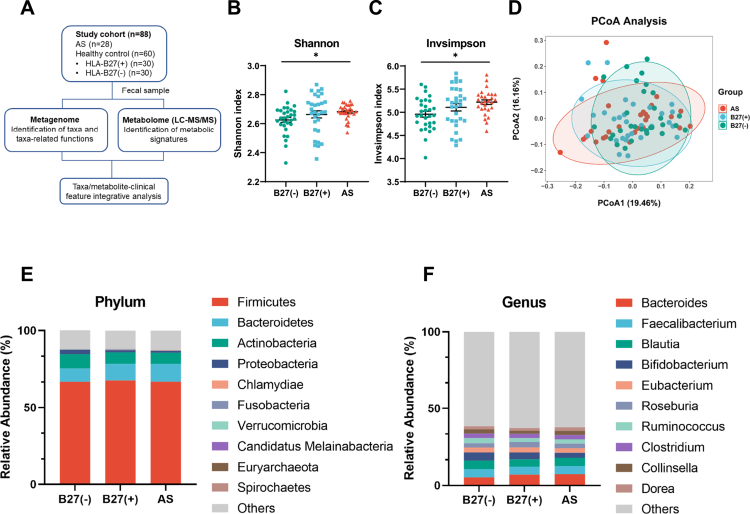
Gut microbiome alteration among AS, B27(+) and B27(−) groups. (A): Overview of the study design. (B) and (C): Alpha diversity measured by the Shannon and Invsimpson index was highest in AS (*n* = 28) compared with B27(+) (*n* = 30) and B27(−) (*n* = 30) (Kruskal‒Wallis test). (D): Principal coordinate analysis (PCoA) revealed that the gut microbial composition of AS was significantly different from that of B27(−), while no difference was found among the three groups (Bray–Curtis distance, ANOSIM, B27(−) vs AS, *P* = 0.027; B27(−) vs B27(+) vs AS, *P* = 0.161; B27(+) vs AS, *P* = 0.631; B27(−) vs B27(+), *P* = 0.442). Additionally, the top 10 relative abundance of bacterial taxa at the (E) phylum and (F) genus levels were presented. *P < 0.05.

We analyzed 88 fecal samples from three cohorts: AS patients (AS, *n* = 28), HLA-B27-positive HCs (B27(+), *n* = 30), and HLA-B27-negative HCs (B27(−), *n* = 30) (Figure 1A). Demographic and clinical characteristics – including gender, age, and disease indices – are summarized in Table 1^19^ and Table S1. Age, sex, and body mass index (BMI) did not differ significantly among groups. All the participants reported rice as their staple food, and the distribution of taste preferences and dietary habits were comparable across the three groups (Table S3). Within the AS cohort, disease activity was generally high (ASDAS-CRP: mean ± SD, 2.9 ± 1.0). In addition to low back pain, gastrointestinal symptoms were reported by eight patients (28.6%).

### Altered composition of the gut microbiota among AS, B27(+) and B27(−) groups

We profiled gut microbial differences across the AS, B27(+), and B27(–) cohorts using metagenomic sequencing. Alpha diversity was higher in the AS group than in both the control groups by Shannon and Inverse Simpson indices (*P* = 0.0419 and *P* = 0.02, respectively; Figure 1B–C), whereas the Chao1 index did not differ (*P* = 0.6112; Figure S1A). Principal coordinate analysis (PCoA) based on Bray–Curtis distances revealed that although the overall comparison among the three groups was not statistically significant (ANOSIM, *P* = 0.161), pairwise analysis revealed a significant difference between AS patients and HLA-B27–negative individuals (*P* = 0.027) (Figure S1B). Notably, the HLA-B27–positive healthy group exhibited an intermediate microbial composition, which is consistent with a gradient pattern progressing from HLA-B27–negative healthy controls to HLA-B27–positive healthy controls and ultimately to AS patients (Figure 1D).

**Figure 2.**
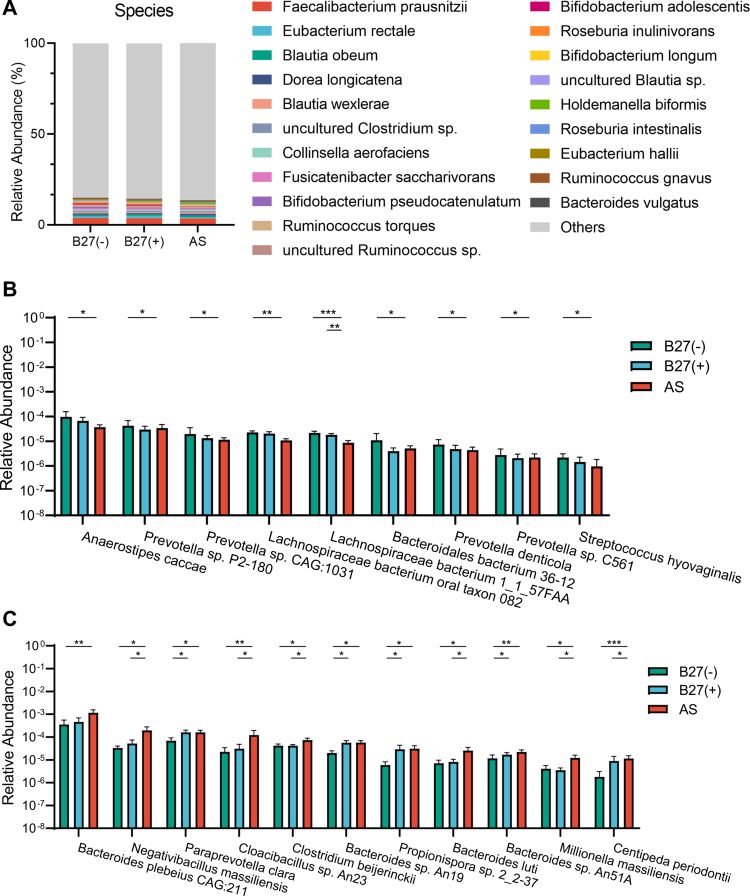
Alterations of the microbial species among AS, B27(+) and B27(−) groups. (A): The top 20 relative abundance of microbial species. Bar plots showed the relative abundance of species decreased (B) and increased (C) in AS compared with B27(+) and B27(−). *P < 0.05, **P < 0.01, ***P < 0.001 as determined by the Kruskal‒Wallis post hoc test.

Given the hypothesized impact of HLA-B27 on gut ecology and AS pathogenesis, we focused on taxa showing progressive changes across groups (Kruskal–Wallis test). At the phylum level, the ten most abundant phyla are shown in Figure 1E, with *Synergistetes* enriched in AS versus B27(–) (Figure S1C). At the genus level (Figure 1F), AS showed higher *Acidaminococcus* (Figure S1D) and *Bilophila* (Figure S1E) compared with B27(–); *Paraprevotella* was increased in both AS and B27(+) relative to B27(–) (Figure S1F). At the species level, the top 20 most abundant species are depicted in Figure 2A. In total, 40 species differed significantly in AS (31 increased, 9 decreased). The AS group exhibited significantly lower relative abundances of *Anaerostipes caccae*, *Prevotella sp. P2-180*, and *Lachnospiraceae bacterium oral taxon 082*, among others, when compared to the B27(−) group (Figure 2B). There was also a notable decrease in *Lachnospiraceae bacterium 1_1_57FAA* in the AS group relative to both the B27(+) and B27(−) groups (Figure 2B). Species such as *Negativibacillus massiliensis, Bacteroides luti*, and *Centipeda periodontii* were found to be enriched in the AS group as opposed to both the B27(+) and B27(−) groups (Figure 2C). Additionally, *Paraprevotella clara*, *Bacteroides sp. An19*, and *Bacteroides sp. An51A*, among others, were enriched in both the AS and B27(+) groups (Figure 2C). Furthermore, species including *Bacteroides plebeius*, *Bilophila sp. 4_1_30*, and *Bilophila wadsworthia* showed a significantly increased abundance in the AS group when compared to the B27(−) group (Figure S1G–H).

To identify group-specific microbial biomarkers, we performed LEfSe (Figure S2). In the AS group, we detected different distributions of two types of bacteria (e.g. *Prevotellaceae*, *Caudovirales*), while in the B27(+) group, four types of bacteria were identified (e.g. *Sporomusaceae*, *Sutterellaceae*, *Campylobacterales* and *Epsilonproteobacteria*) (Figure S2A). In total, 27 mixed-level phylotypes were identified as potential microbial markers, including 13 taxa (e.g. *Prevotellaceae*, *Paraprevotella*, *Negativibacillus massiliensis,* etc.). For the AS group, 7 taxa (e.g. *Clostridium sp. CAG:7*, *Eubacterium sp. CAG:274*, *Sutterellaceae*, etc.) for the B27(+) group and 7 taxa (e.g. *Blautia sp. CAG:237*, *Clostridium sp. AT4*, *Clostridium sp. L2-50,* etc.) for the B27(−) group (Figure S2B).

Collectively, these data indicate that the HLA-B27 status is associated with shifts in the gut microbial composition and that dysbiosis is most pronounced in HLA-B27–positive patients with AS relative to HLA-B27–positive healthy controls.

### Alterations in fecal metabolites among AS, B27(+) and B27(−) groups

**Figure 3.**
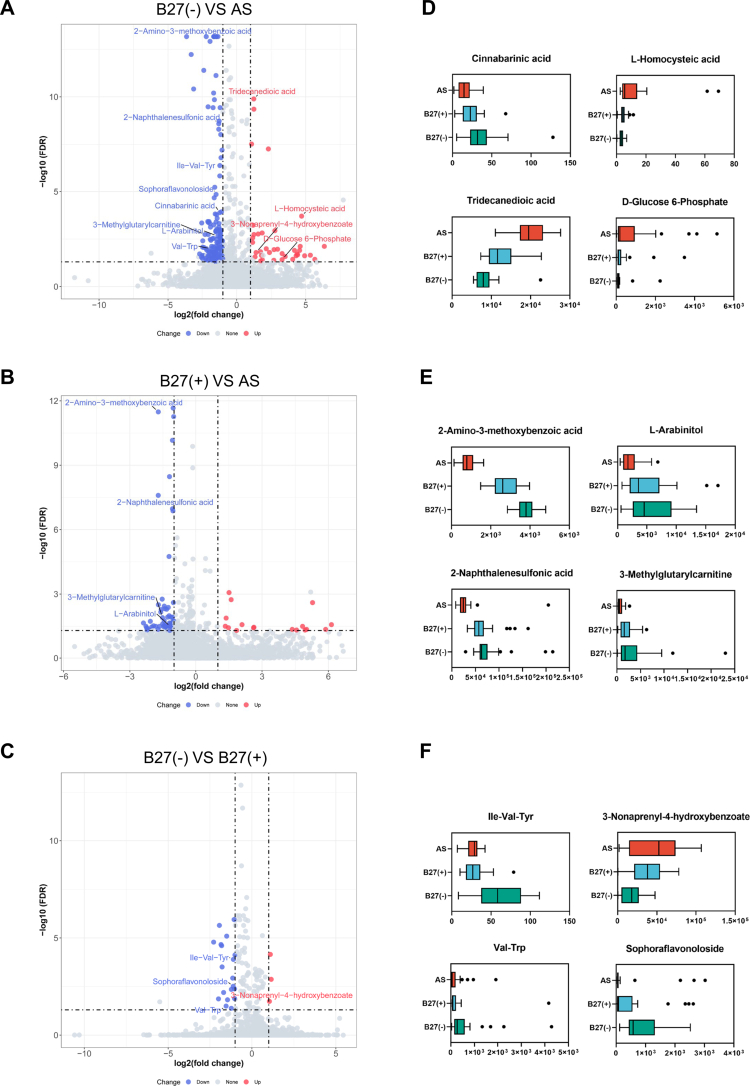
Significantly altered metabolites among AS, B27(+) and B27(−) groups. (A–C): Volcano plots demonstrated metabolites changes in B27(−) vs AS, B27(+) vs AS and B27(−) vs B27(+), respectively. The X axis indicates the log2-transformed fold change of metabolite abundances, and the Y axis indicates the negative value of the log10-transformed FDR using Kruskal-Wallis rank-sum test. The horizontal lines represent FDR < 0.05 and the vertical lines indicate fold change of >2 or <0.5. Metabolites that were elevated or decreased are highlighted in red and blue, respectively. (D): Boxplot showing representative metabolites that were significantly changed in the AS group compared with the B27(−) group. (E): Boxplot showing representative metabolites that were significantly changed in the AS group compared with both HC groups. (F): Boxplot showing representative metabolites that simultaneously differed in AS and B27(+) compared with B27(−). Boxes represent the 25th–75th quartile range, and lines within boxes denote median values. Whisker denotes the highest and lowest values within 1.5 times the IQR, and outliers are represented as dots.

Given that fecal metabolites reflect microbiome function, we performed untargeted LC–MS/MS profiling. OPLS-DA demonstrated clear separation of metabolic profiles among AS, B27(+), and B27(–) groups (Figure S3). Across the 7893 detected metabolites, differential analyzes (VIP > 1, FDR < 0.05, fold change ≥ 2 or ≤ 0.5) identified 196 features between AS and B27(–), 80 between AS and B27(+), and 22 between B27(+) and B27(–). Among the 196 AS vs B27(–) differences, 159 metabolites were decreased, and 37 were increased in AS (Figure 3A). Illustratively, the kynurenine-pathway derivative cinnabarinic acid was reduced, whereas the levels of cysteine-derived L-homocysteic acid and the glycolytic intermediate D-glucose-6-phosphate were elevated. Tridecanioic acid showed a particularly strong increase (FDR = 1.29 × 10⁻¹⁰; fold change = 2.36) (Figure 3D; Table S4). Compared with B27(+), the AS group exhibited 63 downregulated and 17 upregulated metabolites (Figure 3B). Thirty-eight metabolites differed in AS relative to both control groups, including the kynurenine-related 2-amino-3-methoxybenzoic acid and the sugar alcohol L-arabinitol. Notably, 2-amino-3-methoxybenzoic acid showed the largest shifts in both comparisons (B27(–) vs AS: FDR = 6.79 × 10⁻¹⁴; fold change = 0.22; B27(+) vs AS: FDR = 3.26 × 10⁻¹²; fold change = 0.30) (Figure 3E; Tables S4, S5). Between B27(+) and B27(–), 19 metabolites were decreased and 3 were increased in B27(+) (Figure 3C). Most of these (17/22) overlapped with the AS vs B27(–) differences, including the oligopeptide Ile-Val-Tyr and 3-nonaprenyl-4-hydroxybenzoate (Figure 3F; Tables S4, S6).

Collectively, these data indicate a substantial HLA-B27–associated shift in the fecal metabolome. AS is characterized by perturbed carbohydrate metabolism – evidenced by altered D-glucose-6-phosphate and L-arabinitol – as well as dysregulated amino-acid–related pathways, reflected in changes to cinnabarinic acid, 2-amino-3-methoxybenzoic acid, L-homocysteic acid, and Ile-Val-Tyr.

### Alterations of microbial function and related metabolites in AS with HLA-B27 background

**Figure 4.**
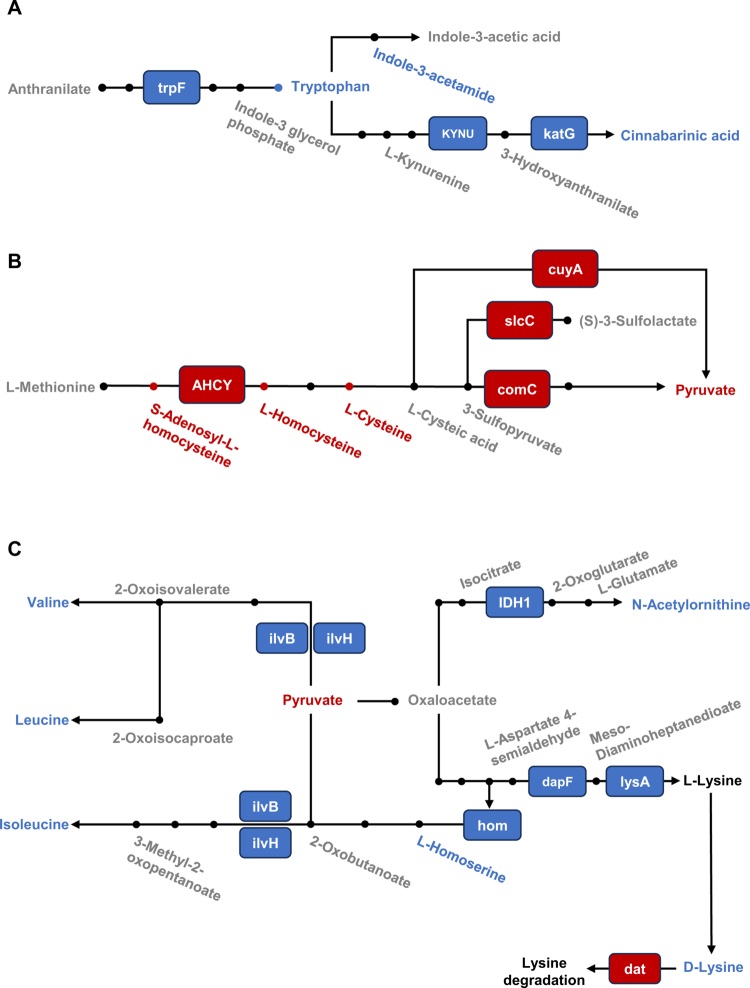
KEGG maps showing disturbed amino acid metabolic pathways in AS with HLA-B27 background. (A): Decreased levels of microbial genes (trpF, KYNU and katG) involved in tryptophan metabolism in the AS group relative to those in the B27(+) and B27(−) groups. Meanwhile, levels of tryptophan and its downstream metabolites (indole-3-acetamide and cinnabarinic acid) were also downregulated. (B): Increased levels of microbial genes (AHCY, cuyA, slcC and comC) involved in cysteine metabolism in AS group. Meanwhile, levels of homocysteine and its adjacent metabolites like S-adenosyl-L-homocysteine and cysteine were upregulated. Pyruvate, the catabolite of cysteine, was upregulated as well. (C): Decreased levels of microbial genes (ilvB, ilvH, hom, IDH1, dapF and lysA) involved in biosynthesis of branched-chain amino acid, ornithine and lysine in AS group, while the gene (dat) involved in lysine degradation was increased. In addition, levels of isoleucyl-valine, leucine, isoleucine, homoserine, *N*-Acetylornithine and lysine were downregulated. KEGG genes (squares) and metabolites are color-marked. Red indicates enriched microbial genes or metabolites in AS group, and blue indicates reduced in AS group. Metabolites marked in gray indicates no difference between the three groups or that no information was accessible. The pathways were generated on the basis of KEGG pathway maps.

Given that HLA-B27–associated shifts in community composition may alter microbial function – especially metabolism – we integrated KEGG-based functional profiles with fecal metabolomics. Across the AS, B27(+), and B27(–) groups, 258 KEGG Orthologs (KOs) differed. KEGG enrichment indicated the primary involvement of amino-acid metabolism and pentose–glucuronate interconversions (Figure S4), aligning with our metabolomic results. Mapping differential KOs to differential metabolites highlighted three perturbed pathways: “tryptophan metabolism” (Figure 4A), “cysteine metabolism” (Figure 4B), and “biosynthesis of branched-chain amino acids (BCAAs), ornithine, and lysine” (Figure 4C).

In the tryptophan metabolism, the AS group showed reduced abundance of trpF, suggesting diminished microbial tryptophan synthesis. The expression of catabolic genes (KYNU, katG) also decreased, which was concordant with the lower levels of the indole derivative indole-3-acetamide and the kynurenine-pathway derivative cinnabarinic acid (Figure 4A, Figure S5A–B). In the cysteine metabolism, AS exhibited upregulation of AHCY, implicated in homocysteine production, with corresponding increases in homocysteine, S-adenosyl-L-homocysteine, and cysteine. Cysteine-catabolic genes (cuyA, slcC, and comC) were also elevated, paralleling a rise in pyruvate (Figure 4B, Figure S5C–D). Despite increased pyruvate, the pyruvate-centered amino-acid biosynthetic axis was attenuated in AS. The expression of key BCAA biosynthetic genes (ilvB, ilvH) and hom genes was reduced, and metabolomics revealed lower levels of isoleucyl-valine, leucine, isoleucine, and homoserine. An ornithine-related gene (IDH1) was decreased, with reduced *N*-acetylornithine. For lysine, biosynthetic genes (dapF, lysA) were downregulated, whereas the degradative gene dat was upregulated, collectively yielding decreased lysine (Figure 4C, Figure S6).

Together, these integrated multi-omics data indicate that HLA-B27–linked functional reprogramming of the microbiome centers on amino-acid pathways, with coherent gene–metabolite shifts that are most pronounced in AS.

### Associations between disease-related microbiota and metabolites

To investigate how dysbiosis reshapes metabolic activity, we assessed species–metabolite relationships using Spearman rank correlations (Figure S7A).^19^ Within the three perturbed pathways (tryptophan, cysteine, and biosynthesis of branched-chain amino acids/ornithine/lysine), cinnabarinic acid (decreased in AS) correlated positively with the control-enriched *Lachnospiraceae bacterium 1_1_57FAA* (*ρ* = 0.22, *P* = 0.037) and negatively with the AS-enriched *Centipeda periodontii* (*ρ* = −0.301, *P* = 0.004). In contrast, S-adenosyl-L-homocysteine (increased in AS) correlated negatively with *Lachnospiraceae bacterium 1_1_57FAA* (*ρ* = −0.27, *P* = 0.011) and positively with *C. periodontii* (*ρ* = 0.21, *P* = 0.049). *N*-acetylornithine (decreased in AS) correlated positively with the control-enriched *Lachnospiraceae bacterium oral taxon 082* (*ρ* = 0.26, *P* = 0.014) and negatively with the AS-enriched *Bacteroides sp. An51A* (*ρ* = −0.22, *P* = 0.042).

Beyond these pathways, L-arabinitol (reduced in AS) was positively associated with *Lachnospiraceae bacterium 1_1_57FAA* (*ρ* = 0.42, *P* < 0.0001; Figure S7B). Tridecanedioic acid (elevated in AS) correlated positively with the AS-enriched *Negativibacillus massiliensis* (*ρ* = 0.31, *P* = 0.0036; Figure S7B). 2-amino-3-methoxybenzoic acid (markedly decreased in AS) correlated negatively with *C. periodontii* (*ρ* = −0.46, *P* < 0.0001; Figure S7B). Additional metabolites decreased in AS, including chivosazole B and sedanolide, showed negative correlations with AS-enriched taxa *Geosporobacter ferrireducens* and *Tannerella sp. CAG:118*, respectively (*ρ* = −0.54, *P* < 0.0001 and *ρ* = −0.45, *P* < 0.0001; Figure S7B).

Collectively, these correlations link disease-enriched species to reductions in control-enriched metabolites and highlight coherent species–metabolite pairs within amino-acid–related pathways.

### Associations of microbial taxa and metabolites with clinical parameters

**Figure 5.**
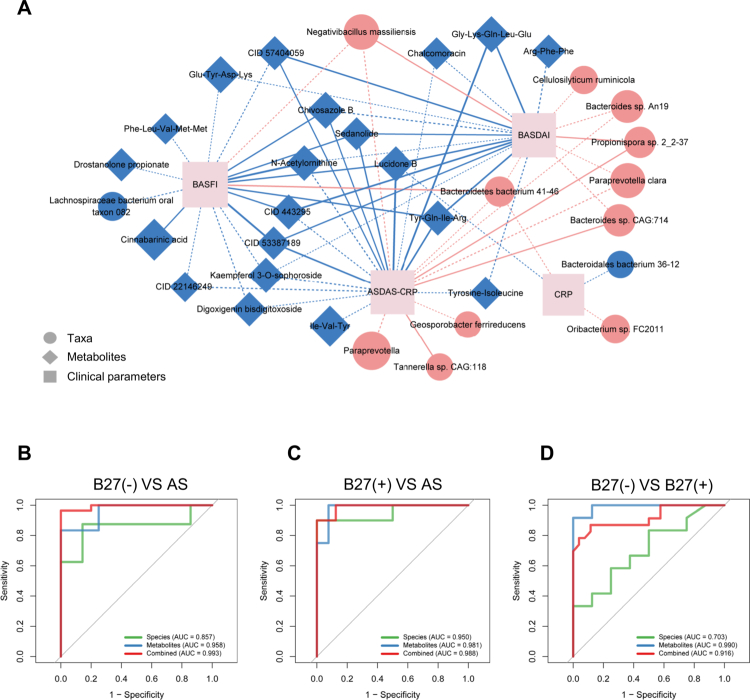
Integrative analysis of microbial and metabolic features with clinical parameters and random forest classification models. (A) Correlation network illustrating associations between differentially abundant microbial species or metabolites and clinical indices in the AS group. Circular and diamond nodes represent microbial and metabolic features that were increased (red) or decreased (blue) in the AS group compared to the B27(+) and B27(−) groups, respectively. The node size corresponds to the abundance level of the features. Edges between nodes indicate correlations, with red and blue representing positive and negative correlations, respectively. The edge thickness corresponds to the magnitude of the Spearman correlation coefficient. The solid edges denote statistically significant correlations, while dashed edges indicate suggestive correlations. (B–D) Performance of random forest classifiers built using species, metabolites, or their combination for discriminating between (B) B27(−) and AS, (C) B27(+) and AS, and (D) B27(−) and B27(+) groups. AUC, area under the curve.

To assess how disease-related taxa and metabolites are related to disease severity, we correlated differentially abundant features with clinical indices (BASDAI, ASDAS-CRP, BASFI, and CRP) using Spearman rank tests (Figure 5A). Among the taxa, *Bacteroidetes bacterium 41–46* correlated positively with all four parameters, reaching significance for BASFI (*ρ* = 0.45, *P* = 0.020). *Negativibacillus massiliensis* was positively correlated with three indices (all except CRP), with a significant association for BASDAI (*ρ* = 0.39, *P* = 0.048). In contrast, the AS-depleted *Lachnospiraceae bacterium oral taxon 082* showed a negative (trend-level) correlation with BASFI (*ρ* = −0.33, *P* = 0.104) (Table S7). Among the metabolites, lucidone B (reduced in AS) correlated negatively with all four parameters, with significant associations for BASDAI (*ρ* = −0.47, *P* = 0.015), ASDAS-CRP (*ρ* = −0.59, *P* = 0.002), and BASFI (*ρ* = −0.44, *P* = 0.024). Sedanolide showed significant negative correlations with BASDAI (*ρ* = −0.40, *P* = 0.042), ASDAS-CRP (*ρ* = −0.45, *P* = 0.023), and BASFI (*ρ* = −0.43, *P* = 0.028), but not CRP. Additional metabolites, including *N*-acetylornithine and chivosazole B, displayed similar inverse relationships. Notably, cinnabarinic acid – reduced in the AS tryptophan-metabolism pathway – was negatively associated with BASFI (*ρ* = −0.40, *P* = 0.044) (Figure 5A, Table S7).

Furthermore, Spearman correlation analysis was performed between clinical parameter-related microbiota and metabolites. Consistently, species previously identified as positively correlated with disease severity (e.g. *Bacteroidetes bacterium 41–46* and *Negativibacillus massiliensis*) were inversely correlated with putatively protective metabolites (e.g. lucidone B, sedanolide, and chivosazole B) in AS (Figure S8).

### Subgroup analysis of AS patients stratified by gastrointestinal symptoms

An exploratory subgroup analysis was performed between patients with (*n* = 8) and without (*n* = 20) gastrointestinal symptoms. Due to the limited sample size of the symptomatic subgroup, these comparisons are underpowered and should be interpreted as hypothesis-generating. Clinically, disease activity scores showed a trend toward being higher in patients with gastrointestinal symptoms (Figure S9A). At the species level, the abundance of *Bilophila wadsworthia* was significantly increased in the symptomatic subgroup, while other AS-enriched species such as *Bilophila sp. 4_1_30* and *Cloacibacillus sp. An23* showed nonsignificant increasing trends (Figure S9B). At the functional level (KO genes), the symptomatic subgroup showed a decreasing trend for trpF (in tryptophan metabolism) and a significant increase for cuyA (in cysteine metabolism), alongside significant alterations in ilvH and lysA (Figure S9C). Consistently, the levels of the metabolites L-Tryptophan, Indole-3-acetamide, and L-Isoleucine were significantly lower in symptomatic patients (Figure S9D). Notably, correlation analysis identified a significant association between *Lachnospiraceae bacterium oral taxon 082* and Sedanolide exclusively in the symptomatic subgroup, which was absent in asymptomatic patients (*ρ* = 0.71, *P* = 0.047) (Figure S9E). These preliminary findings require validation in larger, prospectively stratified cohorts.

### Random forest models based on disease-related taxa and/or metabolites

To assess the discriminatory power of microbial and metabolic profiles, we trained ten-fold cross-validated random forest classifiers using fecal taxonomic and/or metabolite features (Table S8). Using species alone, the model distinguished AS from B27(–) with strong performance (AUC = 0.857; Figure 5B; Table S8). Metabolite features performed even better (AUC = 0.958), and the combined feature set achieved the highest accuracy (AUC = 0.993; Figure 5B; Table S8). Similar results were obtained for AS versus B27(+): species AUC = 0.950, metabolites AUC = 0.981, combined AUC = 0.988 (Figure 5C; Table S8). In contrast, distinguishing B27(+) from B27(–) was more challenging for species alone (AUC = 0.703), whereas metabolites retained high performance (AUC = 0.990), and the combined model remained strong (AUC = 0.916) (Figure 5D; Table S8). Overall, models that integrate microbiota with fecal metabolites outperform those using either feature set alone for differentiating AS from HLA-B27–defined subgroups.

### AS patient-derived microbiota promotes M1 macrophage-mediated intestinal inflammation

**Figure 6.**
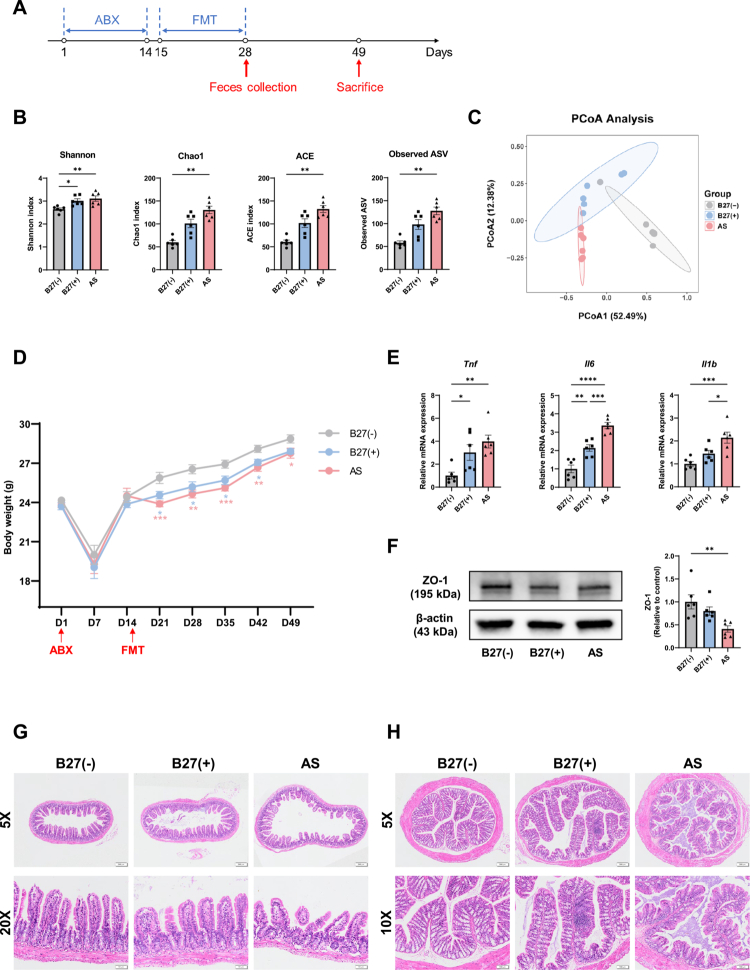
AS patient-derived microbiota promotes intestinal inflammation. (A) Schedule of establishment of FMT model and sample collection. (B) Alpha diversity measured by Shannon, Chao1, ACE and Observed ASV index. (C) PCoA among the B27(−), B27(+) and AS groups (Bray‒Curtis distance, ANOSIM, B27(−) vs AS, *P* = 0.002; B27(+) vs AS, *P* = 0.003; B27(−) vs B27(+), *P* = 0.012; B27(−) vs B27(+) vs AS, *P* = 0.001). (D) Body weight change curves of the three groups. (E) RT‒qPCR analysis of inflammatory factors in small intestinal tissue. (F) Western blot analysis of ZO-1 in small intestinal tissue. From left to right: representative immunoblot bands and corresponding quantitative analysis of ZO-1 protein expression. *β*-actin was used as a loading control. (G) Representative HE staining images of small intestinal tissue. (H) Representative HE-stained images of colon tissue. The data represent the mean ± SEM (*n* = 6). *P < 0.05, **P < 0.01, ***P < 0.001, **** *P* < 0.0001. ABX, antibiotics; FMT, fecal microbiota transplantation. PCoA, principal coordinate analysis.

To probe how HLA-B27–associated dysbiosis disrupts intestinal homeostasis in AS, we established an FMT model by colonizing germ-free mice via oral gavage with stool from AS, B27(+), or B27(–) donors, followed by systemic assessments of inflammation and bone metabolism (timeline in Figure 6A). After 14 d of daily gavage (day 28), fecal 16S rRNA sequencing confirmed engraftment. Compared with B27(–), AS-colonized mice showed higher alpha diversity by Shannon, Chao1, ACE, and observed ASVs; the Shannon index was also elevated in B27(+) (Figure 6B). Bray–Curtis PCoA demonstrated significant compositional differences among the groups, with B27(+) exhibiting an intermediate profile between B27(–) and AS (Figure 6C). The top 10 taxa at the phylum, genus, and species levels are shown in Figure S10A–C. At the phylum level, AS mice presented decreased *Firmicutes* and increased *Bacteroidota*, yielding a lower *Firmicutes*/*Bacteroidota* ratio versus B27(–). Overall, post-FMT community profiles mirrored those from donor metagenomes, indicating successful transplantation.

**Figure 7.**
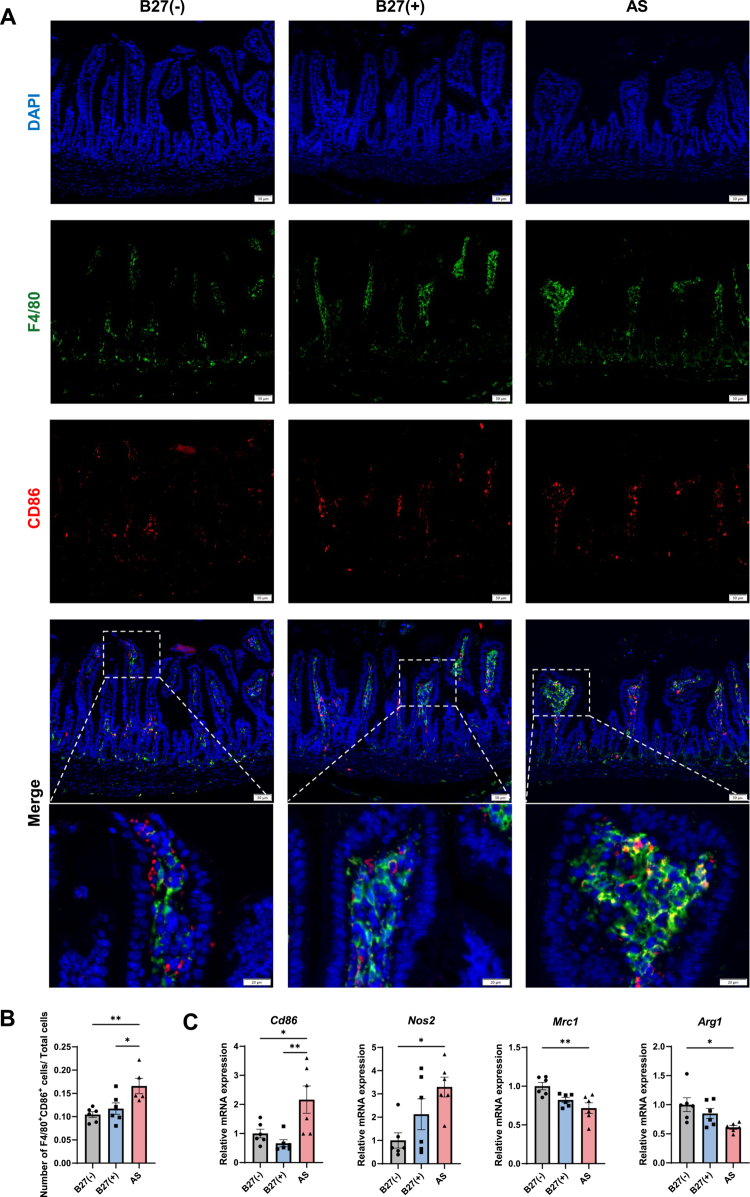
M1 macrophages mediate intestinal inflammation induced by AS patient-derived microbiota. (A) Representative immunofluorescence staining of F4/80 and CD86 in small intestine tissue. (B) Quantification of F4/80⁺CD86⁺ double-positive cells per field of view. (C) RT‒qPCR analysis of M1-related markers (*Cd86* and *Nos2*) and M2-related markers (*Mrc1* and *Arg1*) in small intestinal tissue. The data represent the mean ± SEM (*n* = 5‒6). *P < 0.05, **P < 0.01.

Body weight trajectories diverged after transplantation: both the AS and B27(+) groups weighed less than the B27(–) group throughout the observation period (Figure 6D). In the small intestine, RT–qPCR revealed higher *Tnf*, *Il6*, and *Il1b* expression in AS than in B27(–); *Tnf* and *Il6* were also elevated in B27(+) (Figure 6E). The tight junction marker ZO-1 was reduced in AS intestinal tissue (Figure 6F; Figure S10D). Histology showed shorter villi in both B27(+) and AS versus B27(–), with the AS group displaying disorganized villous architecture (Figure 6G). In colon, gland numbers were reduced in B27(+) and AS, and the AS group exhibited mild lamina propria inflammatory infiltrates (Figure 6H). Macrophage infiltration and polarization were then assessed in small intestine. F4/80 immunohistochemistry localized predominantly to lamina propria and submucosa, with stronger staining in AS versus B27(–), indicating increased macrophage infiltration (Figure S10E–F). Double immunofluorescence showed more F4/80⁺CD86⁺ cells in AS, consistent with M1-skewing (Figure 7A-B). Similarly, RT–qPCR demonstrated the upregulation of M1 markers (*Cd86, Nos2*) and the downregulation of M2 markers (*Mrc1, Arg1*) in AS (Figure 7C).

Together, these data show that microbiota from HLA-B27–positive donors compromise intestinal barrier integrity and elicit localized inflammation in recipient mice, with M1-polarized macrophages as key effectors.

### AS patient-derived microbiota promotes systemic inflammation and bone loss

Flow cytometry of splenic T-cell subsets showed that, versus B27(–), the AS group had fewer Tregs and higher proportions of Th17 and Th1 cells. The B27(+) group also displayed reduced Tregs and increased Th17 cells, with no significant change in Th1 (Figure S11). Monocytes and macrophages were profiled across peripheral tissues. In spleen, both populations were elevated in AS versus the other groups. In peripheral blood, AS and B27(+) exceeded B27(–), whereas bone marrow showed no group differences (Figure S12). Macrophage polarization shifted toward an M1 phenotype: AS mice exhibited more M1 and fewer M2 macrophages in spleen and blood compared with B27(–) (Figure S13A–B). Serum cytokines (CBA) revealed higher TNF in AS and B27(+) versus B27(–); IL-6 and IFN-*γ* trended upward in AS (Figure S13C). Similarly, bone marrow cells from AS mice showed increased *Tnf* expression by RT–qPCR (Figure S13D).

**Figure 8.**
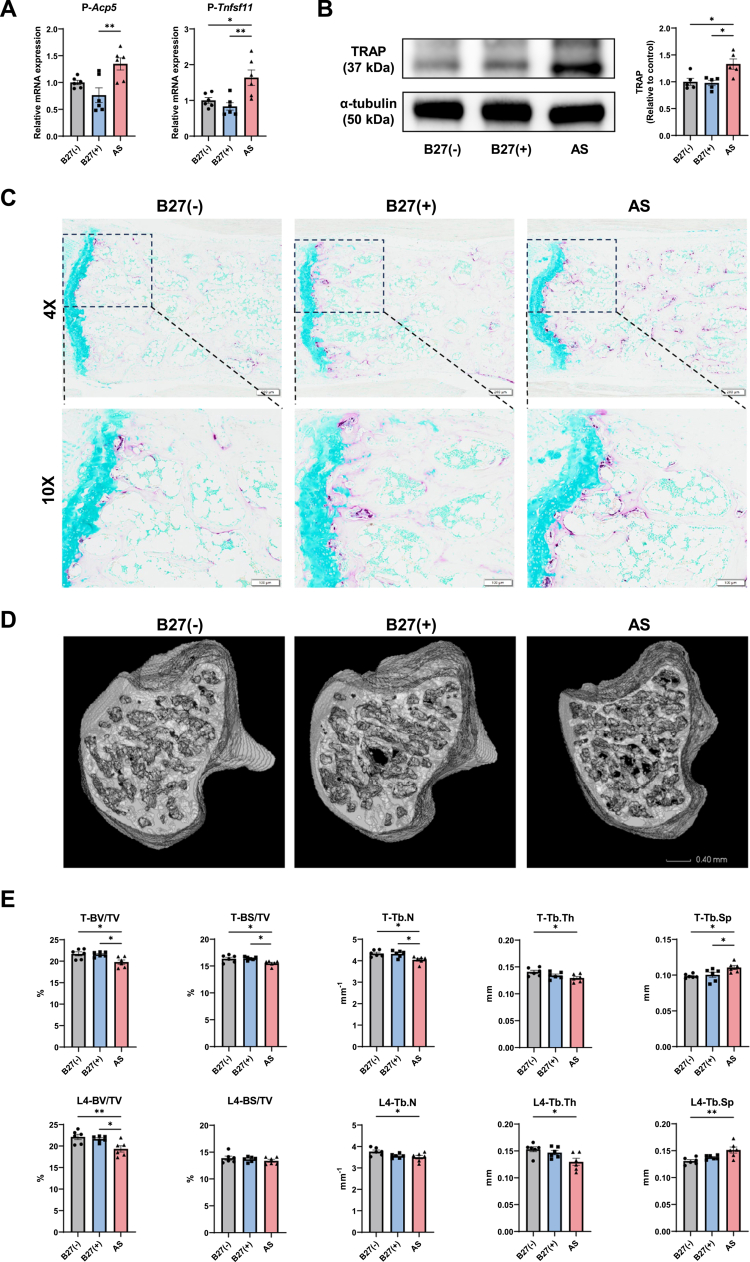
AS patient-derived microbiota promotes bone loss. (A) RT‒qPCR analysis of osteoclastogenesis-related genes (*Acp5* and *Tnfsf11*) in hind paw tissue. (B) Western blot analysis of TRAP in hind paw tissue. From left to right: representative immunoblot bands and corresponding quantitative analysis of TRAP protein expression. *α*-tubulin was used as a loading control. (C) Representative TRAP staining images of caudal vertebrae. (D) Representative 3D micro-CT images of the tibia. (E) Quantitative analysis of bone mass and trabecular parameters in the tibia and fourth lumbar vertebra. The data represent the mean ± SEM (*n* = 5–6). *P < 0.05, **P < 0.01. *P* indicates the hind paw; T indicates the tibia; L4 indicates the fourth lumbar vertebra.

Markers of osteoclastogenesis were upregulated in AS: *Acp5* and *Tnfsf11* were elevated in the bone marrow and hind paw (Figure 8A; Figure S13D), and tartrate-resistant acid phosphatase (TRAP) was increased in the hind paw, as shown by Western blot (Figure 8B). TRAP staining of the caudal vertebrae demonstrated more TRAP-positive cells in bone resorption sites – subchondral bone at endplates and along trabeculae – in AS versus B27(–) (Figure 8C). Micro-CT of tibia and L4 revealed lower bone volume fraction (BV/TV) in AS than in B27(–) and B27(+), along with reduced bone surface/total volume (BS/TV) in tibia, indicating diminished bone mass. Trabecular metrics in AS further showed decreased number (Tb.N) and thickness (Tb.Th) and increased spacing (Tb.Sp) relative to B27(–) (Figure 8D-E).

Collectively, FMT with the AS donor microbiota provoked systemic inflammation, M1-skewed immunity, and an osteocatabolic state culminating in trabecular bone loss.

### CA inhibits M1 macrophage polarization via AhR signaling

**Figure 9.**
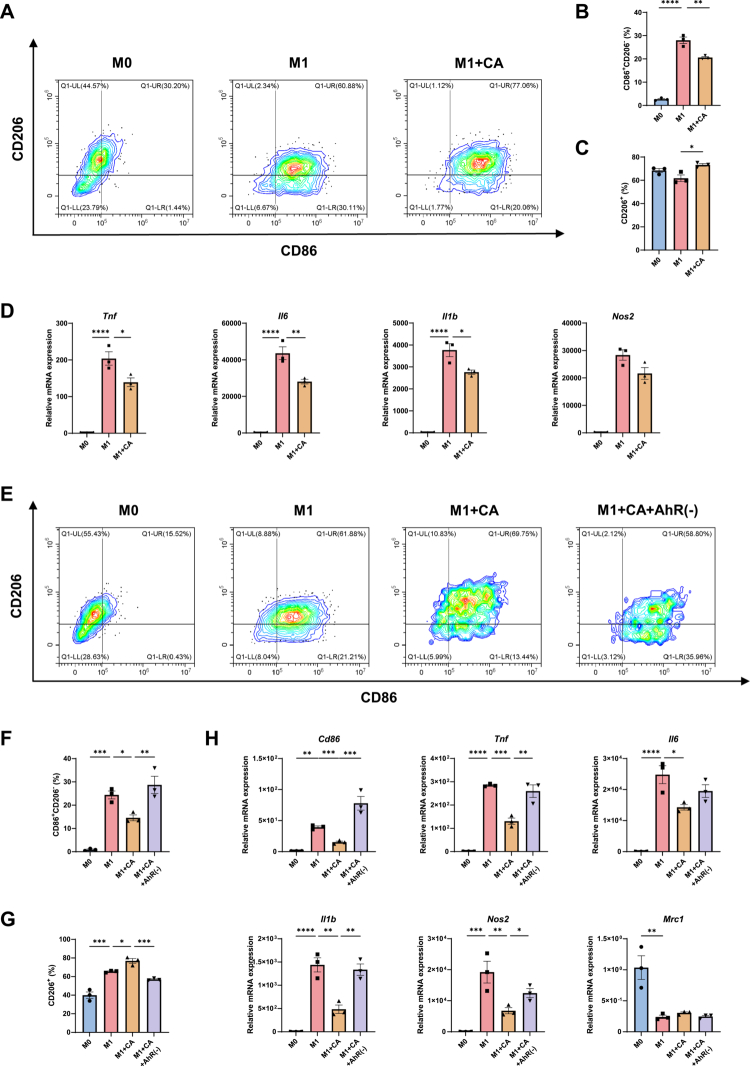
CA inhibits M1 macrophage polarization via AhR. A–D: Effects of CA treatment on macrophage polarization. (A) Representative flow cytometry plots of CD86 and CD206 expression. (B) Proportion of CD86⁺CD206⁻ cells across groups. (C) Proportion of CD206⁺ cells across groups. (D) RT–qPCR analysis of inflammation cytokines. E–H: Effects of CA in the presence of CH-223191. (E) Representative flow cytometry plots of CD86 and CD206 expression. (F) Proportion of CD86⁺CD206⁻ cells. (G) Proportion of CD206⁺ cells. (H) RT‑qPCR analysis of marker genes and inflammation cytokines. Data are presented as mean ± SEM (*n* = 3). *P < 0.05, **P < 0.01, ***P < 0.001, ****P < 0.0001.

In our multiomics analysis, the microbial-derived metabolite cinnabarinic acid (CA) was significantly reduced in the AS group and showed strong correlations with multiple AS-associated bacterial species and clinical indicators. Based on these findings, CA was selected for mechanistic validation. To induce classical M1 polarization, BMDMs were treated with lipopolysaccharide (LPS) and interferon-*γ* (IFN-*γ*), with CA (100 µM) added to the culture medium. After 24 h, flow cytometric analysis demonstrated that CA significantly reduced the proportion of CD86⁺CD206⁻ M1 macrophages (Figure 9A and B) while increasing the proportion of CD206⁺ cells (Figure 9A and C). Consistent with these phenotypic changes, CA markedly downregulated the mRNA expression of the proinflammatory cytokines *Tnf*, *Il6*, and *Il1b*, with *Nos2* expression also showing a decreasing trend (Figure 9D).

To determine whether the anti-inflammatory effects of CA were mediated through aryl hydrocarbon receptor (AhR) signaling, the selective AhR antagonist CH-223191 was employed. BMDMs were pretreated with CH-223191 for 1 h prior to M1 polarization and CA exposure. Compared with CA treatment alone, AhR antagonism significantly increased the proportion of CD86⁺CD206⁻ cells and reduced the CD206⁺ population (Figure 9E–G). At the transcriptional level, blockade of AhR signaling reversed the effects of CA, as evidenced by increased expression of the M1 marker *Cd86* and the inflammatory mediators *Tnf*, *Il1b*, and *Nos2*, accompanied by reduced expression of the M2 marker *Mrc1* (Figure 9H). Consistent with these findings, Western blot analysis showed that CA suppressed inflammation-induced phosphorylation of IKKα/β, IκBα, and p65. This inhibitory effect was abolished by AhR antagonism, as indicated by restored phosphorylation of IκBα and p65 (Figure S14).

### CA mediates protective effects in the FMT model

**Figure 10.**
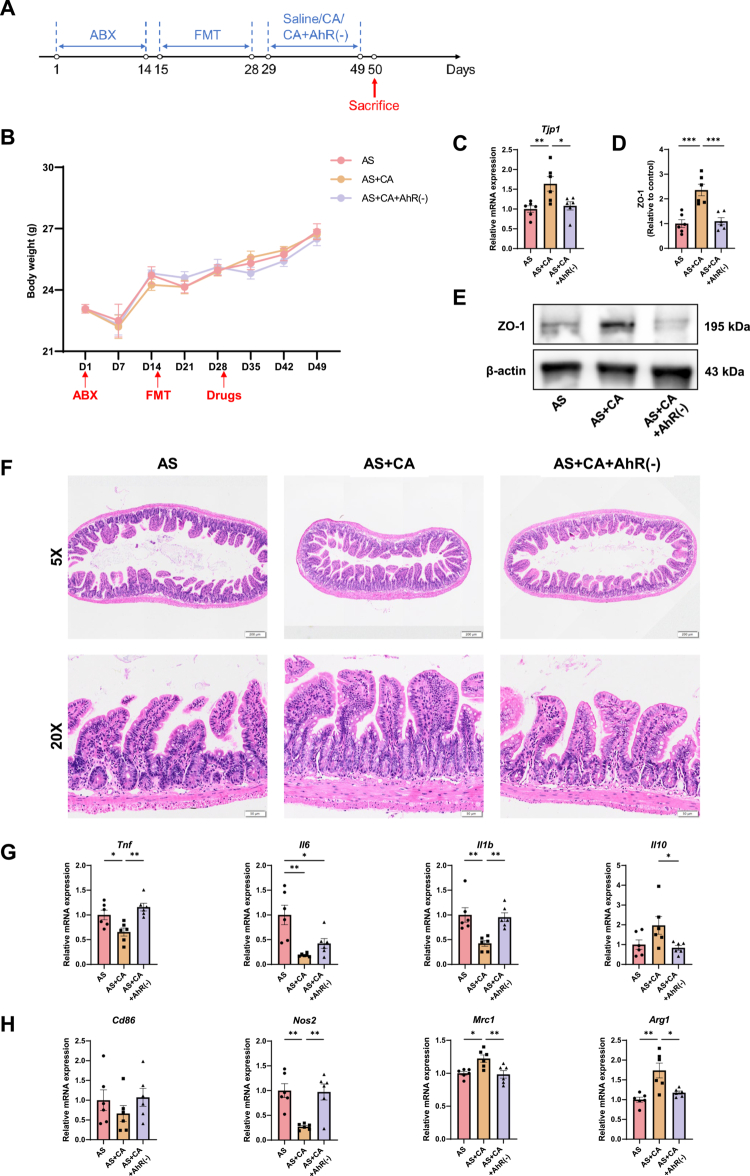
Protective effects of CA assessed in the FMT model. (A) Schedule of treatment and sample collection. (B) Body weight change curves of the AS, AS + CA and AS + CA + AhR(-) groups. (C) RT–qPCR analysis of *Tjp1* in small intestinal tissue. D-E: Western blot analysis of ZO-1 in small intestinal tissue. Quantitative analysis of ZO-1 protein expression (D) and representative immunoblot bands (E). *β*-actin was used as a loading control. (F) Representative HE staining images of small intestinal tissue. (G) RT‒qPCR analysis of inflammatory factors in small intestinal tissue. (H) RT‒qPCR analysis of M1‑related markers in small intestinal tissue. The data are presented as mean ± SEM (*n* = 6). *P < 0.05, **P < 0.01, ***P < 0.001.

To evaluate the therapeutic potential of CA *in vivo*, CA was administered to AS-FMT mice, with concurrent treatment using the AhR antagonist CH-223191 to assess AhR-dependent effects (Figure 10A). Body weight was monitored throughout the experimental period, and no significant differences were observed among the treatment groups (Figure 10B). RT–qPCR and Western blot analyzes demonstrated that CA treatment markedly increased both mRNA and protein expression of the tight junction protein ZO-1 compared with AS-FMT mice. This enhancement of barrier-associated protein expression was substantially attenuated by pharmacological blockade of AhR signaling (Figure 10C–E). Consistent with these molecular findings, histological analysis revealed that CA improved intestinal morphology, characterized by more regular villous architecture and increased villus length. In contrast, AhR inhibition largely reversed these effects, resulting in villus morphology comparable to that observed in untreated AS-FMT mice (Figure 10F).

At the inflammatory level, CA treatment significantly reduced the expression of proinflammatory cytokines (*Tnf*, *Il6*, and *Il1b*) in the small intestine, while co-administration of the AhR antagonist restored their expression to levels similar to those in AS-FMT mice. In parallel, CA increased the expression of the anti-inflammatory cytokine *Il10*, an effect that was abolished by AhR inhibition (Figure 10G). Analysis of macrophage polarization further showed that CA suppressed M1-associated genes (*Nos2* and *Cd86*) and upregulated M2 markers (*Mrc1* and *Arg1*), whereas these regulatory effects were reversed upon AhR antagonism (Figure 10H). Consistent with the transcriptional data, dual immunofluorescence staining revealed a marked reduction in F4/80⁺CD86⁺ macrophages in the intestinal mucosa of CA-treated mice, indicating decreased infiltration of pro-inflammatory M1 macrophages. This effect was again negated by blockade of AhR signaling (Figure S15).

In addition to intestinal effects, TRAP staining of the caudal vertebrae demonstrated a significant reduction in osteoclast activity in CA-treated mice, as evidenced by fewer TRAP-positive cells along subchondral endplates and trabecular surfaces compared with AS-FMT controls. This osteoprotective effect was largely abrogated by AhR inhibition (Figure S16A). Consistently, micro-CT analysis of the L4 vertebra and tibia showed that CA administration significantly increased BV/TV and BS/TV in the L4 vertebra, with similar upward trends observed in the tibia. Improvements in trabecular microarchitecture – including increased Tb.N and Tb.Th and reduced Tb.Sp – were also observed relative to the AS-FMT group. Notably, these beneficial skeletal effects were eliminated following pharmacological blockade of AhR signaling (Figure S16B–C).

Collectively, these findings demonstrate that CA improves intestinal barrier integrity, suppresses gut inflammation, and preserves bone mass in the AS-FMT model, with its protective effects largely mediated through AhR-dependent signaling pathways.

## Discussion

In this study, we integrated gut microbiome profiles, fecal metabolomics, and clinical phenotyping across HLA-B27–positive AS patients and HLA-B27–positive/negative healthy controls. We observed marked disruptions in both microbial composition and metabolite profiles among HLA-B27–positive participants, with the most pronounced alterations in AS. Notably, amino-acid–related pathways emerged as a defining feature of the AS gut ecosystem. Correlation networks linking differential species and metabolites to disease indices (e.g. BASDAI, ASDAS-CRP, BASFI) highlighted candidate microbial–metabolic factors associated with severity. To probe causality at the community level, we established a human-to-mouse FMT model and found that AS-derived microbiota impaired intestinal barrier integrity, promoted systemic inflammation, and induced bone loss, accompanied by M1-skewed macrophage responses. Mechanistic validation further showed that the microbial-derived metabolite CA suppresses M1 macrophage polarization via AhR signaling and confers protection in the FMT model. Collectively, these findings support a model in which HLA-B27 is associated with a dysbiotic and metabolically reprogrammed gut ecosystem that contributes to AS pathogenesis through macrophage-mediated inflammation and osteocatabolic signaling.

Numerous studies have linked AS to gut microbiome dysbiosis, yet the mechanisms by which altered communities shape host immunity remain incompletely defined. The contribution of HLA-B27 – a class I MHC allele – to AS risk is well established, and bacterial peptides mimicking HLA-B27 epitopes have been reported in AS cases.^9^ However, whether and how HLA-B27 influences gut microbial composition and metabolism in humans remains unclear. In our cohort, AS, B27(+), and B27(–) groups exhibited distinct community structures, with B27(+) showing an intermediate profile. Differences were detectable across phylum, genus, and species levels. These patterns are consistent with prior work reporting microbiome differences between HLA-B27–positive and –negative healthy individuals^24^ and support the concept that HLA-B27 can shape the gut ecosystem. At the same time, the divergence between HLA-B27–positive and –negative healthy controls in our cohort was modest, whereas microbiome–metabolome alterations were most prominent in HLA-B27–positive individuals with AS. This suggests that HLA-B27–associated effects are context dependent rather than deterministically pathogenic.

Across the three groups, we identified 40 species with stepwise changes in abundance. Among depleted taxa, *Lachnospiraceae bacterium oral taxon 082* and *Lachnospiraceae bacterium 1_1_57FAA –* members of the SCFA-producing Lachnospiraceae family – were reduced, consistent with reports in Crohn's disease and rheumatoid arthritis (RA).^25^ In contrast, taxa enriched in both AS and B27(+) were dominated by *Bacteroides*, a genus that includes opportunistic pathogens. *Paraprevotella clara* was also increased in AS and B27(+), and has been linked to autoimmune disorders such as primary biliary cirrhosis and granulomatous panuveitis;^26^^,^^27^ notably, the latter study connected disease susceptibility at HLA-DRA with *Paraprevotella clara*.^27^ Together with our HLA-B27–stratified findings, these observations raise the possibility that *Paraprevotella spp*. intersect with HLA-related immune pathways. Consistent with prior work,^8^ *Bilophila wadsworthia* was enriched in AS compared with B27(–); this organism can compromise epithelial barrier integrity and promote inflammation, with potential effects on bile-acid and glucose homeostasis.^28^^,^^29^ We also identify previously unreported AS-associated taxa, including *Negativibacillus massiliensis* and *Centipeda periodontii* (the latter implicated in periodontitis pathogenesis),^30^ expanding the list of candidate microbes for mechanistic investigation in AS development and progression.

At the functional level, OPLS-DA showed clear separation of fecal metabolomes among groups. Differential analyzes indicated that metabolic perturbations in HLA-B27–positive participants were concentrated in carbohydrate and amino-acid pathways. In AS, reduced L-arabinitol together with elevated D-glucose-6-phosphate suggest altered carbohydrate utilization and metabolic flux, which is consistent with an inflammatory metabolic state. Concordantly, untargeted metabolomics and KEGG-based functional inference converge on amino-acid metabolic disruption as a defining feature of AS.

KEGG pathway mapping highlighted three amino-acid–related pathways disrupted in AS: tryptophan metabolism, cysteine metabolism, and biosynthesis of branched-chain amino acids, ornithine, and lysine. Within tryptophan metabolism, both biosynthetic and catabolic nodes were reduced in AS. Cinnabarinic acid (CA) – a kynurenine-pathway derivative – was decreased and has been reported to exert protective effects in experimental autoimmune encephalomyelitis and liver injury, as well as analgesic activity in inflammatory settings.^31-33^ Another kynurenine-related metabolite, 2-amino-3-methoxybenzoic acid, was likewise reduced. Indole-3-acetamide, a precursor of indole-3-acetic acid (IAA), was also lower in AS; notably, in a proteoglycan-induced spondylitis model, IAA attenuated arthritis, suppressed pro-inflammatory cytokines, and restored the Th17/Treg balance,^34^ and it can exert anti-angiogenic effects *in vitro*.^35^ Because both CA and IAA signal through the AhR, diminished availability of AhR ligands may contribute to mucosal and systemic inflammation in AS and nominates AhR-centered pathways as testable therapeutic targets.

Within cysteine metabolism, AS showed increased homocysteine synthesis accompanied by elevated downstream metabolites (cysteine and pyruvate). Elevated homocysteine is a recognized risk factor for cardiovascular and cerebrovascular disease and can amplify immunoinflammatory responses by activating inflammatory monocytes, Th1/Th17 cells, and neutrophils via NF-κB and JAK2/STAT3 signaling.^36^^,^^37^ In contrast, increased pyruvate has been reported to exert antioxidant and anti-inflammatory effects,^38^ potentially reflecting a counterregulatory response in an inflamed gut environment.

Conversely, the pyruvate-centered amino-acid biosynthetic axis was attenuated in AS, with reduced levels of branched-chain amino acids (valine, leucine, isoleucine), lysine, and *N*-acetylornithine. *In vivo*, BCAA supplementation has been reported to partially restore dysbiotic gut communities and suppress inflammatory signaling.^39^ Lysine supports protein synthesis, energy metabolism, mineral absorption, bone formation, and immune function,^40^ with bone and immune regulation being particularly relevant to long-term AS care. Ornithine, a downstream product of *N*-acetylornithine, has been proposed as a nutraceutical to mitigate immune dysfunctions linked to LACC1, including arthritis and inflammatory bowel disease.^41^ Consistent with these observations, *N*-acetylornithine was inversely associated with BASFI in our cohort. Although interventional trials are required, these data raise the possibility that correcting specific amino-acid deficiencies may serve as an adjunctive strategy in AS.

Correlation analyzes further highlighted taxa and metabolites potentially linked to disease severity. *Negativibacillus massiliensis* and *Bacteroidetes bacterium 41–46* correlated positively with the BASDAI and BASFI, respectively. Among the metabolites, CA showed a significant inverse association with the BASFI, suggesting a potential protective role in AS pathophysiology. Several metabolites – including sedanolide, chivosazole B, and lucidone B – were negatively associated with activity and function indices and were also correlated with AS-enriched taxa. Prior studies describe anti-inflammatory or cytoprotective properties of these compounds,^42‐44^ but targeted validation will be required to establish their mechanistic relevance in AS.

The humanized FMT model reconstructs gut microbial communities in antibiotic-treated mice using microbiota from human donors. Following broad-spectrum antibiotic depletion, donor communities can colonize stably and partially recapitulate donor composition, enabling controlled interrogation of host–microbiome immune interactions. This approach has provided insights across conditions including depression, inflammatory bowel disease, osteoarthritis, and RA.^23,45‐47^ To test whether AS-associated microbiota contribute causally to inflammation, we performed FMT using clinical donor stool. 16S rRNA sequencing confirmed engraftment, and recipient community structures – including between-group differences – closely mirrored the human metagenomic profiles. AS recipients displayed a reduced *Firmicutes*/*Bacteroidota* (F/B) ratio, consistent with inflammatory dysbiosis.^48^ Although antibiotics can influence immunity and bone metabolism,^49^^,^^50^ all groups underwent the same depletion and recovery protocol, thereby minimizing confounding. Thus, this model provides a tractable platform to evaluate AS-associated microbial effects at the community level.

In the FMT model, AS-derived microbiota impaired intestinal barrier integrity and promoted systemic inflammation, immune dysregulation, and bone loss. Microbiota from HLA-B27–positive donors (B27(+) and AS) reduced splenic Tregs and increased Th17 cells, with the strongest effects in AS recipients, consistent with findings in HLA-B27 transgenic rats.^51^^,^^52^ Unlike AS recipients, B27(+)–colonized mice did not show significant increases in Th1 cells or macrophage proportions. In contrast, AS recipients exhibited pronounced M1 macrophage infiltration in the intestinal mucosa, elevated M1 polarization in spleen and blood, and increased TNF-*α* expression across intestinal, systemic, and bone marrow compartments. The enhanced osteoclastogenesis in bone and paw tissues resulted in significant bone loss in AS recipients, a phenotype consistent with other mouse model of AS.^53^ Together, these findings suggest that while HLA-B27–associated dysbiosis can shift adaptive immunity toward a Treg/Th17 imbalance, a key pathway driving inflammation and osteocatabolic phenotypes in this model is excessive M1 macrophage activation and heightened osteoclastogenesis under inflammatory conditions triggered by AS-associated microbiota. Importantly, supplementation with CA mitigated intestinal inflammation and bone loss in AS-FMT mice, and these protective effects were attenuated by AhR antagonism, supporting an AhR-dependent mechanism. Our signaling data further suggest that CA limits inflammation at least in part through AhR-dependent suppression of NF-κB activation, thereby restraining M1 macrophage polarization.

This study has several limitations. First, the modest, single-center cohort may limit generalizability. Second, although we attempted to control key confounders (e.g. age, sex, medications), unmeasured factors such as diet, lifestyle, and physiological variability may still influence microbial and metabolic profiles. Future prospective studies should incorporate standardized dietary and physical activity assessments together with relevant biomarker measurements. Third, while the FMT experiments support a community-level causal role of AS-associated microbiota, the pathogenicity of individual taxa (e.g. *Negativibacillus massiliensis* or *Bacteroidetes bacterium 41–46*) was not directly tested. Strain-level validation using monocolonization or defined consortia in germ-free mice was not feasible due to technical, ethical, and practical constraints. Accordingly, these taxa should be viewed as candidates rather than proven drivers, and future studies using targeted isolation and host–microbe interaction assays will be required to establish sufficiency. Finally, our cross-sectional design and sample size do not allow us to conclude that HLA-B27 directly regulates the microbiota or metabolome; therefore, we interpret these patterns as HLA-B27–associated rather than definitively HLA-B27–driven alterations.

In conclusion, our integrated microbiome–metabolome–clinical analyzes indicate that HLA-B27 is associated with a dysbiotic and metabolically reprogrammed gut ecosystem in AS. These alterations are subtle in healthy carriers but become pronounced in patients. Human-to-mouse FMT into antibiotic-treated recipients recapitulated intestinal barrier disruption and osteocatabolic phenotypes, supporting a community-level causal contribution of AS-derived microbiota. Furthermore, CA inhibited M1 macrophage polarization via AhR-dependent suppression of NF-κB signaling and exerted protective effects in the AS-FMT model. Going forward, larger multi-center cohorts and deeper mechanistic dissection – including the use of cell-specific or systemic AhR-knockout models to establish causal signaling roles – will be needed to define strain-level and receptor-level drivers. Interventional studies, such as microbiome modulation (e.g. targeted consortia), metabolite supplementation or lowering (e.g. BCAA/lysine restoration, homocysteine reduction), and macrophage- or osteoclast-targeted therapies, should then test whether correcting these microbial–metabolic abnormalities can modify disease activity and bone outcomes.

## Supplementary Material

Supplemental materials.docx Supplemental materials.docx

## Data Availability

The metagenome sequencing data have been deposited to the NCBI Sequence Read Archive and are available at the accession number PRJNA1004858 (reviewer link: https://dataview.ncbi.nlm.nih.gov/object/PRJNA1004858?reviewer=67cda5ken7ssdhkailojjucvcv).
