# Supplementary material for: HLA-B27-associated gut microbiota and amino acid perturbations promote ankylosing spondylitis through M1 macrophage activation
Source: Gut Microbes. 2026 Feb 16;18(1):2630561. doi: 10.1080/19490976.2026.2630561 (PMC12915779; doi:10.1080/19490976.2026.2630561)
Supplement: Supplemental materials.docx [file KGMI_A_2630561_SM5077.docx]

**Supplemental information**

**HLA-B27-Associated Gut Microbiota and Amino Acid Perturbations Promote Ankylosing Spondylitis through M1 Macrophage Activation**

Tianwen Huang^1,2^, Hang Yang^1^, Lingshu Zhang^1^, Xiangpeng Wang^1^, Ye Chen^1^, Huanzi Dai^2^, Kenji Hashimoto^3^, Yubin Luo^1*^, Yaoyu Pu^1*^ and Yi Liu^1,4*^

**Affiliations:** ^1^Department of Rheumatology and Immunology, West China Hospital, Sichuan University, Chengdu, China, ^2^Department of Rheumatology and Immunology, Daping Hospital, Army Military Medical University, Chongqing, China, ^3^Chiba University Center for Forensic Mental Health, Chiba, Japan, and ^4^West China Lecheng Hospital, Sichuan University, Boao, Hainan, China.

***Correspondence:** Prof. Yubin Luo (luoyubin2016@163.com), Dr. Yaoyu Pu ([puyaoyu@scu.edu.cn](mailto:puyaoyu@scu.edu.cn)) and Prof. Yi Liu ([yiliu8999@wchscu.cn](mailto:yiliu8999@wchscu.cn))

ORCID:

https://orcid.org/0000-0002-7669-1579 (Yubin Luo)

https://orcid.org/0000-0002-7887-3237 (Yaoyu Pu).

https://orcid.org/0000-0002-9258-3594 (Yi Liu).

Supplementary methods

Supplemental figures: 16

Supplemental tables: 8

**Supplementary methods**

**Hematoxylin-Eosin (HE) staining**

The sections of small intestine and colon were dewaxed and hydrated, rinsed in water, and then stained with hematoxylin for 5 minutes, followed by a 10- minute rinse. The sections were fixed with a bluing reagent, rinsed again, and stained with eosin for 1-2 minutes. Finally, the sections were dehydrated through a series of ethanol washes, cleared in xylene, and mounted.

**Immunohistochemistry**

Following high-temperature and high-pressure antigenic retrieval (EDTA, pH 9.0, 95℃, 30 min), the prepared tissue sections were blocked with 3% H2O2 and washed with PBS. Next, the sections were blocked with 3% BSA for 30 min and then incubated overnight at 4°C with primary antibody targeting F4/80 (1:500, Servicebio, # GB12027). The following day, after triple washing with 0.05 M Tris–HCl saline (TBS) with 0.1 % Tween-20 (TBST), the sections were incubated with HRP-conjugated goat anti-rabbit IgG (1:200, ABclonal, # AS014) at 37°C for 45 min, followed by another round of triple washing with TBST. Subsequent to DAB staining (MXB^®^ Biotechnologies, #DAB4033) and hematoxylin staining, the slides were washed with PBS. Dehydration and clearing of the sections were accomplished using absolute alcohol. Neutral gum was applied to cover the slices. Slides were imaged with a VS200 whole-slide scanner and analyzed using OlyVIA 4.1 software.

**Flow cytometry analysis**

Flow cytometry was conducted to evaluate immune cell populations in the spleen, bone marrow (BM), and blood of mice following fecal microbiota transplantation. Erythrocytes were removed with lysis buffer. Single-cell suspensions were filtered through a 70-μm strainer, washed with PBS, and resuspended in staining solution for antibody staining.

For T helper cell profiling, cells were stimulated with a cell stimulation cocktail (Tonbo Biosciences) for 6 hours prior to staining. The following fluorochrome-conjugated antibodies were used: APC-Cy7 anti-mouse CD3e, Percp-Cy5.5 anti-mouse CD4, FITC anti-CD25, Alexa Fluor 647 anti-Foxp3, APC anti-IL-4, FITC anti-IFN-γ, PE anti-IL-17A, PerCP-Cy5.5 anti-ly6G, Brilliant Violet 421 anti-F4/80, PE-Cy7 anti-Ly-6C, FITC anti-CD11b, BV510 anti-CD45, PE anti-CD86, and Alexa Fluor 647 anti-CD206. Fixable Viability Stain 700 was used to exclude dead cells. All antibodies were obtained from BD Biosciences.

Intracellular cytokine staining (IFN-γ, IL-4, IL-17A) was performed using the BD Intracellular Fixation & Permeabilization Buffer Set, while Foxp3 staining was carried out with the eBioscience Transcription Factor Staining Buffer Set. Data acquisition was performed on a BD FACS Canto flow cytometer, and results were analyzed using FlowJo software.

**Cytometric Bead Array**

Serum levels of TNF, IL-6 and IFN-γ were quantified using the Mouse Th1/Th2/Th17 CBA Kit (BD Biosciences) following the manufacturer’s protocol. Prepared serum samples and standard dilutions were processed as instructed. Briefly, 50 μL of mixed capture beads were added to each tube, followed by 50 μL of standard or sample. Then, 50 μL of PE detection reagent was added to all tubes. After thorough mixing, the tubes were incubated for 3 h at room temperature in the dark. Following incubation, 500 μL of wash buffer was added, and the tubes were centrifuged at 300 g for 5 min. The supernatant was aspirated, and the beads were resuspended in 300 μL wash buffer. Data acquisition was performed using a flow cytometer and analyzed with FCAP Array software (FCS 2.0 format).

**
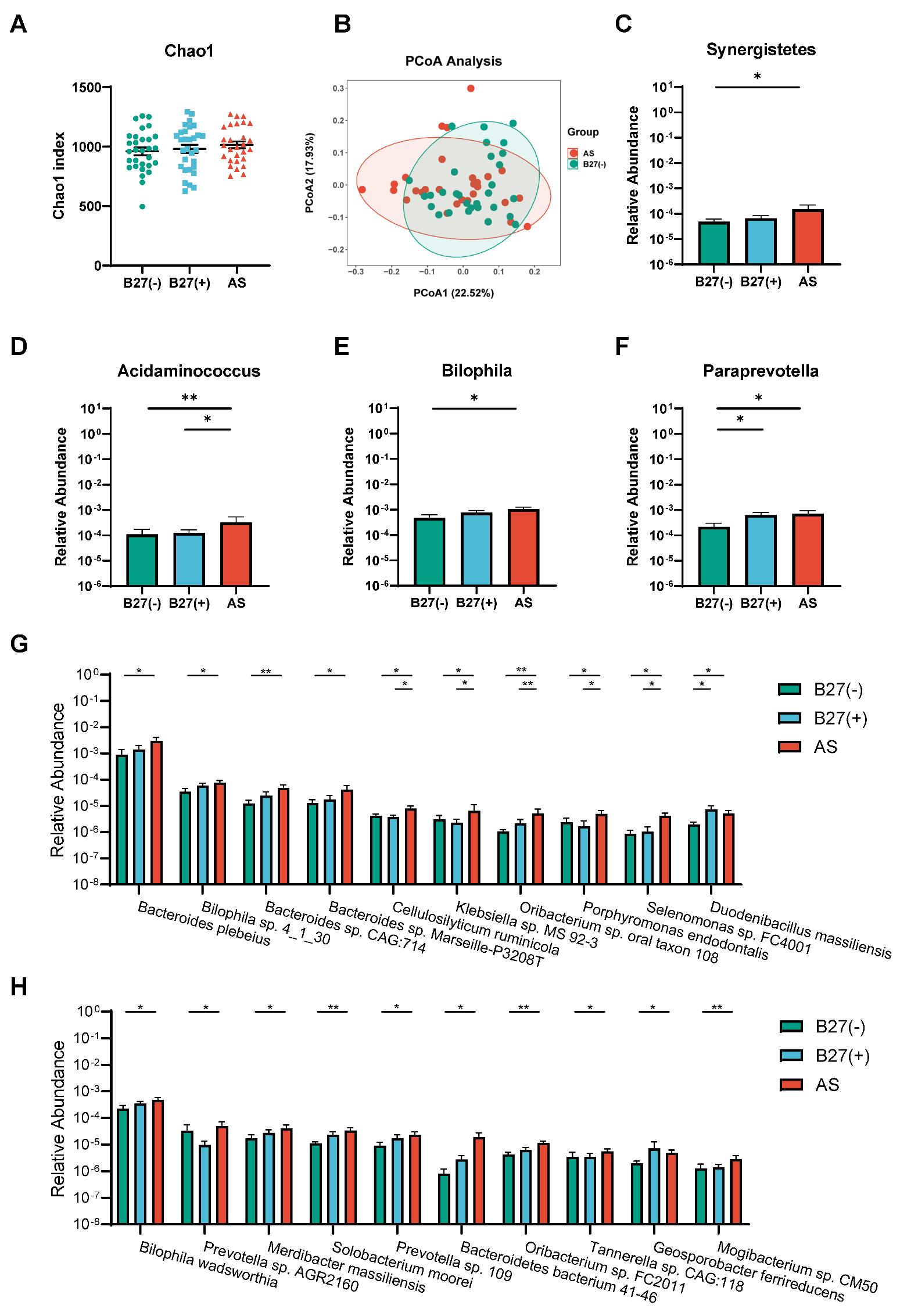
**

**Fig. S1. Gut microbiota signatures among AS, B27(+) and B27(-) groups.** (**A**): Alpha diversity measured by Chao index showed no difference among the three groups. (**B**): Principal coordinate analysis (PCoA) showed gut microbial composition of AS was significantly different than that of B27(-) (Bray-Curtis distance, ANOSIM, B27(-) vs AS, P= 0.027). Bar plots showed altered bacteria taxa in AS compared with B27(+) and B27(-) at the phylum level (**C**) and the genus level (**D-F**). (**G-H**): The relative abundance of species increased in AS compared with B27(+) and B27(-). *P<0.05, **P<0.01 as determined by Kruskal-Wallis post-hoc test. AS, ankylosing spondylitis. B27(+) referred to HLA-B27-positive healthy controls; B27(-) referred to HLA-B27-negative healthy controls.

**
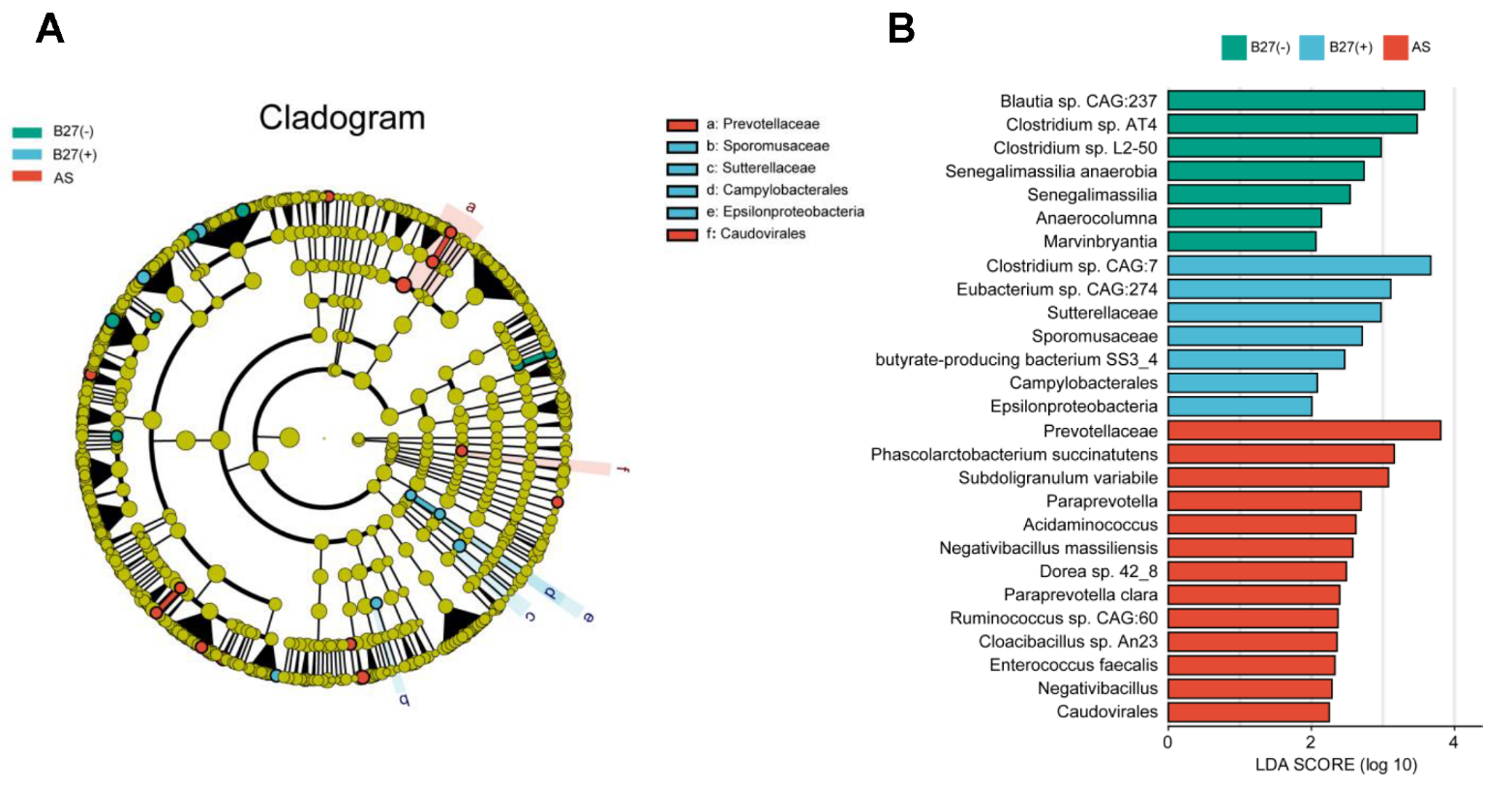
**

**Fig. S2. Linear discriminant analysis Effect Size (LEfSe) among AS, B27(+) and B27(-) groups.** (**A**): Cladogram (LDA score > 2.0, P < 0.05) showed the taxonomic distribution difference between the three groups, indicating with different color region. Each successive circle represents a differentially abundant taxonomic clades at phylum, class, order, family, genus and species level from the inner to outer rings. (**B**): Bar plot of the different abundant taxa based on the cutoff value of LDA score (log10) > 2.0 and P < 0.05 among the three groups.

**
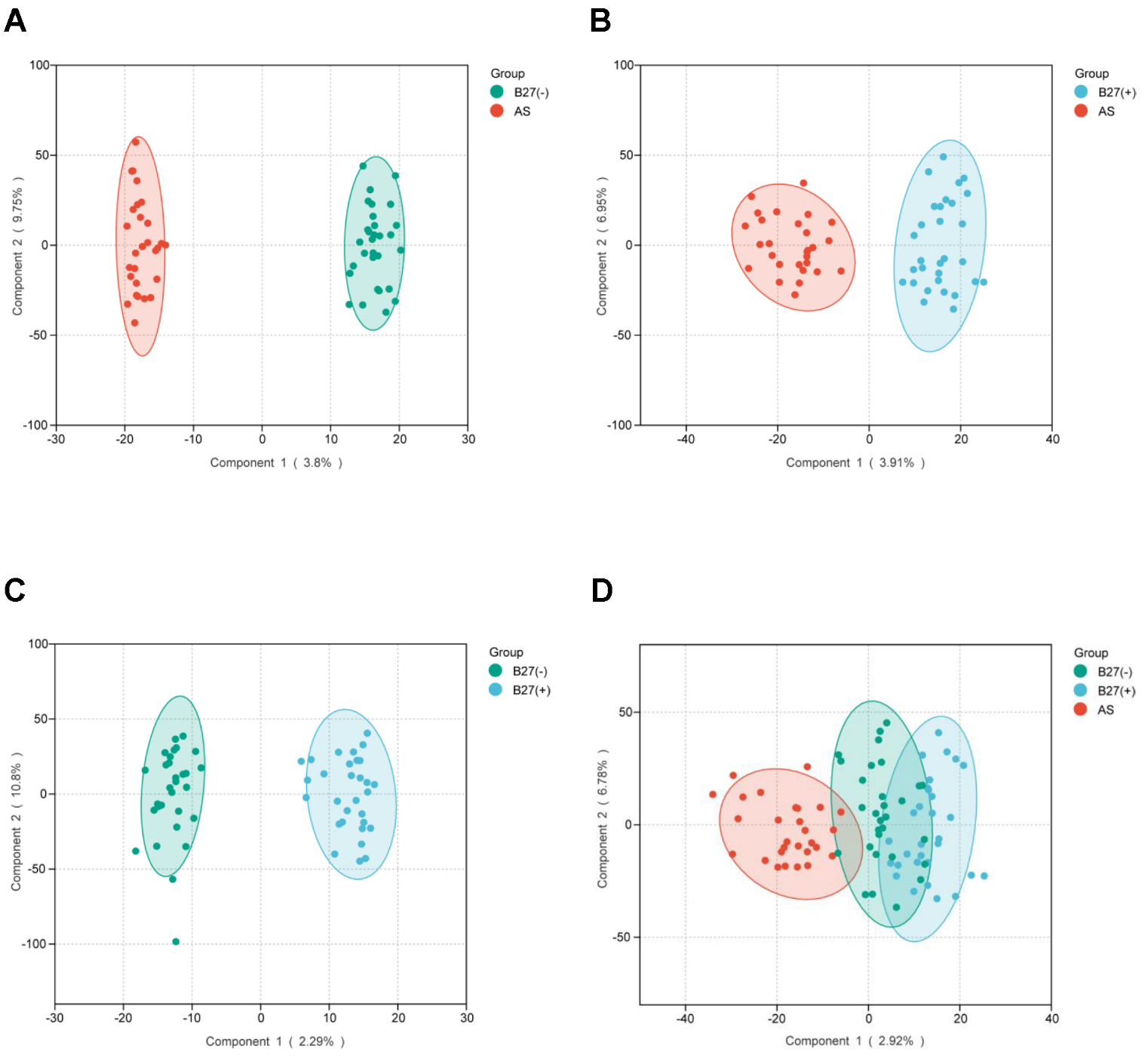
**

**Fig. S3. OPLS-DA of fecal metabolites among AS, B27(+) and B27(-) groups.** (**A**): B27(-) vs AS (R^2^Y = 0.989, Q^2^ = 0.696, P < 0.005). (**B**): B27(+) vs AS (R^2^Y = 0.915, Q^2^ = 0.584, P < 0.005). (**C**): B27(-) vs B27(+) (R^2^Y = 0.962, Q^2^ = 0.392, P < 0.005). (**D**): B27(-) vs B27(+) vs AS (R^2^Y = 0.804, Q^2^ = 0.439, P < 0.005).

**
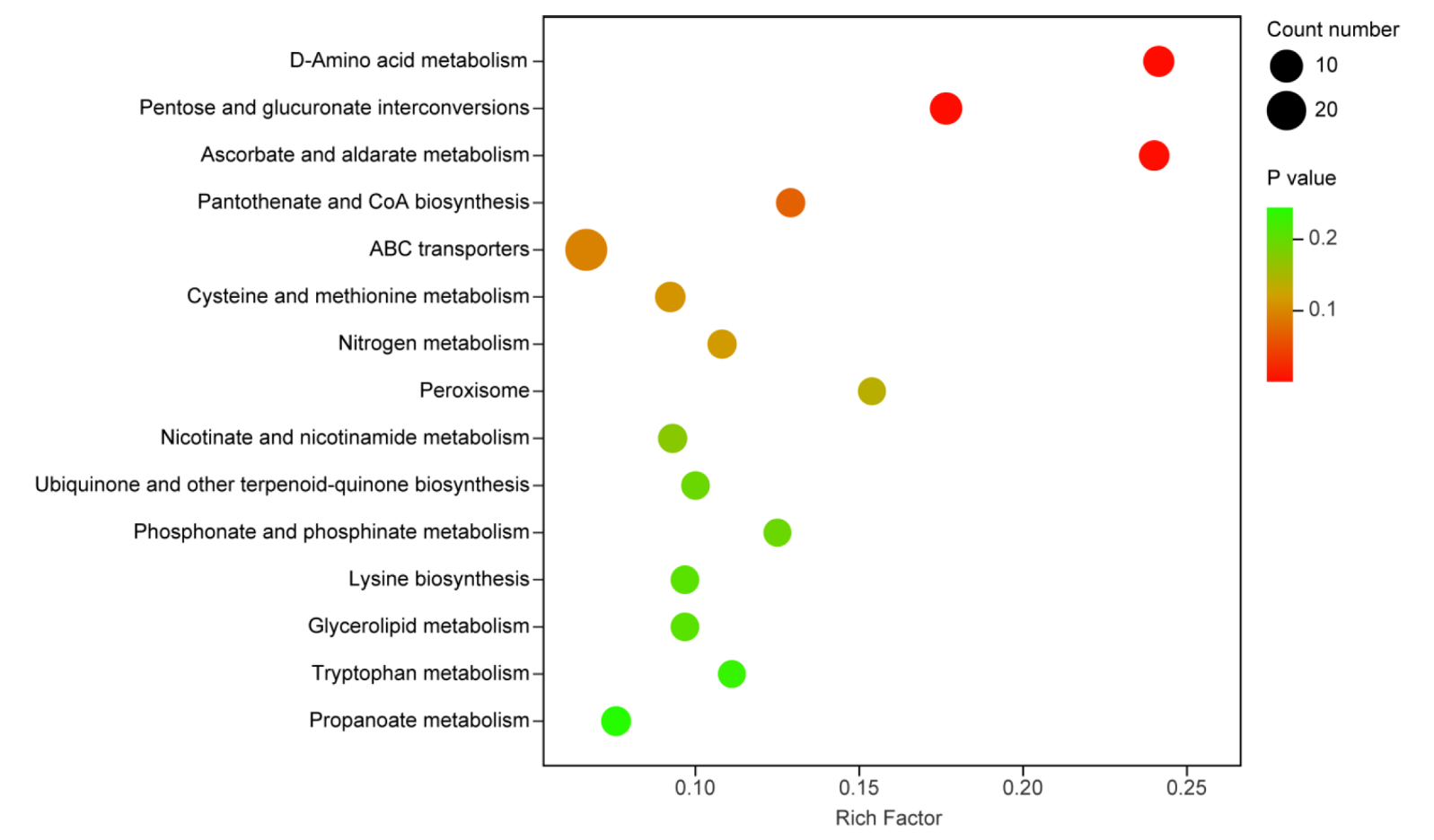
**

**Fig. S4. Biological processes enriched by differential microbial genes among AS, B27(+) and B27(-) groups.**

**
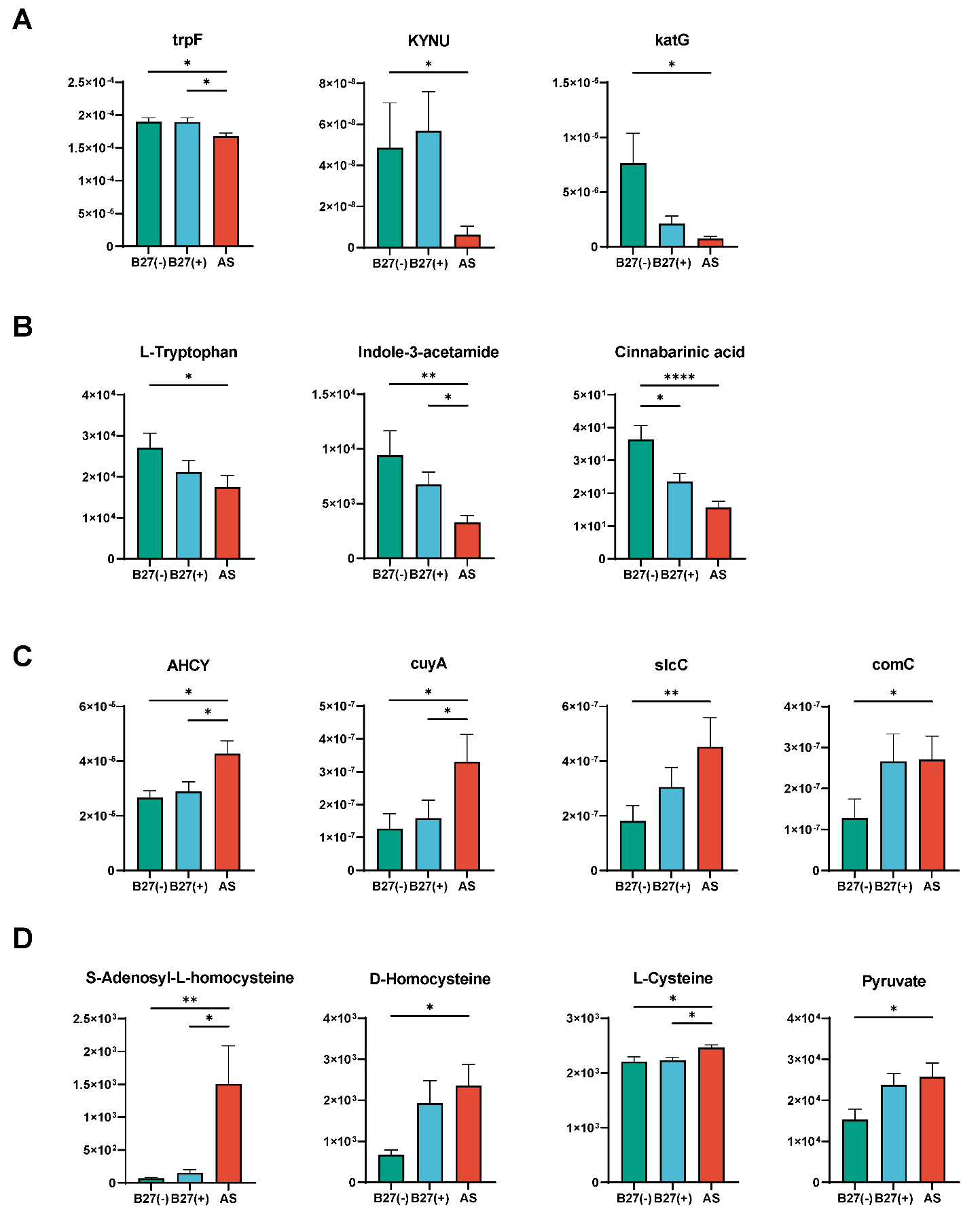
**

**Fig. S5. Perturbation of tryptophan and cysteine metabolism in AS with HLA-B27 background.**

Bar plots showed the relative abundances of differential microbial genes and absolute abundances of differential fecal metabolites in tryptophan (**A-B**) and cysteine (**C-D**) metabolism pathways among AS, B27(+) and B27(-) groups. *P<0.05, **P<0.01, ****P<0.0001 as determined by Kruskal-Wallis post-hoc test.

**
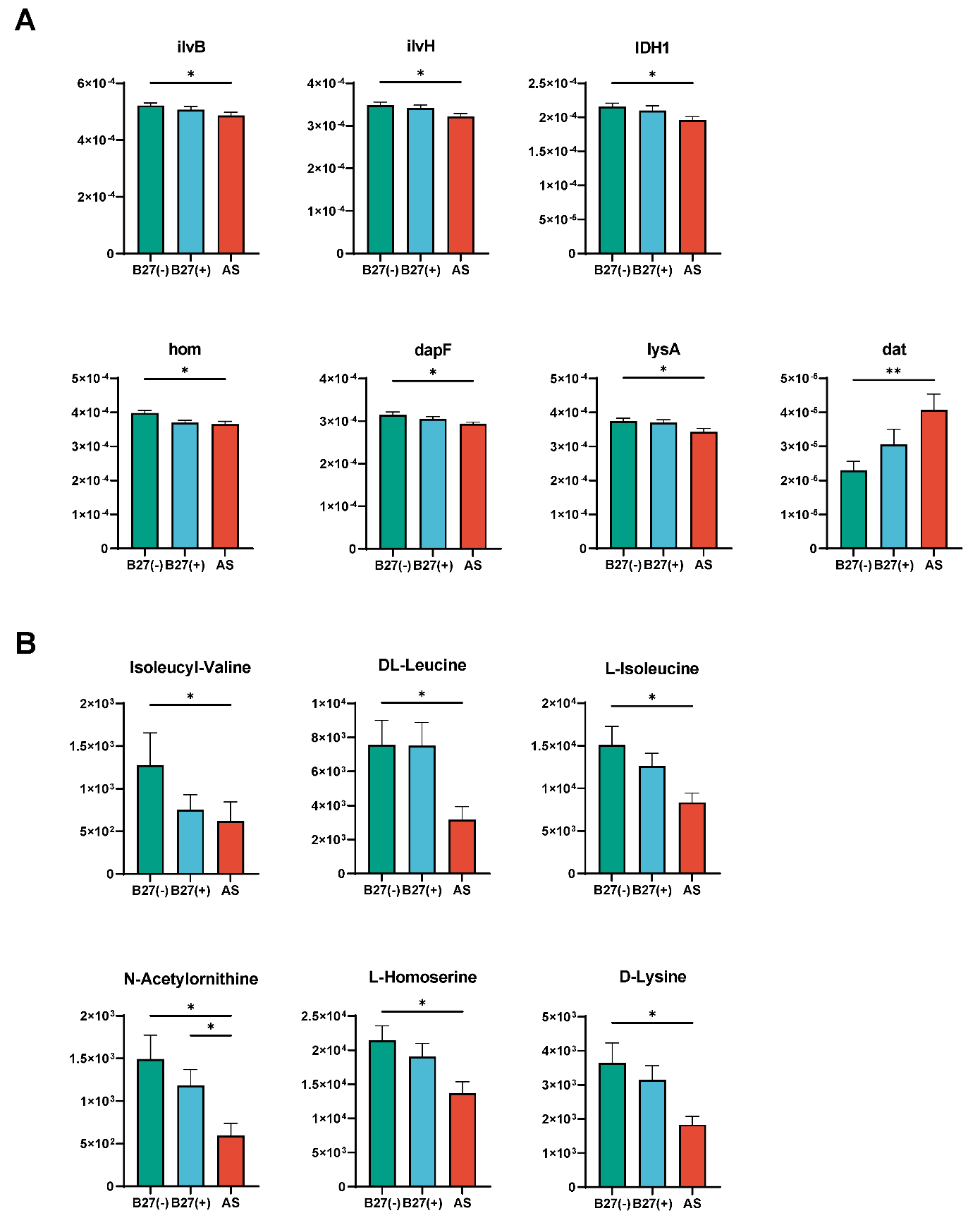
**

**Fig. S6. Perturbation of branched-chain amino acid, ornithine, and lysine biosynthesis in AS with HLA-B27 background**

Bar plots showed the relative abundances of differential microbial genes (**A**) and absolute abundances of differential fecal metabolites (**B**) in the biosynthesis pathways of branched-chain amino acid, ornithine, and lysine among AS, B27(+) and B27(-) groups. *P<0.05, **P<0.01, as determined by Kruskal-Wallis post-hoc test.

**
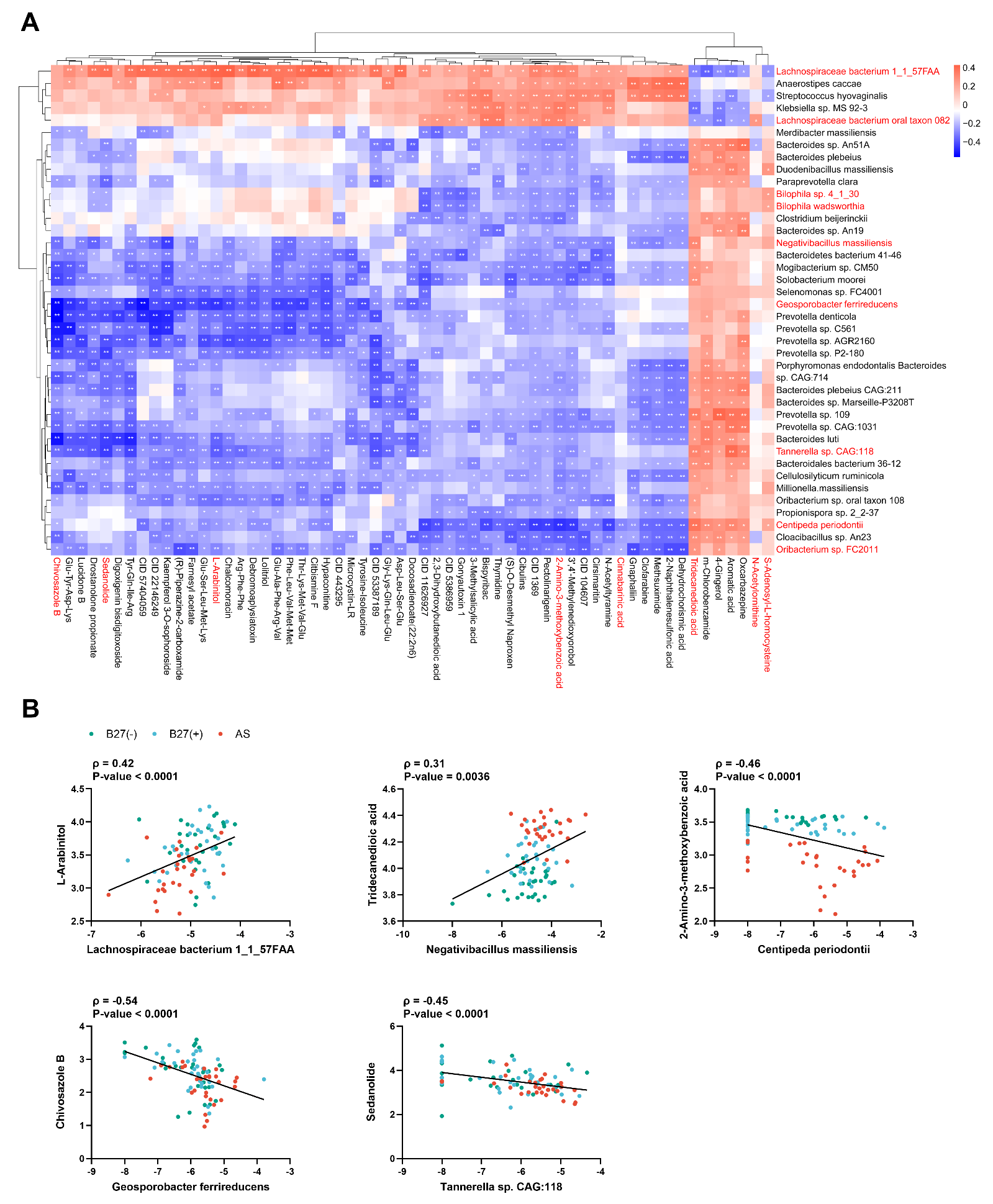
**

**Fig. S7. Associations of the disease-related microbiota and metabolites.** (**A**): The heatmap depicted associations between the species and metabolites altered in AS group (Spearman rank correlation test). (**B**): Examples of individual species–metabolite correlations. For visualization, abundances of species and metabolites are plotted after log10-transformation, and 0 values were assigned 1e-08. Each dot represents one sample. *P<0.05, **P<0.01.


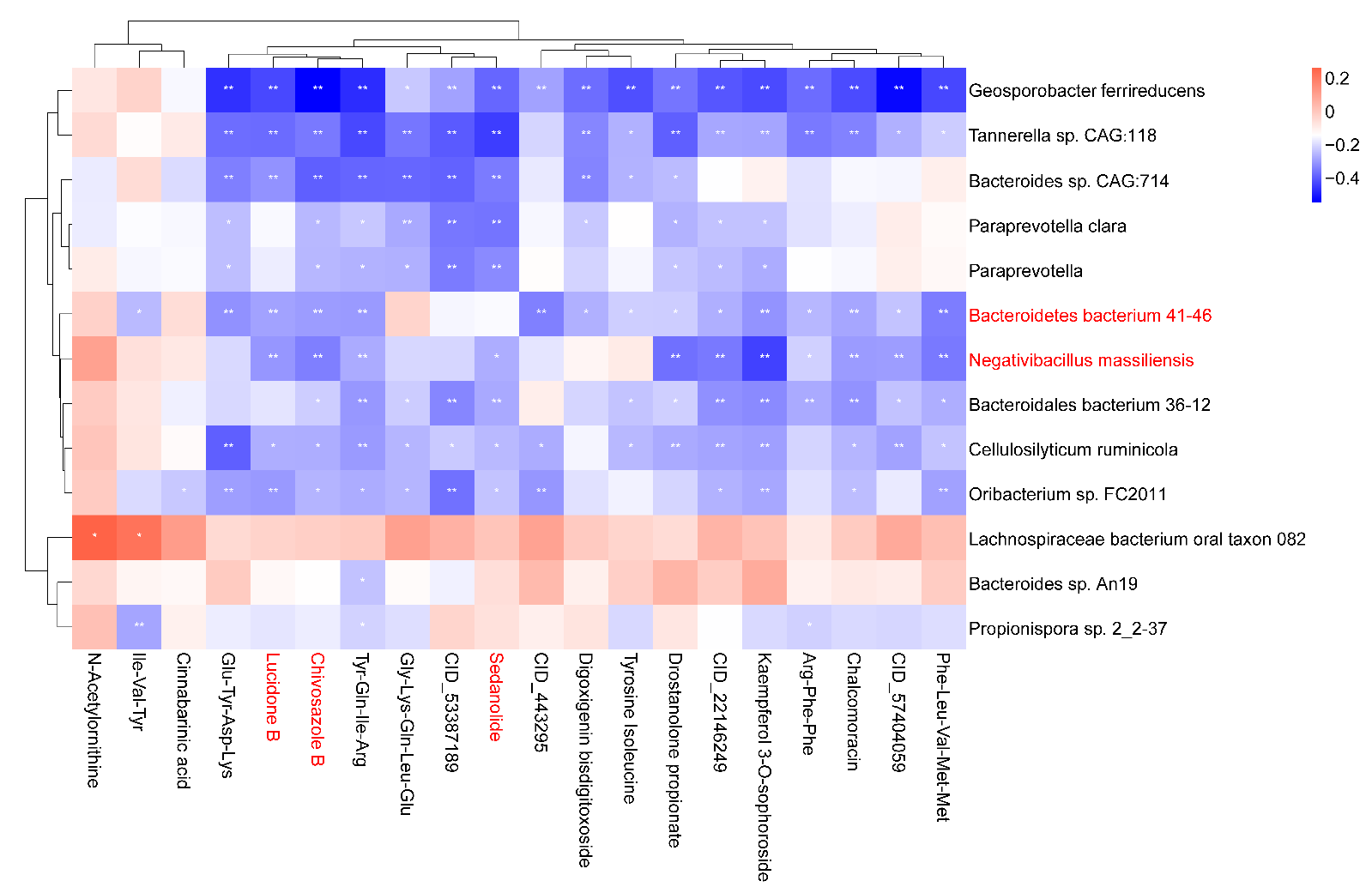


**Fig. S8. Associations of the AS clinical indices-related microbiota and metabolites.** Spearman correlations (heatmap) between microbial species and metabolites related to BASDAI, BASFI, ASDAS-CRP, and CRP are shown. *p < 0.05, **p < 0.01.

**
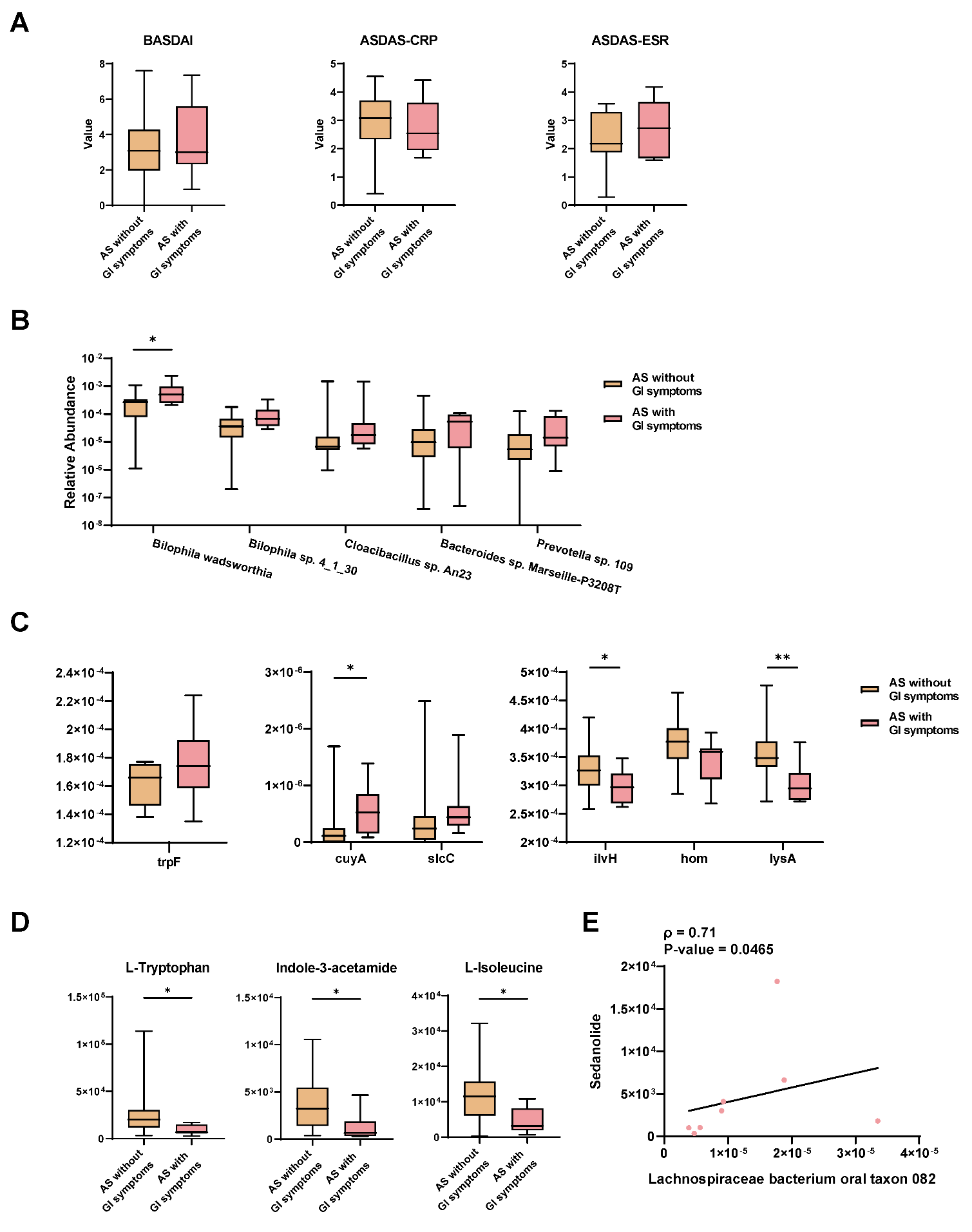
**

**Fig. S9. Subgroup analysis of AS patients stratified by gastrointestinal symptoms.** Subgroup analyses comparing patients with and without gastrointestinal symptoms across multiple levels: (**A**) disease activity indices, (**B**) microbial species, (**C**) KO gene abundances, and (**D**) metabolite levels. Panel (**E**) shows species–metabolite correlation specific to the symptomatic subgroup. *P < 0.05, **P < 0.01.


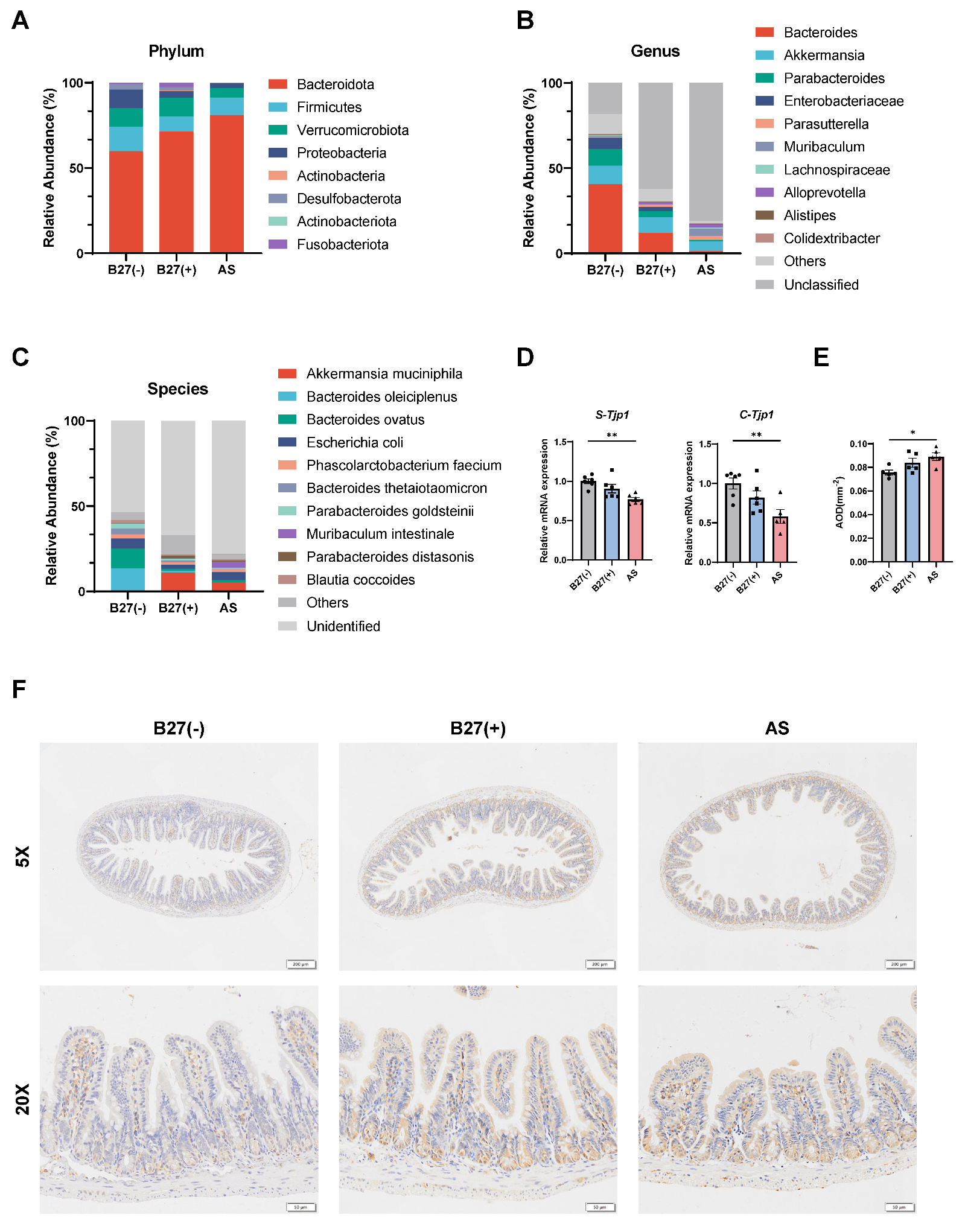


**Fig. S10.** **AS Patient-Derived Microbiota Promotes Macrophage-Mediated Intestinal Inflammation.** (**A**)-(**C**): The top 10 relative abundance of bacterial taxa at the (**A**) phylum, (**B**) genus and (**C**) species levels. (**D**) RT-qPCR analysis of *Tjp1* in intestinal tissue. S indicates small intestine, C indicates colon. (**E**)-(**F**): Quantification of F4-80-positive cell density (**E**) and representative immunohistochemical staining of F4/80 in small intestine tissue (**F**). Data represent the mean ± SEM (n = 5-6). *P < 0.05, **P < 0.01.


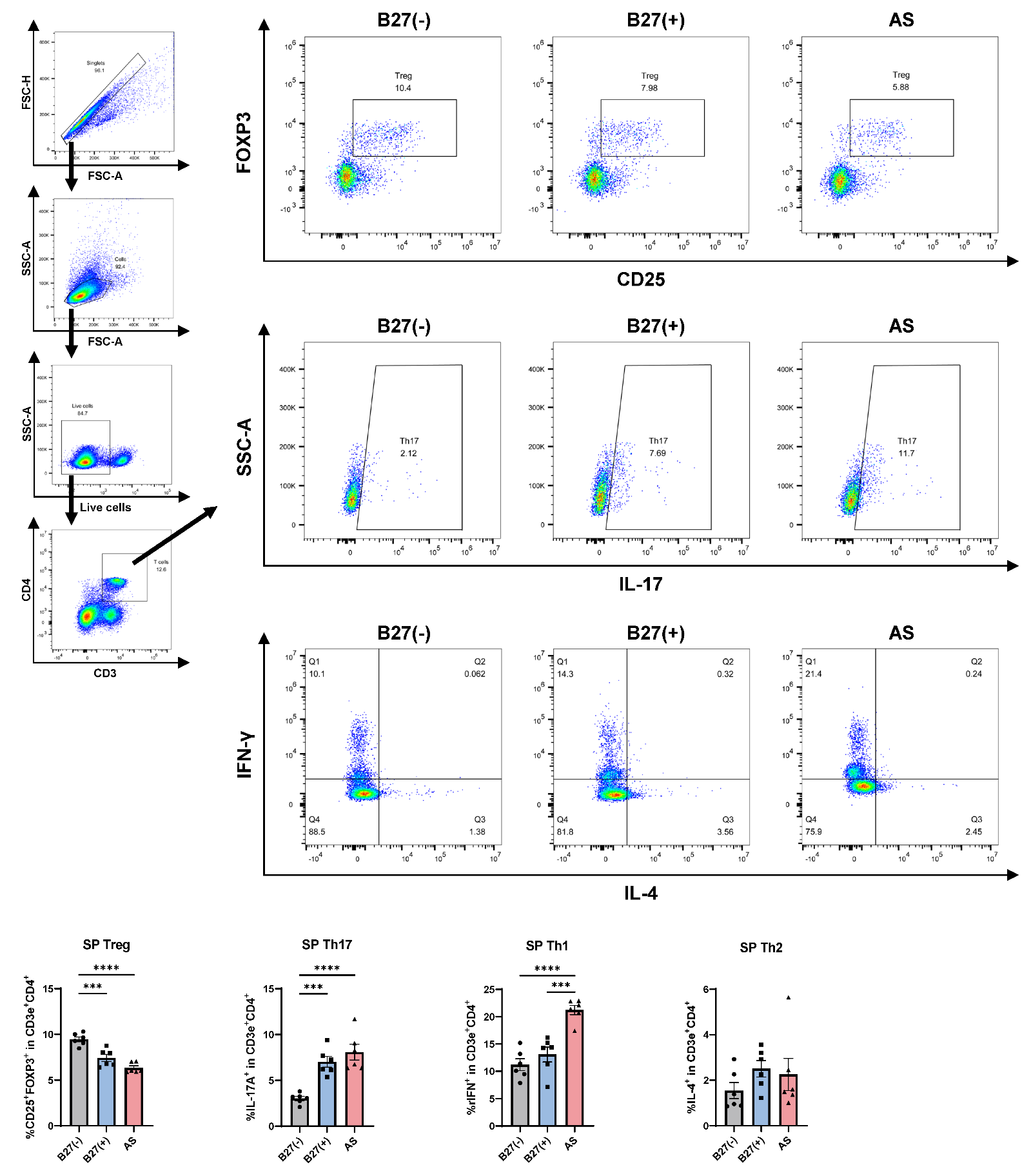


**Fig. S11. Flow cytometry of splenic T cells.** Data represent the mean ± SEM (n = 6). ***P < 0.001, ****P < 0.0001. SP indicates spleen.


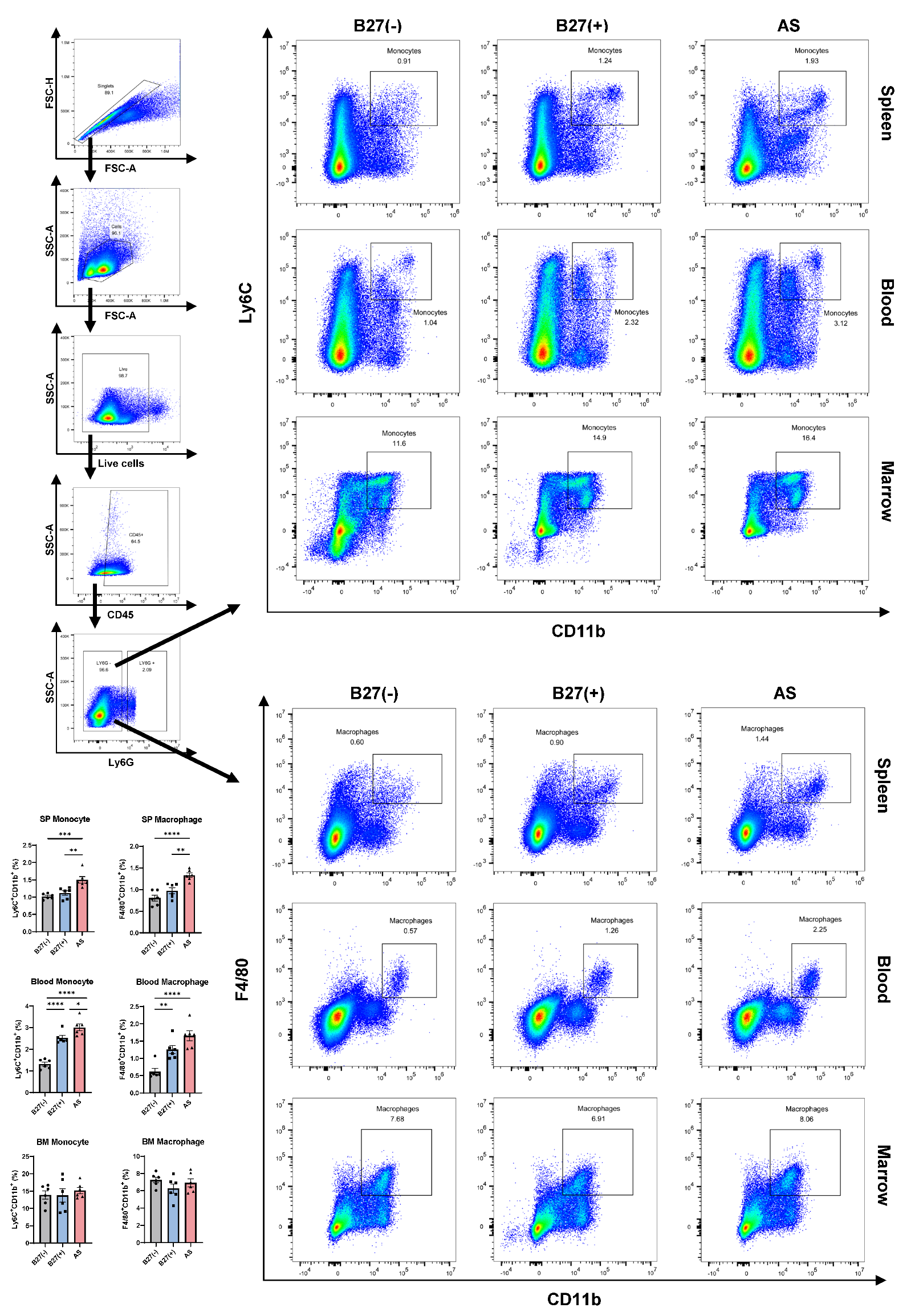


**Fig. S12. Flow cytometry of mononuclear-macrophages in spleen, peripheral blood and bone marrow.** Data represent the mean ± SEM (n = 6). *P < 0.05, **P < 0.01, ***P < 0.001, ****P < 0.0001. SP indicates spleen; Blood indicates peripheral blood; BM indicates bone marrow.


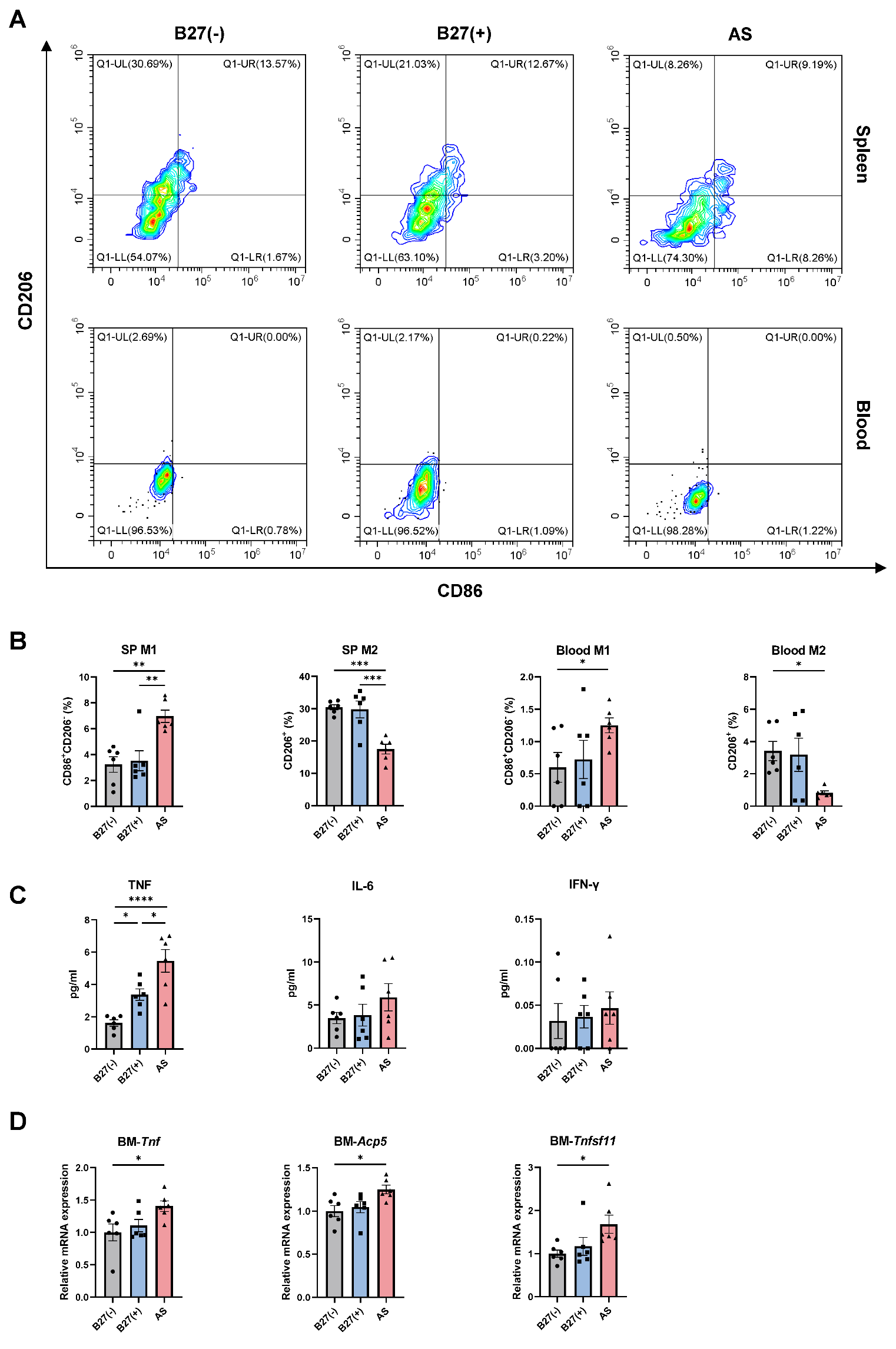


**Fig. S13 AS Patient-Derived Microbiota Promotes Systemic Inflammation.** (**A**) Flow cytometry of M1/M2 macrophages in spleen and peripheral blood. (**B**) Frequencies in spleen and peripheral blood across experimental groups. (**C**) Quantification of serum inflammatory cytokine levels using cytometric bead array (CBA). (**D**) RT-qPCR analysis in bone marrow. Data represent the mean ± SEM (n = 6). *P < 0.05, **P < 0.01, ***P < 0.001, ****P < 0.0001. SP indicates spleen; Blood indicates peripheral blood; BM indicates bone marrow.

**
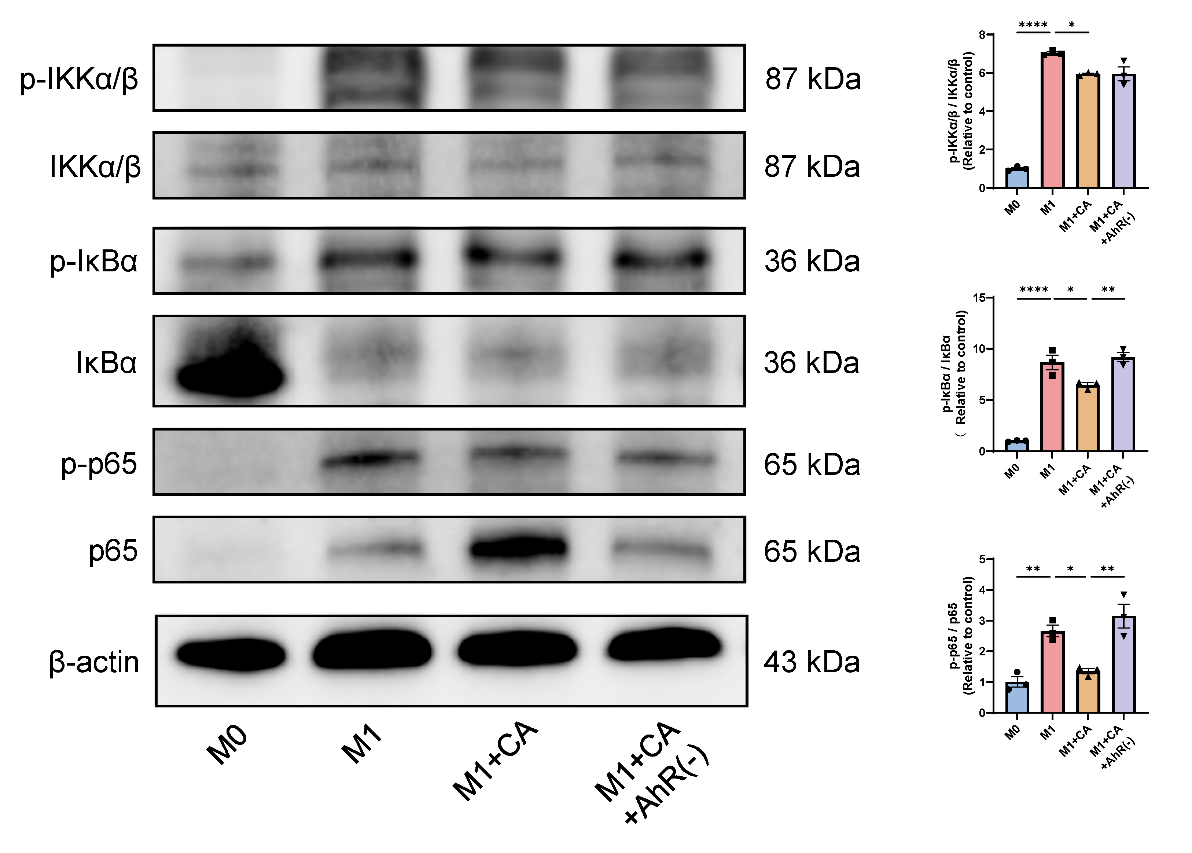
**

**Fig. S14 Western blot analysis of the NF-κB signaling pathway in BMDMs under M1 polarization conditions.** Data are presented as mean ± SEM (n = 3). *P < 0.05, **P < 0.01, ****P < 0.0001.


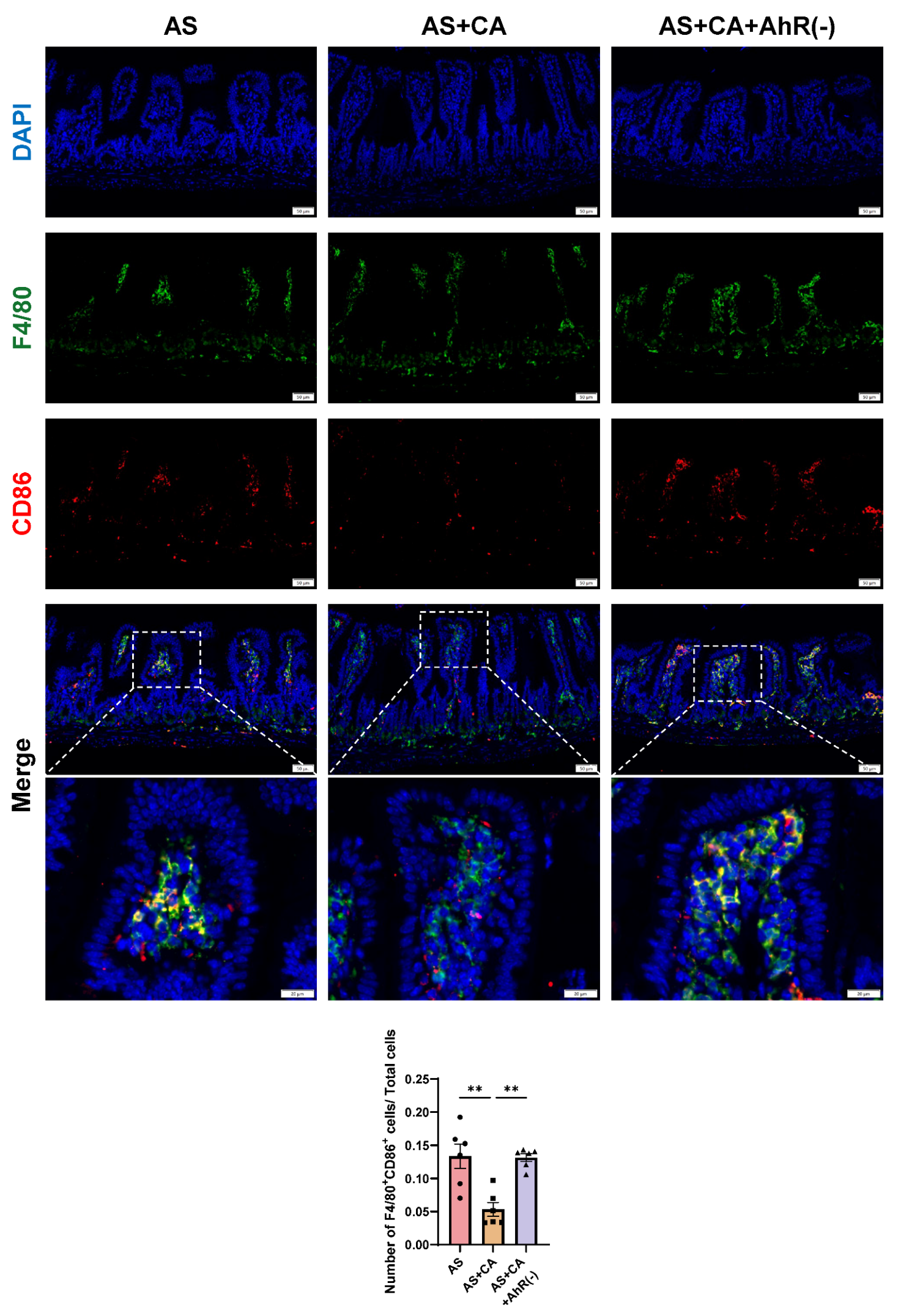


**Fig. S15 Immunofluorescence staining of F4/80 and CD86 in small intestine tissue.** Data are presented as mean ± SEM (n = 6). **P < 0.01.


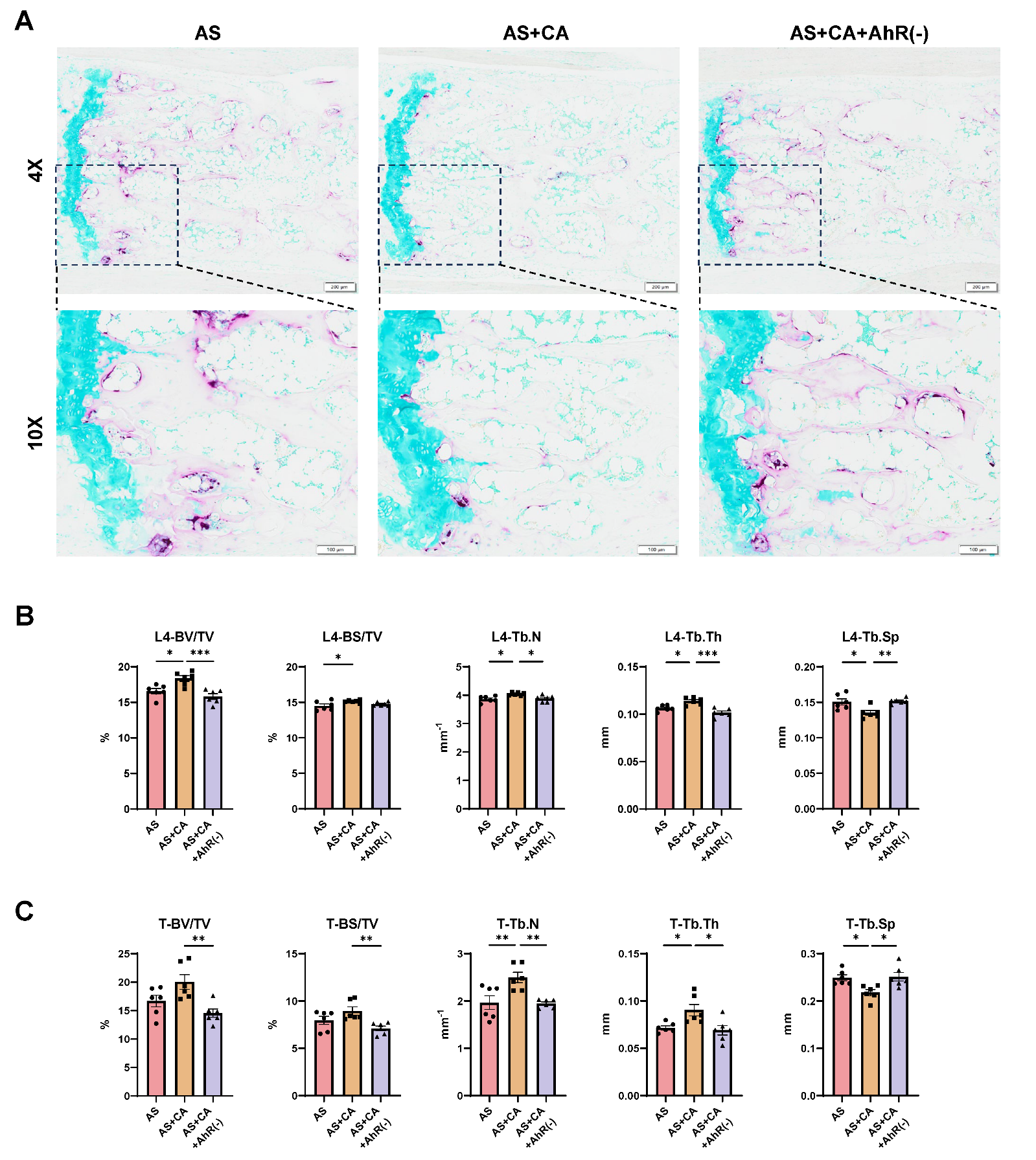


**Fig. S16 CA promotes bone mass in the AS-FMT model.** (**A**) Representative TRAP staining images of caudal vertebrae. **B-C**: Quantitative analysis of bone mass and trabecular parameters in the fourth lumbar vertebra (**B**) and tibia (**C**). Data represent the mean ± SEM (n = 6). *P < 0.05, **P < 0.01, ***P < 0.001. L4 indicates fourth lumbar vertebra; T indicates tibia.

Table S1 Demographic and clinical characteristics of the AS patients

| Patient ID | Gender | Age  (years) | Disease duration (years) | BASDAI | ASDAS-CRP | ASDAS-ESR | BASFI | CRP  (μg/L) | ESR  (mm/h) | Family History  (Y: yes, N:no) |
| --- | --- | --- | --- | --- | --- | --- | --- | --- | --- | --- |
| 01 | Male | 24 | 1 | 2.2 | 3.24 | NA | 3.2 | 13.2 | NA | N |
| 02 | Male | 35 | 15 | 2.25 | 2.33 | NA | 0.5 | 6.72 | NA | Y |
| 03 | Male | 24 | 5 | 2.4 | 2.68 | NA | 0.3 | 8.53 | NA | Y |
| 04 | Male | 32 | 14 | 0.9 | 1.96 | 2.10 | 0 | 12.4 | 35 | N |
| 05 | Male | 47 | 33 | 0 | 0.40 | 0.29 | 0 | 1 | 1 | N |
| 06 | Male | 40 | 3 | 3 | 2.46 | 2.17 | 1 | 19 | 28 | N |
| 07 | Male | 29 | 2 | 0.8 | 1.07 | 1.28 | 0 | 1.3 | 8 | Y |
| 08 | Male | 33 | 12 | 1.9 | 2.46 | 2.17 | 0.2 | 19 | 28 | N |
| 09 | Male | 33 | 3 | 2.05 | 2.29 | NA | 2.2 | 13.2 | NA | Y |
| 10 | Male | 24 | 3 | 1.95 | 2.27 | 1.87 | 0.1 | 5.59 | 8 | Y |
| 11 | Male | 44 | 1 | 3.15 | 2.99 | 3.50 | 4.2 | 12.5 | 56 | N |
| 12 | Male | 36 | 4 | 3.25 | 1.94 | 1.66 | 0.7 | 1.44 | 2.5 | N |
| 13 | Male | 39 | 1 | 4.2 | 3.21 | 3.25 | 1.4 | 26 | 51 | N |
| 14 | Male | 25 | 2 | 5.35 | 4.30 | 1.95 | 2.9 | 38.4 | 0 | N |
| 15 | Male | 37 | 9 | 2.7 | 2.83 | 2.17 | 0 | 13.96 | 8 | N |
| 16 | Male | 25 | 3 | 3.7 | 4.01 | 3.03 | 2.1 | 24.4 | 16 | Y |
| 17 | Male | 29 | 2 | 2.75 | 2.79 | 1.60 | 0.4 | 14.47 | 2 | Y |
| 18 | Male | 37 | 4 | 7.35 | 3.89 | 4.18 | 1.5 | 8.73 | 37 | N |
| 19 | Male | 48 | 5 | 7.6 | 4.09 | 3.59 | 0.1 | 5.53 | 7 | Y |
| 20 | Male | 37 | 4 | 5.1 | 4.41 | 3.65 | 3.4 | 75.1 | 44 | N |
| 21 | Male | 25 | 1 | 5.85 | 4.55 | 3.31 | 9.7 | 26.8 | 10 | N |
| 22 | Male | 35 | 14 | 4.3 | 3.41 | NA | 0.7 | 17.7 | NA | N |
| 23 | Female | 33 | 1 | 1.35 | 1.50 | 1.88 | 0 | 3.15 | 18 | Y |
| 24 | Female | 34 | 2 | 5.6 | 3.16 | NA | 4 | 3.02 | NA | N |
| 25 | Female | 26 | 4 | 3.75 | 3.75 | 3.29 | 3 | 52.5 | 47 | Y |
| 26 | Female | 21 | 5 | 3.55 | 3.56 | 2.45 | 1.6 | 32 | 15.3 | N |
| 27 | Female | 34 | 7 | 2.5 | 1.68 | 2.72 | 1.1 | 1 | 34 | N |
| 28 | Female | 31 | 2 | 5.75 | 2.75 | 3.39 | 1.2 | 2.77 | 29 | N |

NA, not available.

Table S2 Primers sequences for quantitative RT-PCR

| Primers | Forward/Reverse | Sequences |
| --- | --- | --- |
| *Gapdh* | F | 5’- CATCACTGCCACCCAGAAGACTG -3’ |
|  | R | 5’- ATGCCAGTGAGCTTCCCGTTCAG -3’ |
| *Tnf* | F | 5’- CAGGCGGTGCCTATGTCTC -3’ |
|  | R | 5’- CGATCACCCCGAAGTTCAGTAG -3’ |
| *Il6* | F | 5’- TACCACTTCACAAGTCGGAGGC -3’ |
|  | R | 5’- CTGCAAGTGCATCATCGTTGTTC -3’ |
| *Il1b* | F | 5’- TGCCACCTTTTGACAGTGATG -3’ |
|  | R | 5’- TGATGTGCTGCTGCGAGATT -3’ |
| *Il10* | F | 5’- CGGGAAGACAATAACTGCACCC -3’ |
|  | R | 5’- CGGTTAGCAGTATGTTGTCCAGC -3’ |
| *Tjp1* | F | 5’- ACCCGAAACTGATGCTGTGGATAG -3’ |
|  | R | 5’- AAATGGCCGGGCAGAACTTGTGTA -3’ |
| *Cd86* | F | 5’- ACGTATTGGAAGGAGATTACAGCT -3’ |
|  | R | 5’- TCTGTCAGCGTTACTATCCCGC -3’ |
| *Nos2* | F | 5’- CAGCTGGGCTGTACAAACCTT -3’ |
|  | R | 5’- CATTGGAAGTGAAGCGTTTCG -3’ |
| *Mrc1* | F | 5’- GGTGCTACTCCGAACAACAG -3’ |
|  | R | 5’- ACCGTGGCTGAAAGTTCCT -3’ |
| *Arg1* | F | 5’- CATTGGCTTGCGAGACGTAGAC -3’ |
|  | R | 5’- GCTGAAGGTCTCTTCCATCACC -3’ |
| *Acp5* | F | 5’- AGCAGCCAAGGAGGACTAC -3’ |
|  | R | 5’- CATAGCCCACACCGTTCTC -3’ |
| *Tnfsf11* | F | 5’- ACCAAGATGGCTTCTATTACC -3’ |
|  | R | 5’- TCCCTCCTTTCATCAGGTTAT -3’ |

Table S3 Dietary preferences of the study participants

| Characteristics | AS  (n=28) | HLA-B27(+) HC  (n=30) | HLA-B27(-) HC  (n=30) | P-value |
| --- | --- | --- | --- | --- |
| Taste Preference, n (%) |  |  |  | 0.343 |
| Neutral | 13 (46.4) | 19 (63.3) | 22 (73.3) | - |
| Mild | 2 (7.1) | 1 (3.3) | 1 (3.3) | - |
| Spicy | 8 (28.6) | 3 (10) | 4 (13.3) | - |
| Greasy/Oily | 3 (10.7) | 4 (13.3) | 1 (3.3) | - |
| Salty | 1 (3.6) | 3 (10) | 1 (3.3) | - |
| Sweet | 1 (3.6) | 0 (0) | 0 (0) | - |
| Food Preference, n (%) |  |  |  | 0.131 |
| High-sugar foods | 8 (13.8) | 14 (18.2) | 3 (4.5) | - |
| High-fat foods | 22 (37.9) | 21 (27.3) | 16 (24.2) | - |
| Fruits and vegetables | 13 (22.4) | 19 (24.7) | 25 (37.9) | - |
| Legumes and dairy | 10 (17.2) | 17 (22.1) | 18 (27.3) | - |
| Preserved and processed foods | 5 (8.6) | 6 (7.8) | 4 (6.1) | - |

Table S4 The metabolites differentially abundant between AS and HLA-B27(-) HC

| Compounds | VIP | P value | FDR | Fold Change  (AS/HLA-B27(-) HC) | Change |
| --- | --- | --- | --- | --- | --- |
| Sarpogrelate hydrochloride | 1.722485 | 0.001557 | 0.038281 | 0.329243 | Down |
| Cisapride hydrate | 2.434715 | 0.000168 | 0.007627 | 0.352451 | Down |
| 2-Amino-3-methoxybenzoic acid | 4.895561 | 6.88E-17 | 6.79E-14 | 0.216772 | Down |
| Pectolinarigenin | 4.845515 | 6.88E-17 | 6.79E-14 | 0.31542 | Down |
| (S)-O-Desmethyl Naproxen | 4.333443 | 1.27E-12 | 3.71E-10 | 0.305792 | Down |
| Prostaglandin B2 | 1.707174 | 0.000809 | 0.023924 | 0.460618 | Down |
| 2,4-Dihydroxybenzoic acid | 3.935481 | 3.97E-13 | 1.42E-10 | 0.326909 | Down |
| 2-Naphthalenesulfonic acid | 2.622678 | 1.22E-11 | 2.60E-09 | 0.407069 | Down |
| 8-Anilino-1-naphthalenesulfonic acid | 4.837038 | 6.88E-17 | 6.79E-14 | 0.323933 | Down |
| Pindone | 1.607211 | 0.000431 | 0.015395 | 0.460559 | Down |
| 3-[4-(2-methylbutan-2-yl)phenoxy]benzoic acid | 1.882977 | 6.09E-05 | 0.003269 | 0.362626 | Down |
| 4-O-8',5'-5''-Dehydrotriferulic acid | 3.448922 | 8.34E-14 | 3.87E-11 | 0.115119 | Down |
| Benzenesulfonamide,2-(cyclohexylamino)-5-nitro-N-[(pentylamino)carbonyl]- | 2.285301 | 0.000354 | 0.013195 | 0.365348 | Down |
| Butralin | 2.024306 | 0.001557 | 0.038281 | 0.2135 | Down |
| Butylated hydroxytoluene | 2.044497 | 0.001386 | 0.035411 | 0.398669 | Down |
| Dehydrochorismic acid | 2.605491 | 8.29E-12 | 1.87E-09 | 0.415207 | Down |
| Fusaroskyrin | 2.889015 | 4.36E-06 | 0.000378 | 0.249362 | Down |
| Imazamethabenz-methyl | 2.228571 | 0.000222 | 0.009305 | 0.488495 | Down |
| Isopropalin | 1.804498 | 0.000971 | 0.027377 | 0.377996 | Down |
| Manidipine | 2.812121 | 1.05E-05 | 0.000744 | 0.268409 | Down |
| N-[4-[[6-Methoxy-7-[3-(4-morpholinyl)propoxy]-4-quinazolinyl]amino]phenyl]benzamide | 2.396387 | 6.22E-06 | 0.000482 | 0.260504 | Down |
| Pamoic acid | 2.524524 | 1.59E-05 | 0.001082 | 0.458253 | Down |
| Probucol | 1.514009 | 2.21E-05 | 0.001416 | 0.43392 | Down |
| Protohypericin | 3.735451 | 5.24E-11 | 9.62E-09 | 0.44841 | Down |
| Sulochrin | 2.874758 | 1.18E-06 | 0.000117 | 0.466515 | Down |
| Methsuximide | 2.446394 | 3.75E-10 | 6.30E-08 | 0.479477 | Down |
| Argenteane | 1.70423 | 0.001162 | 0.031519 | 0.284805 | Down |
| Isosinomenine A | 1.654257 | 0.001649 | 0.039562 | 0.402632 | Down |
| (3E)-4-(1-Hydroxy-2,6,6-trimethyl-4-oxocyclohex-2-en-1-yl)but-3-en-2-yl 6-O-pentopyranosylhexopyranoside | 2.356711 | 0.00011 | 0.005253 | 0.390347 | Down |
| 1,2-Dioleoyl-sn-glycero-3-phosphate | 2.293745 | 0.001308 | 0.033948 | 0.440422 | Down |
| 1,2-Ditetradecanoyl-sn-glycero-3-phosphocholine | 2.398087 | 9.50E-05 | 0.004716 | 0.369672 | Down |
| 12(S)-HETE | 1.686922 | 0.000761 | 0.023104 | 0.190829 | Down |
| 16(S)-Iloprost | 1.351515 | 0.000193 | 0.008424 | 0.180795 | Down |
| 1-Oleoyl-2-myristoyl-sn-glycero-3-phosphocholine | 1.625794 | 0.000761 | 0.023104 | 0.321306 | Down |
| 3-Methylglutarylcarnitine | 1.926641 | 3.04E-05 | 0.001817 | 0.204121 | Down |
| 7-[3-(Undeca-2,5-dien-1-yl)oxiran-2-yl]hept-5-enoic acid | 2.113055 | 0.002188 | 0.048518 | 0.46026 | Down |
| 7-Acetylbaccatin III | 2.490283 | 2.03E-05 | 0.001327 | 0.353924 | Down |
| Farnesyl acetate | 2.341875 | 0.000378 | 0.013958 | 0.335712 | Down |
| Annocherin A | 1.800072 | 0.000761 | 0.023104 | 0.175863 | Down |
| Asclepin | 2.179074 | 0.000523 | 0.017628 | 0.243209 | Down |
| Cyasterone | 2.107426 | 0.001469 | 0.036817 | 0.354394 | Down |
| Ganolucidic acid D | 1.330489 | 0.002069 | 0.046664 | 0.485576 | Down |
| Ecdysterone | 1.831857 | 3.04E-05 | 0.001817 | 0.239105 | Down |
| Khivorin | 2.003668 | 0.000557 | 0.018314 | 0.353514 | Down |
| N-Palmitoyl-D-erythro-dihydroceramide-1-phosphate | 2.015168 | 0.000809 | 0.023924 | 0.239309 | Down |
| PC(16:0/20:5(5Z,8Z,11Z,14Z,17Z)) | 1.906998 | 4.36E-06 | 0.000378 | 0.234367 | Down |
| TaflUprost (free acid) | 2.630899 | 1.88E-05 | 0.001233 | 0.349067 | Down |
| Vaccinoside | 3.51375 | 8.26E-16 | 5.93E-13 | 0.101769 | Down |
| Clofarabine | 2.668463 | 1.07E-09 | 1.66E-07 | 0.455731 | Down |
| Asn-Lys | 2.333851 | 5.65E-05 | 0.003073 | 0.442199 | Down |
| Cibulins | 4.554327 | 1.38E-16 | 1.21E-13 | 0.262263 | Down |
| Glabrin C | 2.437076 | 0.00031 | 0.012013 | 0.22302 | Down |
| Glutethimide | 2.759015 | 6.22E-06 | 0.000482 | 0.412419 | Down |
| Glu-Tyr-Lys | 1.530055 | 0.001386 | 0.035411 | 0.49614 | Down |
| Gly-Tyr-Gly | 1.85868 | 1.73E-06 | 0.000164 | 0.365578 | Down |
| Met-Pro-Arg | 2.163727 | 7.62E-05 | 0.003905 | 0.182032 | Down |
| N-Formyl-methionyl-leucyl-phenylalanine | 2.625318 | 7.41E-06 | 0.000568 | 0.316741 | Down |
| Oxiglutatione | 1.741387 | 1.05E-05 | 0.000744 | 0.408786 | Down |
| Phe-Phe-Arg | 2.03601 | 0.00011 | 0.005253 | 0.421458 | Down |
| Tyr-Tyr | 1.857544 | 0.00018 | 0.007993 | 0.486841 | Down |
| Tyrosyl-Leucine | 2.615169 | 4.77E-06 | 0.000396 | 0.355787 | Down |
| 2,3-Dihydroxybutanedioic acid | 4.42981 | 1.44E-13 | 6.30E-11 | 0.314366 | Down |
| 3,4-Dicaffeoylquinic acid | 4.127008 | 6.88E-17 | 6.79E-14 | 0.36061 | Down |
| Glyceric acid | 3.849081 | 2.57E-11 | 5.07E-09 | 0.406565 | Down |
| L-Arabinitol | 2.642911 | 3.85E-05 | 0.002232 | 0.389641 | Down |
| 5-benzyl-5-(pyridin-3-yl)imidazolidine-2,4-dione | 1.657257 | 0.000971 | 0.027377 | 0.355512 | Down |
| 8-Hydroxydesmethylclomipramine | 2.645705 | 1.84E-07 | 2.14E-05 | 0.323325 | Down |
| Acyclovir | 2.027967 | 0.001557 | 0.038281 | 0.422266 | Down |
| Cidofovir | 3.532535 | 6.88E-17 | 6.79E-14 | 0.080589 | Down |
| Cinnabarinic acid | 2.660714 | 1.30E-06 | 0.000128 | 0.429374 | Down |
| Cyclothiazide | 4.234177 | 6.67E-15 | 4.05E-12 | 0.191456 | Down |
| Dihydrogambogic acid | 2.256479 | 1.73E-05 | 0.001146 | 0.352299 | Down |
| Famciclovir | 2.197846 | 0.000237 | 0.009805 | 0.45564 | Down |
| Garciduol B | 3.598445 | 1.27E-12 | 3.71E-10 | 0.4352 | Down |
| HUperzine B | 2.327796 | 0.000222 | 0.009305 | 0.429196 | Down |
| hydroxynefazodone | 1.942909 | 0.001746 | 0.041265 | 0.423241 | Down |
| Latanoprost lactone diol | 1.744634 | 6.09E-05 | 0.003269 | 0.265606 | Down |
| Malyngamide T | 2.012968 | 0.000254 | 0.010225 | 0.379469 | Down |
| Mepiprazole | 2.476397 | 2.39E-05 | 0.00151 | 0.447214 | Down |
| Mequitazine | 2.552566 | 1.46E-05 | 0.001014 | 0.432934 | Down |
| N-(Adamantan-1-yl)-1-(5-fluoropentyl)-1H-indole-3-carboxamide | 1.829718 | 0.001469 | 0.036817 | 0.395487 | Down |
| Neoacrimarine H | 1.704277 | 2.76E-06 | 0.000254 | 0.375178 | Down |
| Oxypurinol | 1.546084 | 0.000207 | 0.00883 | 0.420341 | Down |
| Penitrem A | 2.094558 | 0.000207 | 0.00883 | 0.384395 | Down |
| Raltegravir | 1.857643 | 0.001162 | 0.031519 | 0.427589 | Down |
| Urobilinogen | 2.021478 | 0.001386 | 0.035411 | 0.264914 | Down |
| Zaleplon | 2.26696 | 0.000193 | 0.008424 | 0.459891 | Down |
| {[5-hydroxy-8-methyl-6-(2-methylpropanoyl)-2-oxo-4-phenyl-2H,8H-pyrano[2,3-f]chromen-8-yl]methoxy}sulfonic acid | 3.854796 | 6.88E-17 | 6.79E-14 | 0.391095 | Down |
| 2-(3,4-Dihydroxyphenyl)-5,7-dihydroxy-4-oxo-4H-chromen-3-yl 6-deoxy-4-O-beta-D-glucopyranosyl-alpha-L-mannopyranoside | 1.716938 | 1.73E-05 | 0.001146 | 0.422679 | Down |
| 2-{2,6-dihydroxy-4-[6-hydroxy-7-(3-methylbut-2-en-1-yl)-1-benzofuran-2-yl]phenyl}-6-(2,4-dihydroxyphenyl)-4-methyl-5-(sulfooxy)cyclohex-3-ene-1-carboxylic acid | 1.554395 | 4.16E-05 | 0.002394 | 0.430173 | Down |
| 3',4'-Methylenedioxyorobol | 4.844178 | 6.88E-17 | 6.79E-14 | 0.316891 | Down |
| 4-(3,7-dimethylocta-2,6-dien-1-yl)-5-(6-hydroxy-1-benzofuran-2-yl)-3-methoxybenzene-1,2-diol | 2.371766 | 0.000523 | 0.017628 | 0.291893 | Down |
| 4-(7-Hydroxy-4-oxo-3,4-dihydro-2H-chromen-2-yl)phenyl 2-O-(3,4-dihydroxy-4-(hydroxymethyl)tetrahydrofuran-2-yl)hexopyranoside | 1.918485 | 4.74E-08 | 5.94E-06 | 0.332515 | Down |
| Epoxiconazole | 3.578806 | 1.01E-12 | 3.34E-10 | 0.237979 | Down |
| Gnaphaliin | 1.551768 | 1.09E-08 | 1.51E-06 | 0.428437 | Down |
| Mulberrofuran E | 2.166162 | 0.002069 | 0.046664 | 0.415587 | Down |
| Nutlin 3 | 1.025132 | 0.000593 | 0.01911 | 0.155476 | Down |
| Sophoraflavonoloside | 1.965292 | 1.18E-07 | 1.42E-05 | 0.355728 | Down |
| Thalsimine | 1.789532 | 0.000672 | 0.021227 | 0.388244 | Down |
| 17-phenoxy trinor PGF2 ethyl amide | 2.159669 | 8.20E-05 | 0.004177 | 0.423506 | Down |
| Ala-Asp-Phe-Asp | 2.207399 | 1.35E-05 | 0.000941 | 0.370776 | Down |
| Ala-Leu-Gly-Ile-Ser | 1.91733 | 1.57E-06 | 0.000151 | 0.340473 | Down |
| Ala-Pro-Gln | 1.79431 | 0.00046 | 0.016058 | 0.353168 | Down |
| all-trans-Nonaprenyl diphosphate | 2.339711 | 5.70E-06 | 0.00045 | 0.172755 | Down |
| Arg-Asn-Phe-Arg | 2.086702 | 0.000404 | 0.014693 | 0.246629 | Down |
| Arg-His-Gln | 1.626505 | 0.001031 | 0.028464 | 0.358215 | Down |
| Arg-Phe-Phe | 2.018516 | 0.001956 | 0.044621 | 0.280159 | Down |
| Arg-Ser-Val-Glu | 1.708728 | 0.000272 | 0.010828 | 0.352149 | Down |
| Asn-His-Val-Asp | 1.969919 | 0.000254 | 0.010225 | 0.219312 | Down |
| Asn-Leu-Ser-Phe-Gln | 1.849245 | 0.00018 | 0.007993 | 0.314159 | Down |
| Asp-His-Leu-Thr-Gln | 1.82285 | 0.00086 | 0.02515 | 0.245138 | Down |
| Asp-Ile-Ser-Glu | 1.752507 | 0.000168 | 0.007627 | 0.284759 | Down |
| Asp-Leu-Ser-Glu | 1.899515 | 0.000914 | 0.026336 | 0.328651 | Down |
| Asp-Phe-Val-Glu | 1.682004 | 0.000378 | 0.013958 | 0.281176 | Down |
| Avermectin A1b monosaccharide | 2.537574 | 0.000431 | 0.015395 | 0.299329 | Down |
| Boc-Phe(NMe)-Pro-Phe(NMe)-Gly-OMe | 1.375989 | 0.001956 | 0.044621 | 0.327452 | Down |
| Boc-Pro-Phe(NMe)-Gly-OMe | 1.706124 | 0.00018 | 0.007993 | 0.466461 | Down |
| Codonocarpine | 2.292537 | 3.29E-05 | 0.001951 | 0.434783 | Down |
| Digoxigenin bisdigitoxoside | 2.287063 | 0.000914 | 0.026336 | 0.353579 | Down |
| Disinomenine | 1.980515 | 0.001233 | 0.032764 | 0.17809 | Down |
| Endomorphin-1 | 2.465149 | 5.22E-06 | 0.00042 | 0.240971 | Down |
| Fusicoccin H | 1.855948 | 0.001308 | 0.033948 | 0.353857 | Down |
| Glu-Ser-Leu-Met-Lys | 1.990954 | 5.23E-05 | 0.002888 | 0.30956 | Down |
| Glu-Tyr-Asp-Lys | 2.213371 | 0.00086 | 0.02515 | 0.277773 | Down |
| Glu-Val-Phe-Asp-Glu | 2.161729 | 0.001956 | 0.044621 | 0.346381 | Down |
| Gly-Lys-Gln-Leu-Glu | 2.148153 | 6.56E-05 | 0.003431 | 0.456161 | Down |
| His-Tyr-Gln-Asp | 1.561355 | 0.000809 | 0.023924 | 0.324895 | Down |
| His-Val-Phe-Gly-Glu | 1.857781 | 0.00029 | 0.011349 | 0.455679 | Down |
| Ile-Tyr-Met | 2.305391 | 0.002069 | 0.046664 | 0.409046 | Down |
| Ile-Val-Tyr | 3.311034 | 2.89E-09 | 4.30E-07 | 0.429727 | Down |
| JWH182 | 2.219888 | 0.001746 | 0.041265 | 0.430378 | Down |
| Lanceotoxin B | 2.120708 | 0.001095 | 0.030008 | 0.38144 | Down |
| Leu-Leu-Ser-Pro-Tyr | 1.985507 | 0.001746 | 0.041265 | 0.234946 | Down |
| Lolitriol | 1.908876 | 0.001031 | 0.028464 | 0.17114 | Down |
| Lys-Leu-Glu-Ala-Thr | 2.323621 | 5.65E-05 | 0.003073 | 0.293577 | Down |
| Met-Phe-Val | 1.468576 | 0.001233 | 0.032764 | 0.209514 | Down |
| Obliquine | 1.765876 | 7.62E-05 | 0.003905 | 0.491682 | Down |
| Pennigritrem | 1.958547 | 0.001308 | 0.033948 | 0.389094 | Down |
| Phe4Cl-TyrMe-OH | 3.859647 | 6.88E-17 | 6.79E-14 | 0.354762 | Down |
| Phorbol 12-tiglate 13-decanoate | 1.619107 | 0.001386 | 0.035411 | 0.344575 | Down |
| Pro-Tyr-Pro-Arg-Ser | 1.899128 | 0.000404 | 0.014693 | 0.332072 | Down |
| Psychotridine | 2.150353 | 0.000971 | 0.027377 | 0.305976 | Down |
| Ser-Glu-Lys-Ile-Asp | 2.133356 | 0.001233 | 0.032764 | 0.401549 | Down |
| Ser-Glu-Val-Lys-Met | 1.872518 | 0.001469 | 0.036817 | 0.376797 | Down |
| Ser-Ile-Tyr | 2.410904 | 0.000809 | 0.023924 | 0.479889 | Down |
| Symlandine | 1.661346 | 0.001308 | 0.033948 | 0.496162 | Down |
| Thr-Lys-Met-Val-Glu | 2.111867 | 0.000715 | 0.02232 | 0.321548 | Down |
| Thr-Val-Thr-Phe-Tyr | 1.914183 | 0.000431 | 0.015395 | 0.300584 | Down |
| Tyr-Gln-Ile-Arg | 2.265681 | 0.001031 | 0.028464 | 0.332421 | Down |
| Tyr-Glu-Arg-Arg | 1.631162 | 0.001956 | 0.044621 | 0.431459 | Down |
| Tyr-Phe-Asn | 2.066362 | 0.000207 | 0.00883 | 0.194532 | Down |
| Tyr-Phe-His-Glu | 2.385175 | 7.07E-05 | 0.003673 | 0.273699 | Down |
| TyrMe-Met-OH | 2.603786 | 3.85E-05 | 0.002232 | 0.448797 | Down |
| Urdamycin B | 1.87896 | 0.00086 | 0.02515 | 0.345587 | Down |
| Urobilin | 2.180437 | 0.002188 | 0.048518 | 0.349209 | Down |
| Val-Trp | 1.316827 | 0.000254 | 0.010225 | 0.279838 | Down |
| Yangambin | 2.354111 | 0.000593 | 0.01911 | 0.442331 | Down |
| Debromoaplysiatoxin | 2.113153 | 0.001649 | 0.039562 | 0.284988 | Down |
| Catechol pentaacetate | 3.962229 | 1.34E-14 | 7.56E-12 | 0.353577 | Down |
| Diacerein | 1.167677 | 0.000809 | 0.023924 | 19.80374 | Up |
| Tridecanedioic acid | 4.321689 | 3.10E-13 | 1.29E-10 | 2.363735 | Up |
| Diflufenican | 1.864767 | 0.000332 | 0.01253 | 23.56356 | Up |
| Fosphenytoin | 1.177769 | 0.001469 | 0.036817 | 50.72048 | Up |
| Joro spider toxin | 2.256525 | 8.09E-06 | 0.000591 | 2.277674 | Up |
| Rebamipide | 1.544823 | 0.001469 | 0.036817 | 16.70012 | Up |
| Rugulosin | 1.783089 | 0.000404 | 0.014693 | 3.305892 | Up |
| 1-Arachidoyl-2-hydroxy-sn-glycero-3-phosphocholine | 3.002812 | 3.21E-10 | 5.51E-08 | 4.915114 | Up |
| 1-Oleoyl-2-acetyl-sn-glycerol | 2.936975 | 9.50E-05 | 0.004716 | 2.228685 | Up |
| 3-methoxy Limaprost | 3.361342 | 1.70E-10 | 3.05E-08 | 2.112748 | Up |
| Cymarin | 1.877846 | 0.000332 | 0.01253 | 18.27557 | Up |
| Dicloxacillin | 2.659859 | 0.00046 | 0.016058 | 5.200335 | Up |
| L-Homocysteic acid | 1.510145 | 2.09E-06 | 0.000196 | 26.08741 | Up |
| Pro-Ala | 2.375555 | 0.000809 | 0.023924 | 30.35134 | Up |
| D-Glucose 6-Phosphate | 1.760482 | 0.001557 | 0.038281 | 9.754227 | Up |
| 2-methyl-3-Pyrimidin-2-yl-Propionic Acid | 4.1928 | 1.58E-12 | 4.47E-10 | 2.375451 | Up |
| 3-[3-tert-Butylthio-1-(4-chlorobenzyl)-5-isopropyl-1H-indol-2-yl]-2,2-dimethylpropionic acid,sodium salt hydrate | 1.985793 | 0.000672 | 0.021227 | 21.34804 | Up |
| 5-Acetylamino-6-formylamino-3-methyluracil | 1.870198 | 0.00029 | 0.011349 | 6.524075 | Up |
| Clorazepate | 1.914662 | 0.001956 | 0.044621 | 3.35058 | Up |
| Decitabine | 1.271177 | 0.000971 | 0.027377 | 2.520982 | Up |
| Khellin | 1.799466 | 2.81E-05 | 0.001717 | 2.946987 | Up |
| [6-(5,7-dihydroxy-4-oxo-2-phenyl-4H-chromen-8-yl)-4,5-dihydroxyoxan-3-yl]oxidanesulfonic acid | 1.320256 | 0.001649 | 0.039562 | 3.633532 | Up |
| {2-[5,7-dihydroxy-2-(3-hydroxy-4-methoxyphenyl)-4-oxo-3-[3,4,5-trihydroxy-6-(hydroxymethyl)oxan-2-yl]-4H-chromen-8-yl]-4,5-dihydroxy-6-(hydroxymethyl)oxan-3-yl}oxidanesulfonic acid | 1.237806 | 0.000715 | 0.02232 | 40.50543 | Up |
| 5-({5-[(1-hydroxy-2-methylbut-3-en-2-yl)oxy]-2,2-dimethyl-7-(2-methylbut-3-en-2-yl)-8-oxo-2H,8H-pyrano[3,2-g]chromen-10-yl}oxy)-8,8-dimethyl-3,10-bis(2-methylbut-3-en-2-yl)-2H,8H-pyrano[3,2-g]chromen-2-one | 1.128942 | 0.000761 | 0.023104 | 3.478307 | Up |
| Acetylsalicylsalicylic acid | 2.215768 | 0.000593 | 0.01911 | 20.79521 | Up |
| Salvianolic acid A | 1.781503 | 2.39E-05 | 0.00151 | 3.593457 | Up |
| Sulfuretin | 1.403157 | 0.000254 | 0.010225 | 4.4141 | Up |
| 2-Octaprenyl-3-methyl-5-hydroxy-6-methoxy-1,4-benzoquinone | 2.655788 | 3.04E-05 | 0.001817 | 2.385352 | Up |
| 3-Nonaprenyl-4-hydroxybenzoate | 2.663987 | 0.00046 | 0.016058 | 2.6537 | Up |
| Bryophyllin A | 1.884067 | 0.000914 | 0.026336 | 11.43069 | Up |
| Cannabisativine | 2.787628 | 4.85E-05 | 0.002714 | 2.241294 | Up |
| Gln-Val-Leu-Leu-Gly | 1.59647 | 0.000168 | 0.007627 | 24.30244 | Up |
| Lys-Leu-Gly-Gln-Ser | 1.006699 | 0.000168 | 0.007627 | 81.68967 | Up |
| 3,4-(Methylenedioxy)cinnamic acid | 1.320091 | 0.001746 | 0.041265 | 6.466898 | Up |
| Patellamide A | 1.725901 | 0.000272 | 0.010828 | 7.791349 | Up |
| Phe-Lys-Glu-Lys | 1.493749 | 0.000557 | 0.018314 | 9.795357 | Up |
| Zhebeinine | 1.207688 | 1.73E-05 | 0.001146 | 6.809145 | Up |

Table S5 The metabolites differentially abundant between AS and HLA-B27(+) HC

| Compounds | VIP | P value | FDR | Fold Change  (AS/HLA-B27(+) HC) | Change |
| --- | --- | --- | --- | --- | --- |
| Tropisetron HCl | 2.085896 | 0.000523 | 0.031976 | 0.436408 | Down |
| Sarpogrelate hydrochloride | 2.043217 | 0.000431 | 0.030108 | 0.364653 | Down |
| 2-Amino-3-methoxybenzoic acid | 4.18802 | 8.26E-16 | 3.26E-12 | 0.304964 | Down |
| Pectolinarigenin | 4.27165 | 2.06E-15 | 5.43E-12 | 0.494231 | Down |
| (±)9-HpODE | 1.798818 | 0.00046 | 0.031557 | 0.390343 | Down |
| (S)-O-Desmethyl Naproxen | 3.40331 | 2.94E-08 | 1.79E-05 | 0.427742 | Down |
| (±)14(15)-EpETE | 2.073843 | 0.000632 | 0.035358 | 0.472231 | Down |
| 2-Naphthalenesulfonic acid | 2.378892 | 1.22E-10 | 1.07E-07 | 0.476291 | Down |
| 1-Hydroxy-1-(4-methoxyphenyl)propan-2-yl 4-methoxybenzoate | 2.02688 | 0.000715 | 0.037151 | 0.498393 | Down |
| 8-Anilino-1-naphthalenesulfonic acid | 4.254992 | 3.50E-14 | 6.90E-11 | 0.475579 | Down |
| Dehydrochorismic acid | 2.359751 | 1.70E-10 | 1.34E-07 | 0.483406 | Down |
| Manidipine | 2.296194 | 0.000971 | 0.04431 | 0.404917 | Down |
| Taprostene | 2.183552 | 0.000431 | 0.030108 | 0.380992 | Down |
| (3E)-4-(1-Hydroxy-2,6,6-trimethyl-4-oxocyclohex-2-en-1-yl)but-3-en-2-yl 6-O-pentopyranosylhexopyranoside | 2.562012 | 0.00049 | 0.031976 | 0.462629 | Down |
| 3alpha-Acetomethoxy-11alpha-oxo-12-ursen-24-oic acid | 1.627051 | 0.000672 | 0.037151 | 0.211908 | Down |
| 3-Methylglutarylcarnitine | 2.390831 | 0.000118 | 0.012588 | 0.348342 | Down |
| 7-Hydroxypimara-8(14),15-dien-18-oic acid | 2.422831 | 0.000354 | 0.026641 | 0.440661 | Down |
| 9(10)-Epoxy-12Z-octadecenoic acid | 2.524369 | 0.00018 | 0.017785 | 0.367607 | Down |
| 9(S)-HODE | 2.485122 | 0.000378 | 0.027916 | 0.375133 | Down |
| Farnesyl acetate | 2.146995 | 0.000632 | 0.035358 | 0.444195 | Down |
| Khivorin | 1.724465 | 0.001095 | 0.047486 | 0.38874 | Down |
| Asn-Lys | 2.466065 | 0.00029 | 0.023635 | 0.495335 | Down |
| Bestatin(hydrochloride) | 1.818023 | 0.000593 | 0.034174 | 0.405193 | Down |
| Cibulins | 3.81152 | 3.01E-12 | 3.40E-09 | 0.433594 | Down |
| Enalapril | 1.397681 | 0.000593 | 0.034174 | 0.248441 | Down |
| Glutethimide | 2.340501 | 0.000523 | 0.031976 | 0.420108 | Down |
| Gly-Pro-Lys | 2.170655 | 0.000971 | 0.04431 | 0.302756 | Down |
| Leu-Tyr | 2.176292 | 2.03E-05 | 0.003649 | 0.367958 | Down |
| Lysyl-Lysine | 2.606948 | 0.00011 | 0.011874 | 0.453555 | Down |
| Methotrexate | 2.704708 | 3.29E-05 | 0.005297 | 0.333852 | Down |
| Prilocaine | 1.83855 | 1.59E-05 | 0.003139 | 0.305848 | Down |
| Ternatin | 1.49274 | 0.001031 | 0.046519 | 0.242851 | Down |
| Tyrosyl-Leucine | 2.356446 | 0.00031 | 0.024507 | 0.45199 | Down |
| L-Arabinitol | 2.281802 | 0.000593 | 0.034174 | 0.452351 | Down |
| 8-Hydroxydesmethylclomipramine | 2.081803 | 2.39E-05 | 0.004195 | 0.417823 | Down |
| Cyclothiazide | 3.69958 | 2.57E-11 | 2.54E-08 | 0.304551 | Down |
| Dulciol C | 2.717043 | 8.83E-05 | 0.010249 | 0.429755 | Down |
| Mequitazine | 2.628944 | 0.000254 | 0.022268 | 0.490804 | Down |
| Pelitinib | 2.51944 | 0.000193 | 0.018595 | 0.485807 | Down |
| 1,7-Bis(4-hydroxyphenyl)-5-methoxyheptan-3-one | 2.482868 | 0.000971 | 0.04431 | 0.482882 | Down |
| 3',4'-Methylenedioxyorobol | 3.836438 | 2.75E-16 | 2.17E-12 | 0.486239 | Down |
| 6-{4-[3-(3,7-dimethylocta-2,6-dien-1-yl)-7-hydroxy-8-(3-methylbut-2-en-1-yl)-4-oxo-4H-chromen-2-yl]-3-hydroxyphenoxy}-3,4,5-trihydroxyoxane-2-carboxylic acid | 2.132603 | 2.81E-05 | 0.004816 | 0.374501 | Down |
| Rutamarin | 2.809162 | 1.24E-05 | 0.002507 | 0.490047 | Down |
| Thalsimine | 1.896979 | 0.00049 | 0.031976 | 0.282751 | Down |
| (9Z)-(7S,8S)-Dihydroxyoctadecenoic acid | 1.776436 | 0.000254 | 0.022268 | 0.433326 | Down |
| 7(1)-Hydroxychlorophyllide a | 2.16006 | 4.85E-05 | 0.006957 | 0.329879 | Down |
| Ala-Asp-Phe-Asp | 1.699069 | 0.00018 | 0.017785 | 0.495681 | Down |
| Ardisianone | 1.648624 | 0.000523 | 0.031976 | 0.207472 | Down |
| Asn-His-Val-Asp | 1.931571 | 0.000254 | 0.022268 | 0.191654 | Down |
| Asp-Leu-Ser-Glu | 2.041651 | 0.000557 | 0.033298 | 0.362356 | Down |
| Cannabisin D | 2.701444 | 3.04E-05 | 0.005103 | 0.396386 | Down |
| Codonocarpine | 1.900208 | 0.00031 | 0.024507 | 0.349926 | Down |
| Cryptopleurine | 1.52844 | 0.000431 | 0.030108 | 0.263258 | Down |
| His-Tyr-Gln-Asp | 1.431816 | 0.000809 | 0.039923 | 0.216901 | Down |
| JWH147 | 2.569469 | 7.41E-06 | 0.001721 | 0.344797 | Down |
| Leu-Ser-Phe-Met-Gln | 2.664823 | 0.000272 | 0.022808 | 0.45192 | Down |
| Lolitriol | 1.913862 | 0.001095 | 0.047486 | 0.214297 | Down |
| Lys-Leu-Glu-Ala-Thr | 2.529688 | 0.000523 | 0.031976 | 0.316822 | Down |
| Mycolactone D | 1.544501 | 0.000193 | 0.018595 | 0.237753 | Down |
| Thr-Lys-Met-Val-Glu | 2.063735 | 0.000431 | 0.030108 | 0.421928 | Down |
| Tyr-Phe | 2.057795 | 0.00011 | 0.011874 | 0.47438 | Down |
| Val-Glu-Ser-Lys | 2.372595 | 0.00011 | 0.011874 | 0.408983 | Down |
| CAFESTOL ACETATE | 1.756545 | 0.001162 | 0.049578 | 0.437169 | Down |
| Diacerein | 1.509402 | 1.24E-05 | 0.002507 | 38.93386 | Up |
| Deoxyrubroskyrin | 1.655179 | 0.00046 | 0.031557 | 2.524875 | Up |
| 1-Arachidoyl-2-hydroxy-sn-glycero-3-phosphocholine | 2.055532 | 3.03E-06 | 0.000854 | 2.840324 | Up |
| Lippioside II | 1.554589 | 0.000715 | 0.037151 | 2.788492 | Up |
| 3-[3-tert-Butylthio-1-(4-chlorobenzyl)-5-isopropyl-1H-indol-2-yl]-2,2-dimethylpropionic acid,sodium salt hydrate | 2.574681 | 0.000971 | 0.04431 | 20.59122 | Up |
| 3-Methylpyrrole-2,4-dicarboxylic Acid | 2.266534 | 0.000354 | 0.026641 | 70.45464 | Up |
| Chelidonic acid | 2.801807 | 0.000523 | 0.031976 | 28.45578 | Up |
| Khellin | 1.74359 | 8.09E-06 | 0.001824 | 3.034339 | Up |
| 5-({5-[(1-hydroxy-2-methylbut-3-en-2-yl)oxy]-2,2-dimethyl-7-(2-methylbut-3-en-2-yl)-8-oxo-2H,8H-pyrano[3,2-g]chromen-10-yl}oxy)-8,8-dimethyl-3,10-bis(2-methylbut-3-en-2-yl)-2H,8H-pyrano[3,2-g]chromen-2-one | 1.835394 | 0.000632 | 0.035358 | 6.197685 | Up |
| Sulfuretin | 1.402474 | 0.000127 | 0.013166 | 2.591806 | Up |
| Beiwutine | 2.535797 | 0.000971 | 0.04431 | 32.24375 | Up |
| Patellamide A | 1.83199 | 0.000354 | 0.026641 | 4.263793 | Up |
| Phe-Lys-Glu-Lys | 2.024313 | 0.001095 | 0.047486 | 23.56274 | Up |
| Scillaren A | 2.548981 | 0.001095 | 0.047486 | 30.74217 | Up |
| Thr-His-Ile-Arg | 1.729242 | 0.000971 | 0.04431 | 58.70162 | Up |
| Tyr-Leu-Thr-Arg | 2.472322 | 0.000715 | 0.037151 | 6.121333 | Up |
| alpha-Peltatin | 1.349916 | 0.001162 | 0.049578 | 3.596894 | Up |

Table S6 The metabolites differentially abundant between HLA-B27(+) and HLA-B27(-) HC

| Compounds | VIP | P value | FDR | Fold Change  (HLA-B27(+) /HLA-B27(-) HC) | Change |
| --- | --- | --- | --- | --- | --- |
| 4-O-8',5'-5''-Dehydrotriferulic acid | 3.73841 | 8.24E-08 | 2.50E-05 | 0.287159 | Down |
| Fusaroskyrin | 3.542298 | 1.57E-06 | 0.000309 | 0.292565 | Down |
| Sulochrin | 3.465899 | 3.14E-07 | 7.51E-05 | 0.498208 | Down |
| 1,2-Dioleoyl-sn-glycero-3-phosphoethanolamine-N-methyl | 2.922242 | 0.000153 | 0.013581 | 0.252286 | Down |
| 1,2-Ditetradecanoyl-sn-glycero-3-phosphocholine | 2.421062 | 4.37E-05 | 0.004423 | 0.428471 | Down |
| 2-Hydroxy-N-[(E)-3-hydroxy-1-[3,4,5-trihydroxy-6-(hydroxymethyl)oxan-2-yl]oxyoctadec-4-en-2-yl]hexadecanamide | 2.400927 | 0.000175 | 0.015162 | 0.365866 | Down |
| PC(16:0/20:5(5Z,8Z,11Z,14Z,17Z)) | 2.691175 | 4.79E-08 | 1.64E-05 | 0.206514 | Down |
| SM(d18:0/16:1(9Z)(OH)) | 3.13268 | 6.72E-05 | 0.006394 | 0.311452 | Down |
| Vaccinoside | 3.76806 | 6.64E-08 | 2.18E-05 | 0.279961 | Down |
| Cephalosporin C | 2.934077 | 4.06E-05 | 0.004164 | 0.48901 | Down |
| Cidofovir | 3.86511 | 4.58E-09 | 2.26E-06 | 0.261525 | Down |
| 4-(7-Hydroxy-4-oxo-3,4-dihydro-2H-chromen-2-yl)phenyl 2-O-(3,4-dihydroxy-4-(hydroxymethyl)tetrahydrofuran-2-yl)hexopyranoside | 1.829638 | 8.68E-06 | 0.001162 | 0.453111 | Down |
| Epoxiconazole | 3.928886 | 1.94E-08 | 8.06E-06 | 0.353735 | Down |
| Sophoraflavonoloside | 2.048217 | 2.80E-05 | 0.003073 | 0.462793 | Down |
| Ala-Leu-Gly-Ile-Ser | 1.697246 | 0.000143 | 0.012997 | 0.48852 | Down |
| Boc-Pro-Phe(NMe)-Gly-OMe | 2.663185 | 0.000543 | 0.041583 | 0.427276 | Down |
| Ile-Val-Tyr | 3.997878 | 5.63E-07 | 0.000127 | 0.466209 | Down |
| Phe4Cl-TyrMe-OH | 4.386259 | 1.43E-09 | 1.13E-06 | 0.479705 | Down |
| Val-Trp | 1.500161 | 0.0004 | 0.031912 | 0.345209 | Down |
| Joro spider toxin | 3.051214 | 1.02E-05 | 0.001343 | 2.219102 | Up |
| 2-Oxopentanoic acid | 4.135763 | 2.84E-07 | 7.01E-05 | 2.138386 | Up |
| 3-Nonaprenyl-4-hydroxybenzoate | 3.226925 | 0.000213 | 0.018246 | 2.058075 | Up |

Table S7 Key correlations of microbial species and metabolites with clinical parameters

| Clinical parameters | Species/metabolites | Spearman's rank correlation coefficient | P value |
| --- | --- | --- | --- |
| ASDAS-CRP | Tyr-Gln-Ile-Arg | -0.4982 | 0.009591 |
| ASDAS-CRP | Sedanolide | -0.4452 | 0.022659 |
| ASDAS-CRP | CID_57404059 | -0.38913 | 0.049437 |
| ASDAS-CRP | Lucidone B | -0.58643 | 0.001641 |
| ASDAS-CRP | Gly-Lys-Gln-Leu-Glu | -0.53616 | 0.004752 |
| ASDAS-CRP | Chivosazole B | -0.4028 | 0.041326 |
| ASDAS-CRP | CID_53387189 | -0.50368 | 0.008709 |
| ASDAS-CRP | *Tannerella sp. CAG:118* | 0.397572 | 0.044294 |
| ASDAS-CRP | *Bacteroides sp. CAG:714* | 0.398359 | 0.043837 |
| ASDAS-CRP | *Propionispora sp. 2_2-37* | 0.395691 | 0.045401 |
| BASDAI | Tyr-Gln-Ile-Arg | -0.49265 | 0.010561 |
| BASDAI | Sedanolide | -0.40103 | 0.042316 |
| BASDAI | CID_443295 | -0.51453 | 0.007159 |
| BASDAI | CID_57404059 | -0.46667 | 0.016248 |
| BASDAI | Lucidone B | -0.4735 | 0.014552 |
| BASDAI | Gly-Lys-Gln-Leu-Glu | -0.50359 | 0.008722 |
| BASDAI | CID_53387189 | -0.42701 | 0.029582 |
| BASDAI | *Propionispora sp. 2_2-37* | 0.388784 | 0.049654 |
| BASDAI | *Negativibacillus massiliensis* | 0.390769 | 0.048401 |
| BASFI | Cinnabarinic acid | -0.39739 | 0.044397 |
| BASFI | Tyr-Gln-Ile-Arg | -0.45608 | 0.019194 |
| BASFI | Sedanolide | -0.43103 | 0.027924 |
| BASFI | CID_443295 | -0.42862 | 0.028906 |
| BASFI | N-Acetylornithine | -0.56315 | 0.002741 |
| BASFI | Lucidone B | -0.44029 | 0.024383 |
| BASFI | Chivosazole B | -0.41627 | 0.034408 |
| BASFI | CID_53387189 | -0.57276 | 0.002228 |
| BASFI | *Bacteroidetes bacterium 41-46* | 0.453466 | 0.019984 |

Table S8 The selected species and metabolites for constructing random forest models

| Model 1 (Species) | Model 2 (Metabolites) | Model 3 (Combined) |
| --- | --- | --- |
| B27(-) VS AS | | |
| *Lachnospiraceae bacterium 1_1_57FAA* | CID 1369 | Model 1+Model 2 features |
| *Bacteroides sp. An19* | Cibulins |  |
| *Clostridium beijerinckii* |  |  |
| *Bacteroides sp. An51A* |  |  |
| *Bacteroidales bacterium 36-12* |  |  |
| B27(+) VS AS | | |
| *Lachnospiraceae bacterium 1_1_57FAA* | 3’,4’-Methylenedioxyorobol | Model 1+Model 2 features |
| *Oribacterium sp. oral taxon 108* | CID 1369 |  |
| *Cloacibacillus sp. An23* |  |  |
| *Geosporobacter ferrireducens* |  |  |
| *Bacteroides luti* |  |  |
| B27(-) VS B27(+) | | |
| *Bacteroides sp. An51A* | Pectolinarigenin | Model 1+Model 2 features |
| *Propionispora sp. 2_2-37* | CID 1369 |  |
| *Paraprevotella clara* | 3’,4’-Methylenedioxyorobol |  |
|  | N-Acetyltyramine |  |
|  | Cibulins |  |
